# Probiogenomic analysis of functional potential and safety of *L. plantarum* 8p-a3 and DMC-S1 strains: *in silico* vs *in vitro* and *in vivo* data

**DOI:** 10.1128/spectrum.02314-25

**Published:** 2026-07-06

**Authors:** Maria Markelova, Victoria Kostenko, Arina Aristova, Maria Nikolaeva, Maya Kharitonova, Shamil Validov, Airat Kayumov, Olga Chernova, Vladislav Chernov

**Affiliations:** 1 Institute of Fundamental Medicine and Biology, Kazan Federal University 64922, Kazan, Russia Federation; 2 Kazan Institute of Biochemistry and Biophysics, FRC Kazan Scientific Center of Russian Academy of Sciences 242112 , Kazan, Russian Federation; 3 Laboratory of Molecular Genetics and Microbiology Methods, FRC Kazan Scientific Center of Russian Academy of Sciences 104639, Kazan, Russian Federation; Karolinska Institutet, Stockholm, Sweden

**Keywords:** probiotics, functionality, safety, whole-genome analysis, risk factors, antimicrobial resistance, extracellular vesicles, *in vitro and in vivo *assays

## Abstract

The molecular basis of the beneficial effects and the causes of the negative effects of probiotics are not entirely clear. Clarifying these issues is important for understanding the biology and assessing the safety of the microbes. Omics technologies have opened up new resources for obtaining relevant knowledge. Here, for the first time, we present the results of a comparative analysis of the functional potential and safety of two *L. plantarum strains*: the approved probiotic 8p-a3 and the *Drosophila* intestinal resident, which exhibit opposite effects on *D. melanogaster* as the model host organism. Through genomic analysis, extracellular vesicle studies, and *in vitro* and *in vivo* assays, we have identified the common and specific characteristics of the strains. The strains proved to be similar in a set of genes that determine benefits to the host organism, as well as in the presence of some risk factors. Significant differences between the strains are related to genes responsible for adhesion, sialic acid metabolism, mucin degradation, antimicrobial peptides, tannin resistance, and immunomodulation. *In silico* data correlated with in *vitro and in vivo data*, with the exception of antimicrobial sensitivity. Pronounced differences between the strains were found in terms of the composition and biological effects of their vesicles. *In vivo* data on the effects of the strains correlate with the corresponding data of their vesicles in the fruit fly model. The results obtained open up new facets in *L. plantarum* strains relevant for evaluating the functionality and safety of probiotics.

**IMPORTANCE:**

Using a probiogenomic approach, common and specific features regarding functionality and safety were identified in the strains (the approved probiotic strain *L. plantarum* 8p-a3 and the *Drosophila* intestinal bacterium *L. plantarum* DMC-S1), which exhibit opposite effects on the model host organism (*D. melanogaster*). The genomic analysis was supplemented by the analysis of extracellular vesicles of the strains. Comparative analysis of *in silico* data in combination with *in vitro* and *in vivo* studies was performed, and unexpected capabilities of the strains were discovered. Novel factors, essential for evaluating the safety of probiotics, were identified. New facets in the interplay of probiotic bacterium with host organism have been revealed.

## INTRODUCTION

Probiotics are attracting increasing attention due to their ability to positively influence intestinal homeostasis and the immune system of the host, regulate the structure of microbial communities, and inhibit the growth of pathogenic bacteria (1). The antimicrobial activity of these microorganisms is especially relevant nowadays, given the rapid spread of antibiotic resistance and the antimicrobial drug crisis background (2). The creation of new-generation antimicrobials, immunomodulators, and precision postbiotics is associated with bacteria with GRAS (Generally Recognized As Safe) status, in particular probiotic strains. Specific compounds produced by bacteria (antimicrobial peptides), as well as bacterial extracellular vesicles transporting bioactive molecules and mediating intercellular communication and interactions of bacteria with pro- and eukaryotic cells, are considered candidates for the role of novel postbiotics (3–5). The realization of the relevant prospects involves a comprehensive analysis of the strains’ efficiency, both in terms of functionality and safety. Meanwhile, the molecular mechanisms of interplay between probiotic bacteria and cells of micro- and macroorganisms, including those providing beneficial properties for the host organism, remain largely unknown (6, 7). Moreover, while differential outcomes of the interaction of various probiotic strains with the host organism, as well as the manifestation of negative effects of probiotics, have been reported, the causes of these phenomena remain unclear (6, 8). The lack of standardization of the analysis of probiotics (and probiotic candidates) is a significant obstacle to addressing these issues (1).

The development of high-resolution methods, advancing genomic and postgenomic technologies, and progressing bioinformatic tools has determined the emergence of a new scientific field named probiogenomics (9, 10). This area aims to ensure that the potential of omics technologies, along with the capabilities of classical microbiology, is used for the comprehensive characterization of probiotic strains. The new knowledge gained deepens our understanding of the biology of bacteria and advances probiogenomics. The establishment of the fact that extracellular vesicles are key intermediaries of bacterial interaction with cells of macro- and microorganisms determines the relevance of analyzing the properties of these nanostructures for understanding the biology of intestinal commensals/symbionts, as well as for evaluating the functionality and safety of probiotic strains (3, 11).

Genome-wide analysis of strains and targeted *in vitro*/*ex vivo*/*in vivo* studies are used today to assess crucial elements—probiotic features and risk factors in “probiotic candidates” (10). Such analysis is also relevant for long-used commercial probiotics, especially if negative effects have been reported for them.

We have shown previously that the approved probiotic strain *Lactiplantibacillus plantarum* 8p-a3 (isolated from the commercial probiotic drug Lactobacterin) exhibits negative effects against the model host organism *Drosophila melanogaster* (Canton-S) (12). On the contrary, the strain isolated from *D. melanogaster* intestine, *L. plantarum* DMC-S1, exhibits an exceptionally positive effect on the host. The existence of two phylogenetically close strains that differentially affect the host, in combination with probiogenomics tools, opens a unique possibility to reveal genetic features determining the observed effects and to conduct a comparative study of these bacteria from the point of view of their functionality, safety, and identification of candidate factors associated with the negative effects of the probiotic strain.

Here, we present a comparative analysis of *in silico*, *in vitro*, and *in vivo* data reflecting the functional potential and safety of two *L. plantarum* strains: the approved probiotic 8p-a3 and the *Drosophila* gut resident DMC-S1, which exhibit opposite effects on the model host organism *D. melanogaster* (Canton-S). The genome-wide analysis of the strains, supplemented with data characterizing features and biological activity of the strains’ vesicles, allowed the identification of novel factors essential for evaluating the functionality and safety of probiotics.

## RESULTS

### The general properties of genomes of *L. plantarum* strains

**Fig 1:**
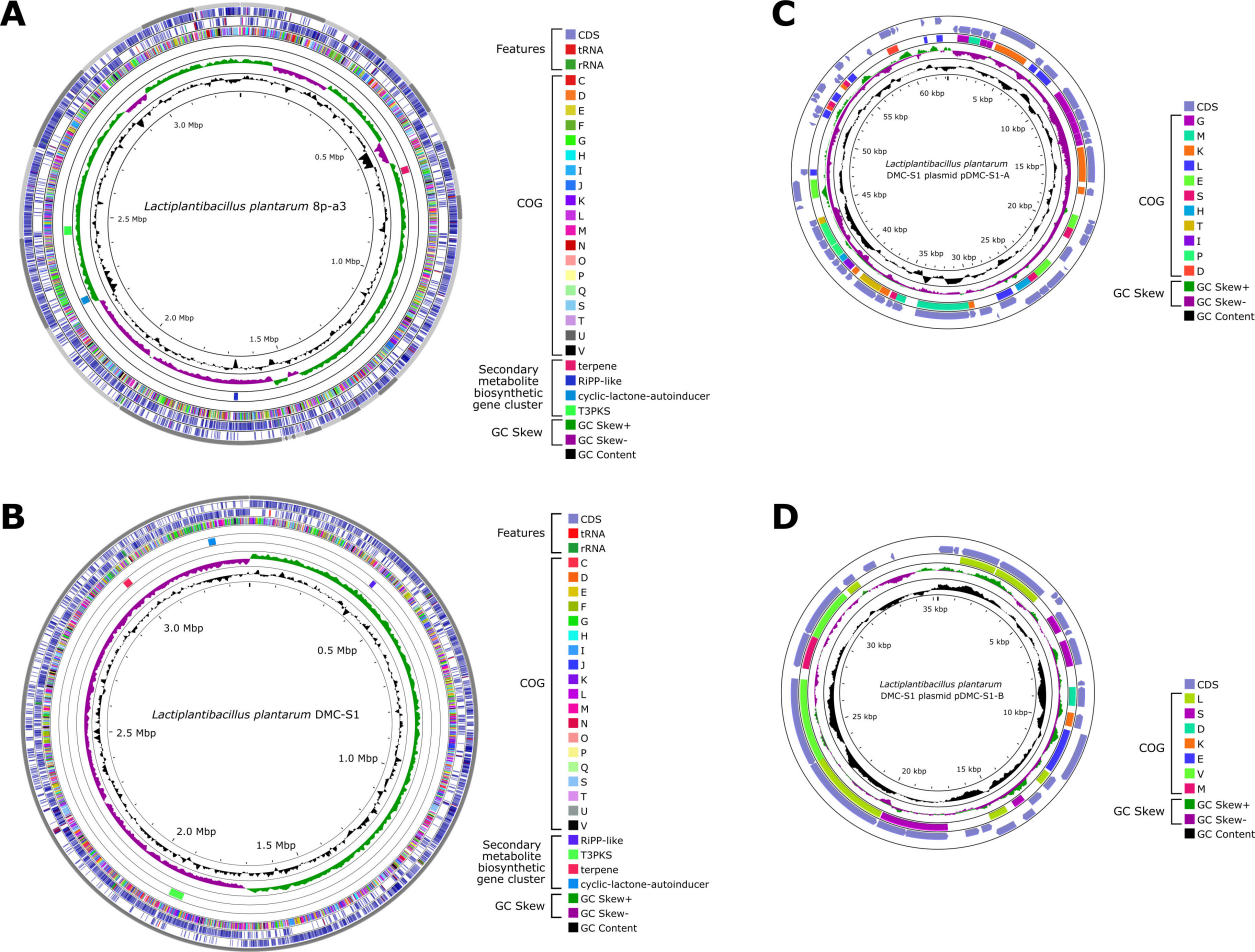
Circular maps of *L. plantarum* 8p-a3 and DMC-S1 genomes visualized using the Proksee tool. (**A** **and B**) Chromosomes of *L. plantarum* 8p-a3 and DMC-S1, respectively*.* (**C and** **D**) Plasmids of *L. plantarum* DMC-S1. See COG category abbreviations in Table S1*.*

The whole genome of *L. plantarum* DMC-S1 has been sequenced using Illumina MiSeq and Oxford Nanopore MinION platforms with an overall coverage of 180× (75× on Illumina MiSeq and 105× on Oxford Nanopore MinION) and sequence reads quality of 99.9%. The *L. plantarum* DMC-S1 genome includes a circular chromosome consisting of 3,376,068 bp and a GC content of 44.5% (Table 1; Fig. 1), and two plasmids with a total length of 63,256 and 35,273 base pairs, respectively (2.92% of the genome), and GC contents of 37.5% and 39.5%. In total, the genome contains 3,349 predicted open reading frames, as well as genes encoding six copies of 5S rRNA, five copies of 16S rRNA, five copies of 23S rRNA, 82 tRNA genes, and four ncRNA molecules.

**TABLE 1:** Comparison of *L. plantarum* 8p-a3 and DMC-S1 genome features^*a*^

	*L. plantarum* 8p-a3*	*L. plantarum* DMC-S1
Genome length (bp)	3,301,244	3,376,068
GC content (%)	44.5	44.5
Plasmids	ND (**)	2 (63,256 bp and 35,273 bp)
Number of ORF	3,154	3,349
5S rRNA	5	6
16S rRNA	2	5
23S rRNA	1	5
tRNA	73	82
ncRNA	4	4

^*a*^ (*) ANI *L. plantarum* 8p-a3 and *L. plantarum *8P-A3 (GCF_009762745.1), 99.997%; ND, no data; (**) plasmid of *L. plantarum *8P-A3, 9,480 bp.

The genome of *L. plantarum* 8p-a3 has been sequenced earlier with an overall coverage of 175× (12). The chromosomal DNA consists of 43 scaffolds with the total length of 3,301,244 base pairs and the GC content of 44.5% (Table 1; Fig. 1). The genome contains 3,154 predicted open reading frames, as well as genes of five copies of 5S rRNA, two copies of 16S rRNA, one copy of 23S rRNA, 73 tRNA genes, and four ncRNA molecules. The main characteristics of the 8p-a3 strain genome correspond to those of strain 8P-A3 (GenBank NCBI CP046726; CP046727), isolated from the commercial probiotic drug Lactobacterin and analyzed by independent researchers. *L. plantarum* 8P-A3 also carries a plasmid DNA of 9,480 bp in length and a GC content of 44.5%. The average nucleotide identity (ANI) between *L. plantarum* 8p-a3 and 8P-A3 is 99.997%. In total, 50 mutations differentiating these strains from each other have been confirmed by both raw whole genome and read alignment, including 14 (28.0%) mutations in the intergenic region, 22 (44.0%) in genes encoding hypothetical proteins, and 14 (28.0%) in genes with a known function (*msrA*, *rbsR, rpoN*, *rpoD*, *mltG*, *atpF*, genes encoding 2-oxoacid dehydrogenase subunit E2, ABC transporter permease, C40 family peptidase, Cof-type HAD-IIB family hydrolase, Dps family protein, LysR family transcription regulator, and uracil-xanthine permease family protein) (see ST_snp.xlsx). Thus, the strains 8p-a3 and 8P-A3 are very close, which allows data to be extrapolated.

### Functional annotation of genomes

**Fig 2:**
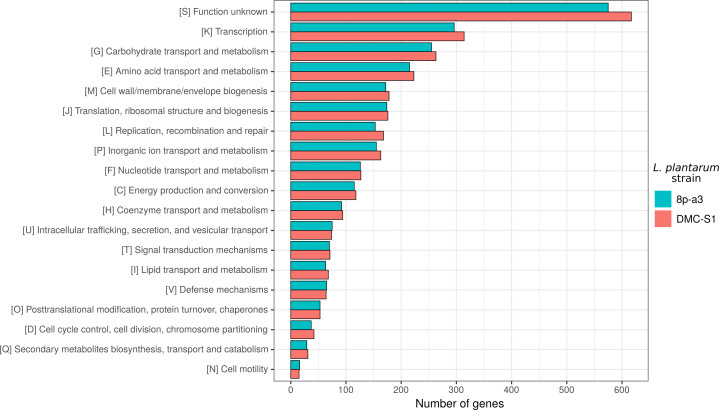
Comparison of distribution of COG (Cluster of Orthologous Groups) functional categories of the proteins in *L. plantarum* 8p-a3 and DMC-S1 (according to COG annotation).

**Fig 3:**
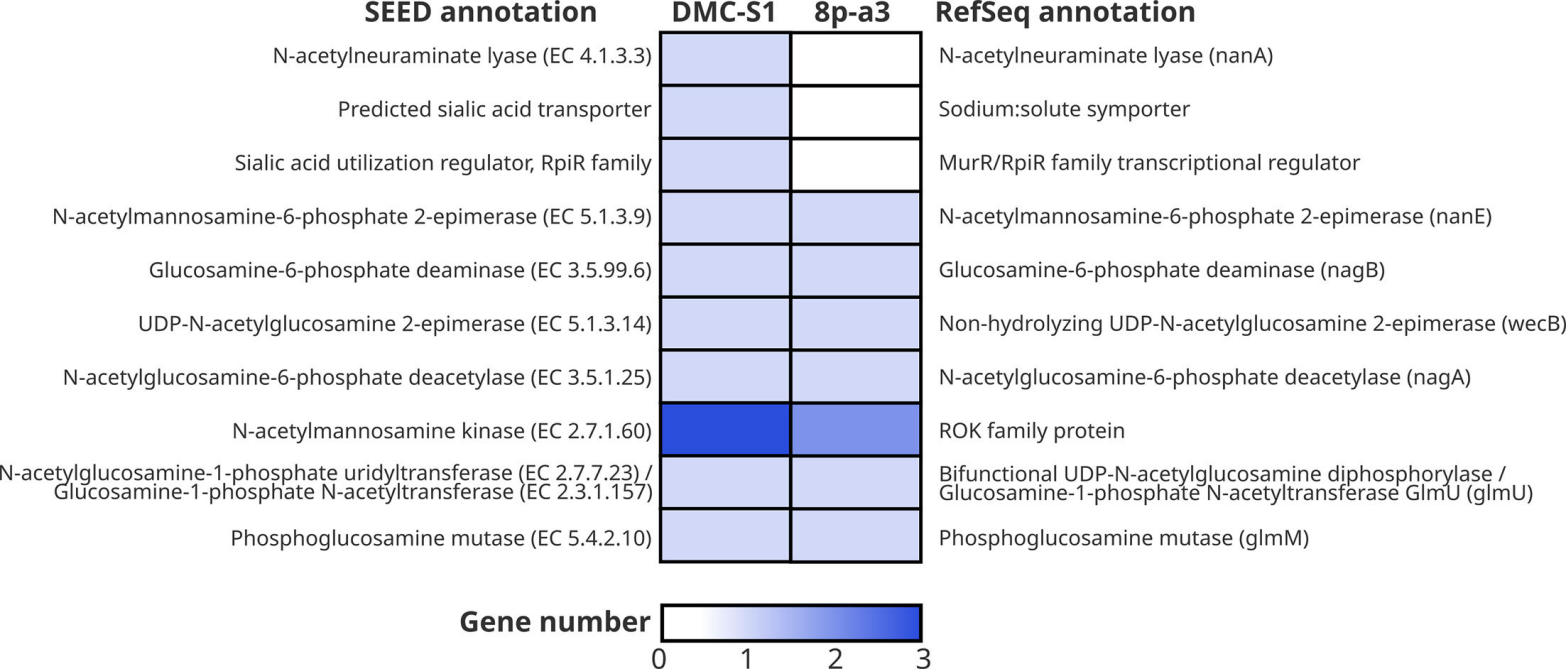
The presence of genes for sialic acid metabolism in *L. plantarum* DMC-S1 and 8p-a3 (according to SEED annotation).

To evaluate the functional potential of *L. plantarum* strains, their genomes and gene functions have been annotated using SEED, COG, KEGG, Pfam, and TCDB databases. Results are summarized in Table 2; Tables S1 to S3 and Fig. 2. Most genes are involved in the metabolism of carbohydrates and amino acids, genetic machinery, and environmental information processing. A substantial fraction of genes belongs to the category of “unknown function.” Both strains have pathways for the synthesis of vitamins (riboflavin, thiamine, pyridoxine, and folic acid), which are important for the metabolism of the host, the lactic acid bacterium itself, and other bacteria in the consortium. While the genes for sialic acid metabolism were found in both genomes, three genes associated with the catabolism of sialic acids (N-acetylneuraminate lyase, putative sialic acid transporter, and sialic acid utilization regulator) were found in the genome of *L. plantarum* DMC-S1 and were absent in the 8p-a3 strain (Fig. 3). Thus, the functional annotation of genomes indicates high similarity among strains in terms of functional potential, including the pool of genes relevant for survival under critical conditions and the implementation of functions beneficial to the host. At the same time, both strains contain genes for sialic acid catabolism, although with some differences, suggesting an advantage of *L. plantarum* DMC-S1.

**TABLE 2:** Functional profiling of *L. plantarum* 8p-a3 and DMC-S1 genomes^*a*^

	Database				
	COG	KEGG	SEED	Pfam	TCDB
*L. plantarum* 8p-a3	2,554	1,259	930	2,528	368
*L. plantarum* DMC-S1	2,665	1,284	970	2,618	389

^*a*^ In case of multiple annotation results, the best ratio is chosen for gene annotation.

### Safety assessment of strains *in silico* and *in vitro*

#### Genome instability

The genomes of *L. plantarum* 8p-a3 and DMC-S1 contained features associated with genomic instability, including IS, transposons (Table S4), prophages (Table S5), plasmids (Tables S6 to S8), CRISPR-Cas systems (Table S9), and genomic islands (ST_islands.xlsx). The genes within genomic islands are associated with bacteriocins, prophages, insertion elements, transcriptional regulation, transporters, and virulence, in particular, hemolysins of the XhlA family. While CRISPR/Cas systems of bacteria contribute to their protection from viruses, CRISPR/Cas systems in both strains have been identified with an index of 1.0, indicating their apparent inactivity (Table S9), increasing the possibility of foreign DNA acquisitions associated with horizontal transfer, virulence factors, and antibiotic resistance determinants. These data suggest similarity between the strains in terms of the potential for genomic instability.

#### Hemolysins and toxic metabolites

Using the RefSeq annotation results, genes for hemolysins of the XhlA family have been identified in both genomes. Therefore, the expression of these genes was evaluated in *vitro* by qRT-PCR (Fig. S1). A twofold higher expression of the *хhlA* gene at 37°C than at 25°C has been observed in both strains, suggesting their hemolytic potential. Furthermore, the appearance of a greenish halo around both strains, 8p-a3 and DMC-S1, has been observed on sheep blood agar (Fig. S2), confirming α-hemolytic activity, i.e., the ability to cause partial degradation of erythrocytes, similarly to *S. sobrinus,* which served as a reference.

The potential of *Lactobacilli* to produce metabolites that are potentially toxic for humans was evaluated via BLASTn search for the respective genes. While the genes of biogenic amine synthesis were not detected in any strain, both genomes contained the key genes for D-lactate synthesis and genes associated with nitro compound formation (Table S10). Moreover, while the qRT-PCR data indicated the constitutive expression of genes for D-lactate synthesis in cells of both strains, their expression differs between the strains and depends on the temperature, significantly increasing at 37°C in the probiotic strain. The data obtained indicate that the strains studied are similar in terms of “potential genomic liabilities” requiring further validation.

#### Determinants of antibiotic resistance

Using RGI and CARD, the *dltABCD* and *mprF* genes responsible for resistance to cationic antimicrobial peptides have been identified in both genomes. While other genes associated with AMR were not detected in both strains at a high probability threshold (Table S11), both strains were resistant to streptomycin, aminoglycosides, tetracycline, cephalosporins (except ceftazidime for *L. plantarum* 8p-a3), vancomycin, and fluoroquinolones, and were sensitive to amoxicillin, ampicillin, carbapenems, and macrolides (Table S12).

#### Virulence and persistence factors

While VirulenceFinder did not identify any virulence genes in both genomes, BLASTn and VFDB revealed a number of virulence-associated genes (Table S13), although with low identity. Some marker genes found in probiotic strains encoding adhesins and immunomodulators may also ensure the bacterial survival and persistence (are associated with virulence in pathogens), as well as expansion under stressful conditions (Tables S14 and S15).

While the strains differ only slightly in genes required for persistence, significant differences are found in the profiles of adhesin genes *cwaA*, *msa*, *mub*, and LPXTG. Of note, proteins containing LPXTG motif are considered essential adhesins, and the *msa* is a marker of probiotic strains. *L. plantarum* 8p-a3 has a genotype of *cwaA^+^ msa^−^ mub^+^* LPXTG^5+^, and *L. plantarum* DMC-S1 is *cwaA^−^ msa^+^ mub*^−^ LPXTG^6+^, thus being expected to differ in adhesion and autoaggregation abilities. These data matched the results of *in vitro* tests (Fig. S3 and S4): the DMC-S1 strain exhibited a higher rate of autoaggregation compared to 8p-a3 (78% vs 62%) and twofold higher hydrophobicity.

Significant differences between the strains are found in the set of genes for tannin resistance. In addition to the *tanB* gene, the probiotic strain 8p-a3 contains the *tanA* gene *encoding* a rare subtype of tannase, which may provide bacteria with unique opportunities for stress survival. The gene has a constitutive expression while significantly increased at 37°C (Fig. S1).

The elucidation of the role of AckA and MprF in immunomodulation, which is critical for the outcome of the interaction of *L. plantarum* with a model organism (*D. melanogaster*), requires the need for attention to the gene sequences of the corresponding proteins. The strains studied proved to carry different variants of the *ackA* and *mprF* genes (Fig. S8 and S9), and the expression patterns of these genes differ between the strains, and depend on the temperature, significantly increasing at 37°C in probiotic strain (Fig. S1).

Thus, strains differ by the pattern of genes required for bacterial persistence and in the outcome of their interaction with the host, which is also consistent with *in vitro* data. While the resident strain is superior in adhesion capacity, the presence of genes of two subtypes of tannase increases the survival potential of the probiotic strain under unfavorable conditions.

#### Bacteriocin-encoding genes

**Fig 4:**
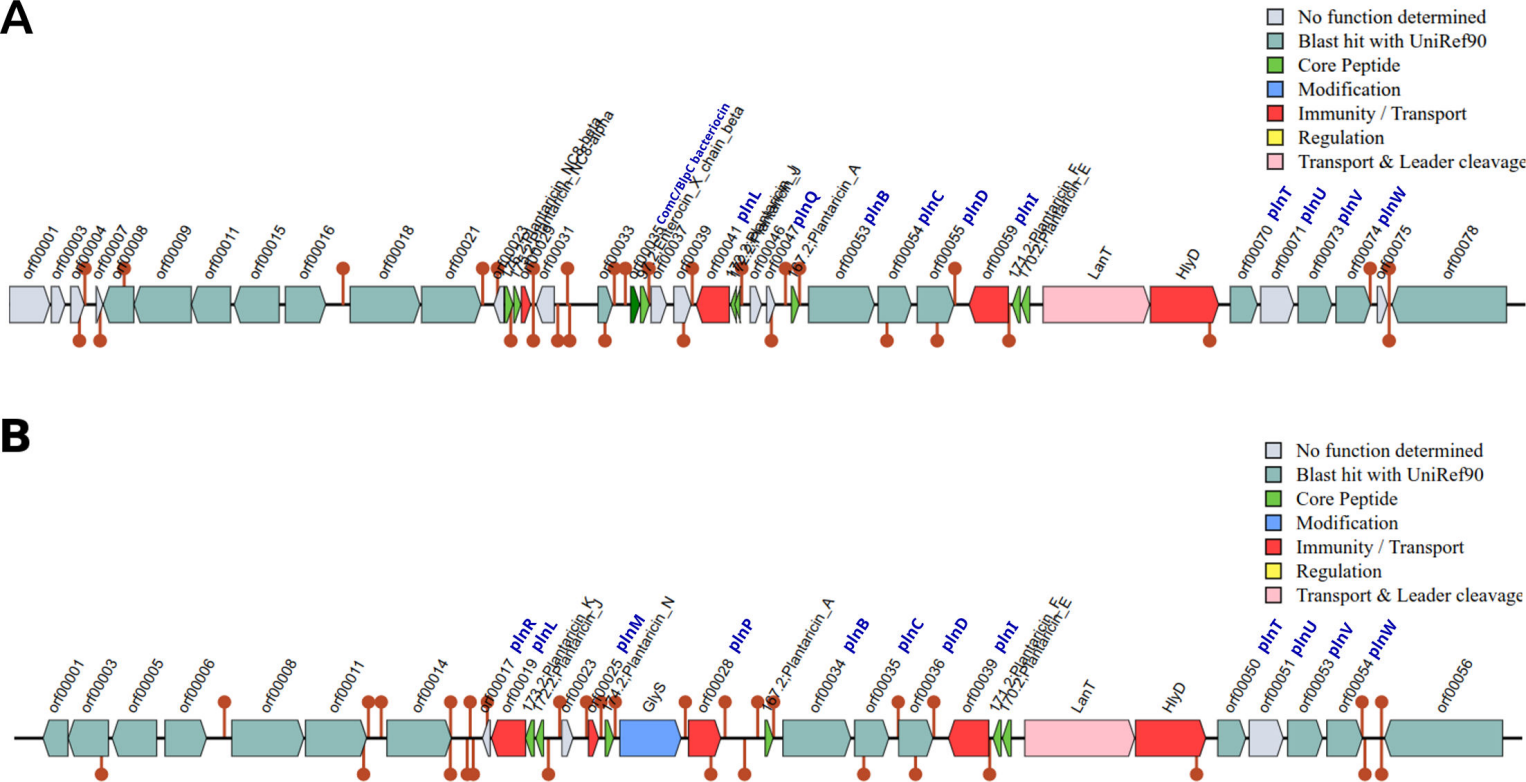
The organization of a bacteriocin synthesis cluster in the genomes of *L. plantarum* 8p-a3 (**А**) and DMC-S1 (**В**), predicted by BAGEL4. The genes in blue were determined using BLASTp.

Using the BAGEL4, clusters of bacteriocin-encoding genes have been identified in genomes of both strains (Fig. 4; Table S16).

Nevertheless, the clusters vary between strains. *L. plantarum* DMC-S1 has a cluster of genes belonging to the plantaricin J class, which contains the core peptides PlnEF, PlnA, PlnN, and PlnJK. In the *L. plantarum* 8p-a3 genome, another variant of the plantaricin cluster was found, containing genes for the core peptides PlnNC8αβ, PlnJ, PlnA, PlnEF, and Enterocin-x-β. The BLASTp analysis revealed that the genes encoding (i) bacteriocin immunity proteins PlnI and L (in both strains), PlnM and P (in DMC-S1); (ii) transcription regulators PlnC and D, histidine protein kinase PlnB, membrane proteases containing CAAX signature PlnT, U, V, and W (in both strains), protein PlnR with unknown function (in DMC-S1), protein of unknown function PlnQ, and lactococcin/core bacteriocin ComC/BlpC family leader-containing protein (in 8p-a3).

Plantaricin genes apparently provide antagonistic activity of the strains observed in *in vitro* tests against various pathogens in spot-on-lawn tests, as well as the repression of the biofilm formation by the cell-free culture liquid of these bacteria (Fig. S5 and S6). Interestingly, the antagonistic activity between these two strains was not observed in the compatibility streak-seeding test, suggesting that the strains are tolerant of the antimicrobial compounds produced (Table S16). Thus, despite some differences in the profiles of the bacteriocin cluster genes, the strains seem to be biocompatible and demonstrate similar antagonistic activity.

#### Genes-based phylogenetic reconstruction

**Fig 5:**
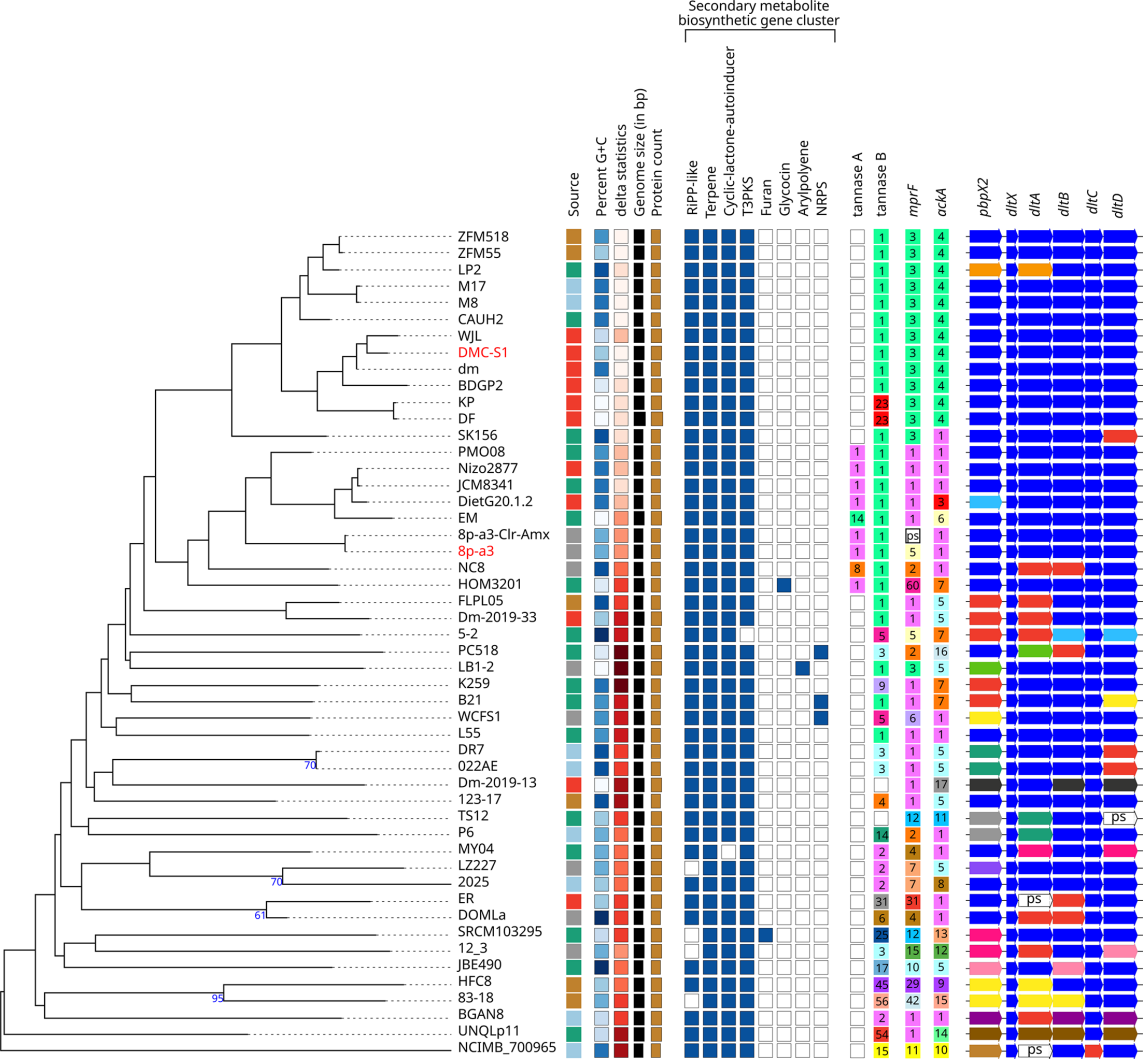
The phylogenomic tree of *L. plantarum* strains isolated from disparate sources is based on genome-wide data. The numbers adjacent to the branches are GBDP pseudo-load support values exceeding 60% in 100 replications, with an average branch support value of 14.6%. The sources of bacterial excretion are as follows: red, *D. melanogaster*; brown, human feces; green, fermented foods; blue, dairy products; and gray, other sources. The numbers in the boxes indicate different gene variants; ps, pseudogene. A phylogenomic tree was reconstructed using the GGDC web server (http://ggdc.dsmz.de/).

The analysis of average nucleotide identity (ANI), phylogenetic reconstruction based on core genes, and conserved genes encoding the crucial symbiosis factors AckA and MprF (regulating the level of immunotolerance relevant to the bacterium and the host) was used to determine the phylogenetic position of *L. plantarum* 8p-a3 and *L. plantarum* DMC-S1 among other *L. plantarum* strains isolated from sources with similar or different conditions and carrying similar or divergent gene variants (Fig. 5; Fig. S7 to S9).

*L. plantarum* DMC-S1, isolated from the intestine of *Drosophila*, has the highest homology with strains isolated from similar sources (WJL, dm, and BDGP2). The genome of *L. plantarum* 8p-a3 is more similar to other strains obtained from fermented sources, as well as the gastrointestinal tract of flies (EM, DietG20.1.2, and JCM8341). The strains closest to *L. plantarum* DMC-S1 by ANI are dm (ANI 99.95%), WJL (ANI 99.84%), and BDGP2 (ANI 99.80%). For *L. plantarum* 8p-a3 (as well as 8P-A3), the closest strains are EM (ANI 99.45%) and HOM3201 (ANI 99.31%). ANI between *L. plantarum* 8p-a3 and *L. plantarum* DMC-S1 is 98.99%. According to the phylogenetic data, a strict pattern of the relationship between the *ackA* and *mprF* gene variants or the degree of genomic homology and the sources of strains is not evident. For example, the *ackA* sequence variant, “the moderate *Drosophila*-growth-promoting ability variant,” is present in *L. plantarum* strains with varying degrees of genetic proximity that were isolated from different sources (including the commercial probiotic Lactobacterin), and vice versa (Fig. 5). Thus, the results of phylogenetic reconstruction indicate that *L. plantarum* 8p-a3 and *L. plantarum* DMC-S1 are phylogenetically close—the probiotic strain 8p-a3 turned out to be close to the *Drosophila*-associated strain line. However, the versions of the immunomodulator genes, crucial for the outcome of the interaction between *L. plantarum* and *D. melanogaster* differ between the strains.

#### Extracellular vesicles: physicochemical properties and bioactivity

An important part of the bacterial secretome, relevant for intercellular communication and targeted action on recipient cells, is associated with extracellular bacterial vesicles (3). Using TEM and NTA, we found that *L. plantarum* 8p-a3 and DMC-S1 produce extracellular vesicles (EVs), membrane-surrounded nanostructures with average diameters of 148 ± 1.9 nm and 157 ± 2 nm, respectively. These EVs represent a heterogeneous pool consisting of vesicle subpopulations that vary in size and exhibit train-specific profiles (Fig. S10A).

According to infrared spectroscopy (IR) data, lipids, nucleic acids, polysaccharides, and proteins are present in EVs of both strains (Fig. S10B). The specific features of the IR spectra in a number of frequency ranges indicate differences in the vesicular composition of the strains in terms of the qualitative and quantitative characteristics of the corresponding compounds, including proteins (1,550 cm^−1^). The LC-MS/MS proteomic analysis of EVs revealed 37 proteins in 8p-a3 vesicles and 39 proteins in DMC-S1 vesicles, among which 23 proteins were common, while 14 and 16 were strain-specific, respectively (13).

A significant part of the proteins in the vesicles of both strains belongs to those with the potential to affect the biofilms, exhibiting pro- or antibiofilm activity (Fig. S11). The spectrum of such proteins in the strains turned out to be similar, suggesting similar effects on bacterial biofilms. Indeed, *in vitro* both strains could affect the density of biofilms of some ESKAPE group pathogens. Significant decreases in the biofilms of *E. faecium*, *S. aureus*, and *P. aeruginosa* have been observed, but not *E. coli* (Fig. S12).

The *ex vivo* data indicate that vesicles of *b*oth strains can modulate immunoreactivity in the gut of *D. melanogaster* (Fig. S13), although the expression patterns of *D. melanogaster* genes associated with the immune response to vesicles of *L. plantarum* 8p-a3 and DMC-S1 differed significantly. In the case of vesicles from 8p-a3, predominant expression of genes associated with the activation of the immune response was observed, but not protection from oxidative stress (which obligately accompanies activation of immunoreactivity), whereas in the case of vesicles from DMC-S1, the opposite effect was found. A significant increase in the expression of the *Drs*, *Mtk*, *CecA1*, *PRDX5*, and *Reaper* genes recorded in the intestinal cells of flies treated with 8p-a3 vesicles (but *Drs*, *cat*, *dFoxo*, and *Reaper* in the cells treated with DMC-S1 vesicles) indicates activation (in the case of the probiotic strain) of the immune pathways associated with the induction of host tissue damage.

The *in vivo* effects of the vesicles derived from 8p-a3 and DMC-S1 proved to be in line with those of the strains. Pronounced negative effects were registered in the case of the probiotic strain (Fig. S14 and S15). Intrathoracic inoculation of adult flies with vesicles obtained from the *L. plantarum* 8p-a3 strain (but not DMC-S1) negatively affected the reproductive status of insects, reducing the number of eggs laid by females 3.5 times compared to the control group (Fig. S14A) and 3.3 times compared with flies inoculated with vesicles from DMC-S1. Inoculation of imago with vesicles from both 8p-a3 and DMC-S1 led to a significant increase in the fetal death frequency by 1.6 and 1.47 times, respectively, compared to the control group (Fig. S14B). Treatment of third-instar larvae for 24 h with vesicles isolated from the *L. plantarum* 8p-a3, but not DMC-S1, led to an increase in DNA damage in intestinal enterocytes, increasing the DNA comet index by 1.43 times compared to the control (Fig. S14E). The shift in the distribution of DNA comet types (from n0 and n1 toward n3 and n4) and histological data (Fig. S14F) also indicates the (geno)toxic effects of the probiotic strain vesicles on intestinal tissues in fruit flies.

Proteins associated with virulence, which determine the genotoxicity, are not found in vesicles of the probiotic strain, whereas the product of the *hupB* gene (HU family protein, a histone-like protein)*,* which is associated with bacterial virulence and is absent in the vesicles of the DMC-S1 strain, has been detected (Fig. S13). It can be secreted through bacterial vesicles and enter host cells, bind DNA, specifically modulate gene expression, potentially contributing to the host DNA damage and the development of pathological processes. However, so far it has been shown for pathogenic bacteria [14, 15].

Thus, these data show pronounced differences in the proteomic composition and biological effects of vesicles derived from the strains studied. The negative effects of *L. plantarum* 8p-a3 vesicles found in the experimental model used (*D. melanogaster*) determine the need for further relevant studies in higher-order model systems to confirm the safety of the probiotic strain.

## DISCUSSION

The molecular basis of the beneficial effects and the causes of the negative effects of probiotics are not entirely clear (1, 4). Clarifying these issues is important for understanding the biology of these microbes, the mechanisms of their interaction with the host and other microbes, and for assessing the safety of probiotic strains. Omics technologies have opened up new resources for obtaining relevant knowledge. New knowledge opens up new facets of probiotics and defines new directions for their research, advancing probiogenomics (9). The establishment of the fact that extracellular vesicles are key intermediaries of bacterial interaction with cells of macro- and microorganisms determines the relevance of analyzing the properties of these nanostructures for understanding the biology of intestinal commensals/symbionts, as well as for evaluating the functionality and safety of probiotic strains, especially if their producers have been found to have negative properties (3, 5).

We have previously shown that the approved probiotic strain *L. plantarum* 8p-a3 (isolated from the commercial probiotic drug Lactobacillus and used in clinical practice in Russia since 1973) exhibits negative effects on the model host organism *D. melanogaster* (Canton-S). On the contrary, the *L. plantarum* strain DMC-S1, isolated by us from the intestine of *D. melanogaster* in 2022, has an exceptionally positive effect on the host organism (12). Here, for the first time, we present the results of a comparative analysis of the functional potential and safety of these two strains to identify candidate *factors* associated with the negative effects of the probiotic strain in the *D. melanogaster* model. Through genomic analysis, extracellular vesicle studies, and *in vitro* and *in vivo* assays, we have identified the common and specific characteristics of the strains. We performed a phylogenetic reconstruction of the strains. The strains turned out to be phylogenetically similar (the probiotic turned out to be close to the “*Drosophila* lineage”), including in terms of their functional potential and safety. The strains proved to be similar in a set of genes that determine benefits to the host, as well as in the presence of some unwanted sequences (common among *L. plantarum strains* and requiring further safety studies).

Significant differences between the strains were found in the sets of genes responsible for adhesion, antimicrobial peptides, sialic acid metabolism, mucin degradation, and tannin resistance. In addition, important differences have been identified in the genes of immunomodulators, which are crucial for the microbe-host outcome, including their primary structures and expression. These genetic factors essential for the persistence of bacteria in the host can determine the specific features of strains.

The adhesion is an essential aspect for the survival of a probiotic in the gastrointestinal tract (16). Therefore, the hydrophobicity and autoaggregation are assessed as indicators of microbial potential for adhesion and attachment to the gut. The genetic potential of the strains for adhesion turned out to be different, and *in vitro* analysis indicated a correlation between genotype and phenotype. In the genomes of both strains, genes encoding proteins with LPXTG motifs that anchor the cell wall and promote bacterial colonization in the gastrointestinal tract are found (17) (Fig. S14). However, the *msa* gene, considered as a marker of reliable adhesion of probiotic *L. plantarum* strains (18), is present in DMC-S1, while it is absent in the 8p-a3 strain. This fact could explain the observed lower hydrophobicity and autoaggregation capacity of the 8p-a3 strain compared to DMC-S1 (Fig. S3 and S4). In turn, autoaggregation is believed to promote competitive exclusion of microbes from the econiche and displacement of pathogens of intestinal commensals and provides resistance to antimicrobials, survival, and persistence in certain niches (19). Interestingly, the antagonistic activities of both strains were close and relatively high against various pathogenic bacteria (Fig. S4), including the biofilms (Fig. S5 and S6). This phenomenon seems surprising against the background of significant differences between strains in relation to bacteriocin genes.

*L. plantarum* strains differ significantly in bacteriocin synthesis, and some isolates do not produce the required range of these compounds (20). Meanwhile, according to *in silico* data, *L. plantarum* 8p-a3 and DMC-S1 have significant bacteriocinogenic potential, with 29 and 31 genes involved in bacteriocin production found in the genomes of the strains, respectively (Fig. 4; Table S16). These genes may provide expression of peptides suppressing Gram-positive and Gram-negative bacteria, fungi (21), and viruses, including the *Flaviviridae* family (22, 23). These findings match the pronounced ability of these strains to suppress the growth of pathogens *in vitro* (Fig. S5 and S6). On the other hand, different patterns in the bacteriocin clusters indicate putative different capabilities of the strains to hit targets. The presence of genes for NC8αβ in the probiotic strain allows suggesting its possible contribution to the modulation of cell proliferation (24) and the structure of the host intestinal microbiota (25), also observed in the case of *L. plantarum* 8p-a3 in our studies (12). Elucidation of the effects of various plantaricins on molecular processes in host cells is a task of future research relevant to assessing the safety of probiotics.

Adhesion of probiotics to intestinal cells leads to their successful competition for binding sites with other bacteria in the community, including pathogens. Bacteria must overcome the gut mucin layer: several glycosyl hydrolases (GHs) work in cooperation in its degradation (1, 26–28). Bacteria of the genus *Lactobacillus* have a small spectrum of GHs associated with mucin degradation. According to genomic analysis, they carry genes for only 1–4 types of GHs, but not of GH33. The absence of a gene for GH33 does not allow considering the strains as involved in significant degradation of mucin. However, it should be noted that the potential of the *L. plantarum* 8p-a3 strain (GH2, GH20, GH42, and GH85) in relation to mucin-lytic abilities is still higher than that of DMC-S1 (GH2, GH20, and GH42) (Table S15).

Resistance to tannins is an essential factor for the persistence of bacteria in the intestine. The presence of tannase genes in bacteria determines the possibility of tannin degradation. Regarding the genetic determinants of tannin resistance, *L. plantarum* 8p-a3 and DMC-S1 strains differ significantly: the potential of the probiotic strain exceeds that of the DMC-S1. Genes for two tannase subtypes, A and B, were found in the genome of L. plantarum 8p-a3, while only a gene for subtype B was found in DMC-S1. Generally, *L. plantarum* strains have only the tannase B gene, and the presence of genes for two tannase subtypes is extremely rare (Fig. 5). The presence of subtype A tannase can significantly increase the potential of bacterial stress resistance, and the presence of two subtypes of tannases can cause unexpected consequences for the host. The ability of *S. gallolyticus* and *F. nucleaticus* to actively degrade tannins due to the presence of two subtypes of tannases (A and B) is considered a possible reason for the successful survival of malignant cells in the presence of these bacteria (29). It remains to be revealed whether *L. plantarum* 8p-a3, as the owner of two tannase subtypes at once, can exhibit similar properties. According to *in vitro* analysis (Fig. S1), at temperatures relevant to poikilothermic organisms, the tannase A gene is constitutively expressed in *L. plantarum* 8p-a3 cells, and at temperatures relevant to the human body, the expression level of this gene increases significantly. Whether this is the case in *vivo*, and what the consequences of this strain feature are for the host, are questions for future research relevant to assessing the safety of probiotics.

A significant contribution to bacterial persistence in higher eukaryotes is made by proteins with the potential as immunomodulators that ensure host immunotolerance against the corresponding microbes. Some mutations in such genes can dramatically affect the outcome of an interaction (symbiosis/pathogenesis). In the case of the *L. plantarum–D. melanogaster* association, such genes include *ackA* and *mprF*. The products of these genes involved in specific metabolic processes have proven to be important regulators of immunotolerance (30, 31). Mutation related to the “loss of function” in the case of *mprF* causes sensitivity to cationic peptides (12, 30), as well as a change in the LTA structure in the bacterium, and leads to disruption of immunotolerance in the host (the development of inflammatory processes in response to the persistence of this bacterium) (12, 30). Mutation related to the “loss of function” in the case of *ackA* causes an imbalance of acetyl phosphate (32), associated with the development of virulence in bacteria, as well as serious pathologies in humans (33, 34). In *L. plantarum,* this mutation was shown to be accompanied by increased formation of acetylated amino acids, including N-acetyl glutamine, which induces host immunotolerance (31). In this regard, a comparative analysis of the sequences and expression of the corresponding genes is very relevant for assessing the functionality and safety of probiotic strains. The *L. plantarum 8*p-a3 and DMC-S1 strains differ in terms of variants of primary sequences for *ackA* as well as *mprF (*Fig. S8 and S9). The sequences of these genes in the strains are “working”; their differences are associated with single non-synonymous substitutions without the effect of shifting the reading frame and the formation of stop codons. The pronounced difference in the levels of the corresponding transcripts *in L. plantarum 8p-a3* and DMC-S1 strains (Fig. S1) indicates the possibility of significant differences between the strains in terms of regulation of *ackA* and *mprF* expression. Whether that is the case remains to be seen. It is obvious that the genes of immunomodulators, which are critical for the outcome of the interaction of probiotic bacteria with the host, should be the focus of special attention in future studies. Subtle differences in the sequences and/or expression of these genes can be very significant for the metabolism of the bacterium, as well as its interaction with other microbes and host cells, and are accordingly relevant for assessing the safety of probiotics.

Pronounced differences between the strains were found in terms of composition and biological effects of their vesicles (Fig. S12 to S14). The results of in *vivo* studies indicated (geno)toxicity of the probiotic strain vesicles against intestinal tissue of the model organism used (Fig. S14), and the data correlated with *in vivo* data for the strains (12). Identifying the trigger of this effect is extremely important for understanding the molecular base of the phenomenon and evaluating the safety of probiotics.

According to the data of bioinformatic resources recommended for the search for virulence factors, proteins associated with virulence that determine the genotoxic potential are not found in probiotic vesicles of *L. plantarum* 8p-a3. Nucleases can potentially cause genotoxic effects, but the nuclease profiles of vesicles in the strains are similar, and the search tools do not identify them as the corresponding agents. We hypothesize that the DNA-binding protein of the HU family (a product of the *hupB* gene) may be a candidate factor associated with the negative effects of *L. plantarum* 8p-a3 vesicles in the *D. melanogaster model*. This protein is stably detected in vesicles of *L. plantarum* 8p-a3, but not *L. plantarum* DMC-S1. Histone-like protein HU, mediating the modulation of DNA topology and gene expression, is crucial for the survival of pathogenic bacteria (14, 35). The dual role of the HU protein (“internal” — protection of the bacterial genome and “external” — interference in the genome of host cells) makes it a key factor in persistence and virulence of pathogenic bacteria. The contribution of this protein to the interaction of commensals with host cells is not yet studied. The possibility of its participation in the negative effects of the *L. plantarum* 8p-a3 observed in the fruit fly model system remains to be investigated. Clarifying these issues is extremely important for evaluating the safety of probiotics. At the same time, taking into account the differences between the strains with respect to the infrared spectra of vesicles, it cannot be excluded that other classes of molecules may (also) be involved in the negative effects of the probiotic strain. sRNAs patterns of vesicles will require special attention (36). The features of extracellular vesicles secreted by the bacterium with GRAS status revealed through our research using the fruit fly model, open up new facets in assessing the functionality and safety of probiotics.

It is important to note that *in silico* data of our study generally correlated with in *vitro* and *in vivo data*, testifying to the validation of predictions important for evaluating the functionality and safety of probiotics. The exception was the results of the analysis of antibiotic sensitivity. The genomes of both strains contain genes *dltABCD* and *mprF*, encoding resistance to cationic antimicrobial peptides, which are crucial for survival in the gastrointestinal tract (Table S11). They belong to the intrinsic antimicrobial resistance of bacteria and are not considered to pose a significant risk in regard to the transmission of resistance to other microbes. The genes determining resistance to other groups of antibiotics are not detected in the genomes of the strains at high confidence, while some genes responsible for target protection from antibiotics, antibiotic efflux, antibiotic inactivation, and reduced permeability to antibiotics, with identity ranging from 20% to 71%, are observed (Table S11). However, *in vitro*, both strains exhibited phenotypic resistance to antibiotics of different groups (in addition to cationic antimicrobials) and differed slightly in their antibiotic sensitivity profiles (Table S12).

According to previously published reports (36–41), the phenotypic profiles of lactobacilli were determined using the standard agar disk diffusion method. Certainly, to achieve reliable results and consistency between genomic predictions and phenotypic data, it is necessary to avoid differences in experimental methods and conduct antimicrobial sensitivity testing in accordance with the recommendations of EUCAST and/or CLSI (42, 43). However, current guidelines do not establish a sufficient number of control points for testing lactobacilli for antibiotic sensitivity, probably because lactobacilli are commensals rather than clinically significant microorganisms. Future studies should adjust the relevant recommendations.

Differences in phenotypic/genotypic resistance in *Lactobacillus* isolates of various origins have also been noted in other studies (41, 44, 45). The discrepancy between genomic predictions and experimental antibiotic resistance *in L. plantarum* is biologically plausible and expected. This species has innate tolerance mechanisms such as pumps for multiple drug excretion, thickened peptidoglycan layers, and low membrane permeability, which can lead to phenotypic resistance without appropriate antibiotic resistance genes (41, 44, 46). In addition, condition-dependent gene expression, incomplete genome assembly, and database distortions can lead to underestimation or overestimation of the prediction of resistance genes. Differences in the analysis conditions (pH, seed density, and diffusion medium) can also affect the inhibition zones. Finally, the involvement of yet unknown resistance mechanisms and related genetic determinants cannot be ruled out (47–50), especially since not all genes in the genomes of even well-studied bacteria are annotated.

Despite a significant breakthrough in defining the genetic landscape *of bacteria*, part of their genome remains in the shadows (51, 52). A number of genes of *L. plantarum* 8p-a3 and *L. plantarum* DMC-S1, as well as many other probiotic strains, are genes for proteins with an unknown function (Table S16). This limitation seems to be a serious obstacle to the correct assessment of the safety of bacteria used in medicine, agriculture, food industry, and biotechnology (53). Obviously, for practical use and safety, it is advisable to use only strains with fully annotated genomes that do not contain undesirable sequences and/or to develop “precision postbiotics.”

### Study limitations

Our study has some methodological and biological limitations. In particular, the genomic data for the 8p-a3 strain is based on rough assembly, which limits the validity of SNPs in a genomic context. The analysis of the functionality and the expression of a number of critical genes was carried out only in *vitro*, without transcriptomic or proteomic/metabolomic validation, and the activity of genes of *L. plantarum* in the host intestine remains unknown. The vesicles were obtained from bacteria grown under limited environmental conditions, and the identification of their components was performed only with respect to proteins, but not metabolites, RNA, and other compounds. Finally, *in vivo* effects have been tested only in the *D. melanogaster* model. All these points need to be taken into account in further research for a deeper understanding of the interaction of the bacterium with the host, which is relevant for assessing the functionality and safety of probiotics.

### Conclusion

For the first time, a probiogenomic approach was used to identify common and specific features related to functionality and safety in phylogenetically closely related strains of the bacterium with GRAS status—the approved probiotic strain *L. plantarum* 8p-a3 and the intestinal resident *Drosophila L. plantarum* DMC-S1—which have the opposite effects on the model host organism (*D. melanogaster*). The genomic analysis of the studied strains was supplemented by the analysis of their extracellular vesicles. Integrative data analysis *in silico*, *in vitro*, and *in vivo* revealed the unique abilities of the strains associated with the potential of their beneficial and undesirable properties against the host. It was found that *L. plantarum* 8p-a3 has special properties, including those related to the effects of the bacterial vesicles *in vivo* on the fruit fly model, which determine the need for further appropriate studies in higher-order model systems to assess the safety of the probiotic strain. Emerging evidence indicates that, for assessing the safety of probiotics, not only the abundance of genes, but also variants of the primary structure of genes matter. The findings highlight the challenges associated with gaps in our knowledge of probiotic biology, as well as the standardization of probiotic strain research. It is obvious that the power of modern high-resolution methods should be directed to solving these problems in order to develop an effective system for evaluating the safety of probiotics.

## MATERIALS AND METHODS

### Bacterial strains and growth conditions

*Lactiplantibacillus plantarum* DMC-S1 has been previously isolated from the gut of *D. melanogaster* (12). *L. plantarum* 8p-a3 strain is approved as a probiotic strain (Biomed, Russia) and is effective against various dysbiosis-associated disorders (54). LAB strains were grown in MRS broth (Difco, USA), and *Staphylococcus aureus* subsp. *aureus* ATCC 29213, *Enterococcus faecium* ATCC 19434, *Escherichia coli* ATCC 25922, and *Pseudomonas aeruginosa* ATCC 27853 were used as test bacteria and grown in Luria-Bertani (LB) broth. For the biofilm assay, the BM broth (glucose 5.0 g, peptone 7.0 g, MgSO_4_·7H_2_O 2.0 g, and CaCl_2_·2H_2_O 0.05 g in 1.0 L tap water) was used (55). The LB agar plates supplemented with 5% sheep blood were used for the hemolytic activity tests. For *E. faecium*, media were supplemented with 5% fetal bovine serum (FBS).

### Bacterial antibiotic susceptibility test

Antibiotic susceptibility of the LAB strains was determined by disc diffusion assay as described in reference 50. Cultivation was carried out at 37°C for 18 h on MRS agar, as recommended in references 51–53. Bacteria were classified as resistant (R), intermediate (I), or susceptible (S), according to the EUCAST recommendations.

### Antagonistic activity

Antagonistic activity was examined by agar spot test described in (56). Briefly, overnight cultures of individual strains were spotted (2 μL) on the surface of MRS agar and incubated anaerobically (Anaerogas gaspack, NIKI MLT, Russia) for 24 h at 37°C to develop the spots. A 100 μL volume of an overnight culture of test bacteria was mixed with 7 mL of soft Luria-Bertani (LB) agar (0.7%), poured over the plate, and plates were incubated aerobically at 37°C. After 24 h of incubation, diameters of bacterial growth inhibition zones were measured. Each test was performed in triplicate.

### Cell surface hydrophobicity

The bacterial adhesion to hydrocarbons was measured as described in reference 57. The bacterial cells grown in the MRS broth at 37°C for 18 h were harvested by centrifugation, and the cell pellet was washed twice with 0.1 M KNO_3_ (pH 6.2) and resuspended in the same solution to an OD_400_ = 0.4 (A0). Bacterial cell suspension (2.4 mL) was intensively mixed with n-hexadecane (0.4 mL) and incubated at 37°C for 15 min until a complete phase separation in the mixture. The aqueous phase was gently taken out, and absorbance at 400 nm was measured (A1). The surface hydrophobicity (%) was calculated as (1 − A1/A0) × 100. Strains were classified as low (L) or medium (M) according to their hydrophobicity capacities (58).

### Autoaggregation

The autoaggregation ability of isolates was tested as described in reference 59. Briefly, the bacterial cells grown in MRS broth at 37°C for 18 h were harvested by centrifugation, washed twice with PBS, and resuspended in PBS to OD_600_ = 0.5 (A0). Bacterial cell suspensions (4 mL) were incubated at 37°C in tubes for 4 or 24 h under static conditions, and absorbance of the upper phase was measured at 600 nm (A1). Autoaggregation percentage was calculated as (1 – A1/A0) × 100.

### Isolation and characterization of the extracellular vesicles of *L. plantarum* strains

Extracellular vesicles were isolated from *L. plantarum* 8p-a3 and DMC-S1 cultures as described in reference 60 with modifications. Isolated vesicles were treated with DNase and RNase and suspended in PBS. Transmission electron microscopy (TEM) of isolated extracellular vesicles was carried out on a Hitachi HT 7800 electron microscope as described previously (61). The size distribution and concentration of extracellular vesicles in samples were determined by nanoparticle tracking analysis (NTA) using a NanoSight LM-10 analyzer (Malvern Instruments) (62). IR spectra of vesicles were recorded using an Invenio instrument (Bruker) with an MCT detector and a BioATR II ATR unit with a ZnSe crystal and a Si external coating. Spectra were recorded at a spectral resolution of 4.0 cm^–1^ with 128 scans. Band assignment was carried out as in reference 63.

### Protein identification

Vesicular proteins were identified by LC-MS using an UltiMate 3000 nanoflow HPLC instrument (Thermo, United States) as described previously (61). A C18 PepMap100 precolumn (Thermo, United States) was used with a Peaky75-30 capillary reversed-phase column (Molekta, Russia). For each spectrum, identification was performed using the MSFragger search engine of the software package FragPipe 19.1. Samples were examined by targeted mass spectrometry using a Sciex QTRAP 6500 + mass spectrometer (Sciex, United States) and an ExionLC HPLC system (Sciex, United States) to validate the proteome data. Peptides were separated on an Ultra Aqueous C18 HPLC column (3 μm, 150 × 3.0 mm) (Restek) with an Ultra Aqueous C18 HPLC guard cartridge (Restek). Identification with the MSFragger search engine of the FragPipe 19.1 package was performed for each spectrum.

### Biofilm assays

To test the antagonistic activity of *L. plantarum* strains against other bacteria in growing biofilms, *L. plantarum* and opportunistic bacteria were grown together under static conditions for 48 h in BM broth with a minimal amount of glucose (0.2%) to minimize acidification. Then, CFUs were differentially counted on Endo agar (for *E. coli*), mannitol salt agar (for *S. aureus*), and cetrimide agar (for *P. aeruginosa*) using the drop-plate assay (64).

The ability of vesicles to eradicate the formed microbial biofilm was assessed as described in reference 16. The 24-hour-old biofilms of opportunistic bacteria were washed with sterile saline (0.9% NaCl), and wells were filled with BM broth supplemented with vesicles normalized according to NTA data and incubated for the next 24 h. Then, the wells were rinsed with water, and biofilms were stained with crystal violet (64, 65). Data from five independent experiments were shown as medians with IQR.

### The effect of *L. plantarum* and vesicles on *D. melanogaster*

*D. melanogaster* Canton-S were cultured on a standard nutrient medium (agar-agar, 10.0 g; yeast, 10.0 g; semolina, 25.0 g; sugar, 25.0 g; propionic acid, 4.0 mL in 1.0 L tap water) at 25°C and a relative humidity of 65% (66). Infection of flies with *L. plantarum* 8p-a3 and *L. plantarum* DMC-S1 strains and assessment of the flies’ adaptability were performed as described earlier (12). The locomotor activity of *Drosophila* was measured using the Climbing assay; groups of 10–20 individual flies were tested in three repeats in each experiment (67).

An alkaline comet assay was performed to assess DNA damage in enterocytes of flies infected with vesicles (68). Briefly, a third-instar *D. melanogaster* larvae were infected via a nutrient substrate containing 25 µL of vesicles of *L. plantarum* 8p-a3 and DMC-S1 strains. Larvae grown in a pure medium (25 µL of PBS) served as controls. After 24-hour incubation with vesicles, intestines of larvae were extracted (*n* = 5) in Poel’s saline solution (15 mM NaCl, 6.4 mM NaH_2_PO_4_, 42 mM KCl, 7.9 mM CaCl_2_, 1.8 mM KHCO_3_, 20.8 mM MgSO_4_; pH 6.95). Further, the samples were centrifuged for 5 min (5,000 rpm) at 4°C, and a supernatant was kept and applied to 80 µL of 0.65% low-melting agarose and loaded onto glass slides. After complete solidification of the agarose, lysis was performed for 1 h at 4°C in L buffer (2.5 M NaCl, 100 mM EDTA, 10 mM Tris, 1% Triton X-100; pH 10). Next, the slides were transferred to alkaline electrophoresis buffer (0.3 M NaOH, 1 mM EDTA, pH 13) and incubated for 15 min. Electrophoresis was performed in the same buffer for 25 min (25 V, 300 mA). The slides were then neutralized in 0.4 M Tris (pH 7.5). DNA was stained with DAPI for 20 min in the dark. The slides were visualized using a Carl Zeiss Axio Imager M2 fluorescence microscope (Germany). At least 100 randomly selected cells were analyzed for each sample. The degree of DNA damage was assessed by visual classification (N0–N4).

To analyze intestinal tissue damage caused by *L. plantarum* vesicles, intestines (*n* = 5–7) were taken from infected *D. melanogaster* larvae and fixed in 4% PFA for 30 min at 25°C. Next, the samples were washed three times in PBS for 5 min and incubated twice in a PBST solution (1 × PBS with 1% Tween-20) for 15 min. Ten microliters of dye solution (propidium iodide [PI] [69], Hoechst [70], and DAPI [4′,6-diamidino-2-phenylindole] [71]) was added to samples and incubated for 5 min in the dark at 25°C. Imaging was performed on a Carl Zeiss Axio Imager M2 fluorescent microscope (Carl Zeiss, Germany) at appropriate excitation/emission wavelengths. The resulting images were processed and analyzed using ImageJ software.

### Quantitative RT-PCR

The expression of *хhlA*, *ackA*, *mprF*, *tanA,* and D-lactate dehydrogenase genes (*ldh1*, *ldh2*) in *L. plantarum* strains was evaluated by qRT-PCR using the *rpoC* as a reference gene. For that, *L. plantarum* 8p-a3 and DMC-S1 were grown in MRS broth at either 25°C or 37°C for 24 h, and total RNA was extracted from 1 mL of cultures using RNA Solo (Evrogen, Moscow, Russia).

To assess the effect of *L. plantarum* vesicles on the immune response and stress response of *D. melanogaster*, total RNA was isolated using a DNA/RNA isolation kit with a co-precipitant (Biolabmix, Novosibirsk, Russia) from intestinal homogenates (*n* = 15) of a third-instar larvae infected with vesicles as described above. The transcription of genes encoding antimicrobial peptides (Cecropin A1, Defensin, Drosocin, Drosomycin, and Metchnikowin) and stress response proteins (Catalase, dFoxo, Peroxiredoxin 5, Reaper, and ANT) was quantified by qRT-PCR using *βTub56D* and *eEF1α1* as reference housekeeping genes.

The cDNA was synthesized using MMLV RT kit (Evrogen, Moscow, Russia) with random primers following the manufacturer’s instructions. The qPCR of *хhlA*, *ackA*, *mprF*, *tanA,* and D-lactate dehydrogenase genes (*ldh1*, *ldh2*) cDNA was performed using the 5× SYBR + low ROX mix (Evrogen, Moscow, Russia) and primers presented in Table S17. The *D. melanogaster* gene expression was evaluated in qPCR performed with BioMaster HS-qPCR HI-ROX SYBR (BiolabMix, Novosibirsk, Russia). The reactions were carried out in triplicate on the QuantStudio 5 (Thermo, USA). The 2^-ΔΔCt^ method (72) was used for calculations.

### Genome sequencing

The complete nucleotide sequence of *L. plantarum* 8p-a3 genome has been determined using the MiSeq platform in 2 × 300 bp sequencing mode (Illumina, USA). The sequencing of *L. plantarum* DMC-S1 genome was performed using the NovaSeq platform in 2 × 100 bp (Illumina, USA), as well as the MinION platform (Oxford Nanopore Technologies, UK). The genome coverage was 175× for *L. plantarum* 8p-a3 and 180× (75× on the Illumina NovaSeq and 105× on the Oxford Nanopore MinION) for the *L. plantarum* DMC-S1 strain.

### Genome mining

Genome annotation was performed using the NCBI Prokaryotic Genomes Annotation Pipeline (PGAP) version 6.9 (73). The genome maps of the *L. plantarum* DMC-S1 and 8p-a3 strains were visualized using Proksee (74). Gene annotation against COG, KEGG, Pfam, and TCDB databases was performed using eggNOG mapper 2 (e-value < 0.001, identity > 40, query > 20, emapper version 2.1.12, eggNOG DB version 5.0.2) (75), and gene annotation against SEED categories was performed using RAST (76). Average nucleotide identity (ANI) analysis was performed using FastANI (77).

Comparison of the genome sequences of *L. plantarum* 8p-a3 and *L. plantarum* 8P-A3 was performed using GSAlign v.1.0.22 (78) to align the whole genomes and Bowtie 2 (79) to align the raw reads of the 8p-a3 strain to the complete genome of *L. plantarum* 8P-A3. Variant calling was performed using samtools, bcftools (80), and SNPEff (81). The variant calling threshold was set at *Q* > 30 and coverage > 15. Only variants confirmed by both whole genome and raw reads alignment methods are provided.

To search for insertion elements in the *L. plantarum* genomes, ISFinder and BLASTn v2.2.31 were used with an E-value threshold of < 5e−25 (82). The sequences encoding prophage genes were detected using the PHASTEST (83). CRISPR-Cas elements were identified using CRISPRCasFinder (84).

Clusters of genes associated with bacteriocin synthesis were identified using BAGEL4 (85) and verified with BLASTp v2.2.26 (E-value < 1e-50) (86). Antibiotic resistance genes were identified using CARD and Resistance Gene Identifier with perfect, strict, and loose hits models.

The presence of genes encoding bile acid hydrolases, esterases hydrolyzing plant phenolic compounds, and genes encoding vitamins, biogenic amines, D-lactate, and nitro compounds was determined by aligning the amino acid sequences of previously characterized genes using BLASTp v2.2.26 (E-value < 1e-50) (86).

Virulence genes were identified using the VFDB virulence factor database (87) and BLASTx (E-value < 1e-5, identity > 80%).

### Statistical analysis

Experiments were carried out in biological triplicates with three technical repeats in each, unless otherwise mentioned. Data are shown as averages with standard deviation. The statistical significance of the results was assessed using one-way analysis of variance (one-way ANOVA) with Tukey’s multiple-comparison test, with a significance threshold at *P* < 0.05, using Statistica 12.0 software. The fly survival data were analyzed using the log-rank (Mantel-Cox) test.

## Data Availability

Genome data are available in the GenBank database (BioProject IDs PRJNA528387 and PRJNA956517, and BioSample IDs SAMN11180819 and SAMN34217994 for *L. plantarum* 8p-a3 and DMC-S1, correspondingly). Raw reads were deposited to NCBI Sequence Reads Archive under SRA IDs SRR36237018 (*L. plantarum* 8p-a3, short reads), SRR36236716 (*L. plantarum* DMC-S1, long reads), and SRR36236717 (*L. plantarum* DMC-S1, short reads). Genome assembly accessions are ASM440304v2 for 8p-a3 strain and ASM2985510v1 for DMC-S1 strain.

## SUPPLEMENTAL MATERIAL

File S1 spectrum.02314-25-s0001.xlsx Islands.

Supplemental figures and tables spectrum.02314-25-s0002.pdf Fig. S1 to S15 and Tables S1 to S17.

ASM does not own the copyrights to Supplemental Material that may be linked to, or accessed through, an article. The authors have granted ASM a non-exclusive, world-wide license to publish the Supplemental Material files. Please contact the corresponding author directly for reuse.

## References

[1] da Silva TF, Glória R de A, Americo MF, Freitas ADS, de Jesus LCL, Barroso FAL, Laguna JG, Coelho-Rocha ND, Tavares LM, le Loir Y, Jan G, Guédon É, Azevedo V de C. 2024. Unlocking the potential of probiotics: a comprehensive review on research, production, and regulation of probiotics. Probiotics Antimicrob Proteins 16:1687–1723. doi:10.1007/s12602-024-10247-x 38539008

[2] Ho CS, Wong CTH, Aung TT, Lakshminarayanan R, Mehta JS, Rauz S, McNally A, Kintses B, Peacock SJ, de la Fuente-Nunez C, Hancock REW, Ting DSJ. 2025. Antimicrobial resistance: a concise update. Lancet Microbe 6:100947. doi:10.1016/j.lanmic.2024.07.010 39305919

[3] Krzyżek P, Marinacci B, Vitale I, Grande R. 2023. Extracellular vesicles of probiotics: shedding light on the biological activity and future applications. Pharmaceutics 15:522. doi:10.3390/pharmaceutics15020522 36839844 PMC9967243

[4] Merenstein D, Pot B, Leyer G, Ouwehand AC, Preidis GA, Elkins CA, Hill C, Lewis ZT, Shane AL, Zmora N, Petrova MI, Collado MC, Morelli L, Montoya GA, Szajewska H, Tancredi DJ, Sanders ME. 2023. Emerging issues in probiotic safety: 2023 perspectives. Gut Microbes 15:2185034. doi:10.1080/19490976.2023.2185034 36919522 PMC10026873

[5] Zhang X, Wang Y, E Q, Naveed M, Wang X, Liu Y, Li M. 2025. The biological activity and potential of probiotics-derived extracellular vesicles as postbiotics in modulating microbiota-host communication. J Nanobiotechnol 23:349. doi:10.1186/s12951-025-03435-6 PMC12082936 40380331

[6] Stadlbauer V. 2015. Immunosuppression and probiotics: are they effective and safe? Benef Microbes 6:823–828. doi:10.3920/BM2015.0065 26287986

[7] Liu X, Zhao H, Wong A. 2024. Accounting for the health risk of probiotics. Heliyon 10:e27908. doi:10.1016/j.heliyon.2024.e27908 38510031 PMC10950733

[8] da Silva TF, Glória R de A, de Sousa TJ, Americo MF, Freitas ADS, Viana MVC, de Jesus LCL, da Silva Prado LC, Daniel N, Ménard O, et al.. 2023. Comprehensive probiogenomics analysis of the commensal *Escherichia coli* CEC15 as a potential probiotic strain. BMC Microbiol 23:364. doi:10.1186/s12866-023-03112-4 38008714 PMC10680302

[9] Carvalho RDO, Guédon E, Aburjaile FF, Azevedo V. 2022. Editorial: probiogenomics of classic and next-generation probiotics. Front Microbiol 13:982642. doi:10.3389/fmicb.2022.982642 36060755 PMC9433118

[10] Mondala PK, Vora AA, Zhou T, Lazzari E, Ladel L, Luo X, Kim Y, Costello C, MacLeod AR, Jamieson CHM, Crews LA. 2021. Selective antisense oligonucleotide inhibition of human IRF4 prevents malignant myeloma regeneration via cell cycle disruption. Cell Stem Cell 28:623–636. doi:10.1016/j.stem.2020.12.017 33476575 PMC8026723

[11] Salikhova DI, Golovicheva VV, Fatkhudinov Tk, Shevtsova YA, Soboleva AG, Goryunov KV, Dyakonov AS, Mokroysova VO, Mingaleva NS, Shedenkova MO, Makhnach OV, Kutsev SI, Chekhonin VP, Silachev DN, Goldshtein DV. 2023. Therapeutic efficiency of proteins secreted by glial progenitor cells in a rat model of traumatic brain injury. Int J Mol Sci 24:12341. doi:10.3390/ijms241512341 37569717 PMC10419112

[12] Kostenko VV, Mouzykantov AA, Baranova NB, Boulygina EA, Markelova MI, Khusnutdinova DR, Trushin MV, Chernova OA, Chernov VM. 2022. Development of resistance to clarithromycin and amoxicillin-clavulanic acid in *Lactiplantibacillus plantarum in vitro* is followed by genomic rearrangements and evolution of virulence. Microbiol Spectr 10:e0236021. doi:10.1128/spectrum.02360-21 35579444 PMC9241834

[13] Okuda S, Watanabe Y, Moriya Y, Kawano S, Yamamoto T, Matsumoto M, Takami T, Kobayashi D, Araki N, Yoshizawa AC, Tabata T, Sugiyama N, Goto S, Ishihama Y. 2017. jPOSTrepo: an international standard data repository for proteomes. Nucleic Acids Res 45:D1107–D1111. doi:10.1093/nar/gkw1080 27899654 PMC5210561

[14] Stojkova P, Spidlova P. 2022. Bacterial nucleoid-associated protein HU as an extracellular player in host-pathogen interaction. Front Cell Infect Microbiol 12:999737. doi:10.3389/fcimb.2022.999737 36081771 PMC9445418

[15] Klimentova J, Pavkova I, Horcickova L, Bavlovic J, Kofronova O, Benada O, Stulik J. 2019*.* *Francisella tularensis* subsp. *holarctica* releases differentially loaded outer membrane vesicles under various stress conditions. Front Microbiol 10:2304. doi:10.3389/fmicb.2019.02304 31649645 PMC6795709

[16] Han S, Lu Y, Xie J, Fei Y, Zheng G, Wang Z, Liu J, Lv L, Ling Z, Berglund B, Yao M, Li L. 2021. Probiotic gastrointestinal transit and colonization after oral administration: a long journey. Front Cell Infect Microbiol 11:609722. doi:10.3389/fcimb.2021.609722 33791234 PMC8006270

[17] Ye Q, Lao L, Zhang A, Qin Y, Zong M, Pan D, Yang H, Wu Z. 2023. Multifunctional properties of the transmembrane LPxTG-motif protein derived from *Limosilactobacillus reuteri* SH-23. J Dairy Sci 106:8207–8220. doi:10.3168/jds.2023-23440 37641365

[18] Pretzer G, Snel J, Molenaar D, Wiersma A, Bron PA, Lambert J, de Vos WM, van der Meer R, Smits MA, Kleerebezem M. 2005. Biodiversity-based identification and functional characterization of the mannose-specific adhesin of *Lactobacillus plantarum*. J Bacteriol 187:6128–6136. doi:10.1128/JB.187.17.6128-6136.2005 16109954 PMC1196140

[19] Nwoko E-SQA, Okeke IN. 2021. Bacteria autoaggregation: how and why bacteria stick together. Biochem Soc Trans 49:1147–1157. doi:10.1042/BST20200718 34110370 PMC8286834

[20] Devi SM, Halami PM. 2019. Genetic variation of pln loci among probiotic *Lactobacillus plantarum* group strains with antioxidant and cholesterol-lowering ability. Probiotics Antimicrob Proteins 11:11–22. doi:10.1007/s12602-017-9336-0 29027118

[21] Syaputri Y, Iwahashi H. 2020. Characteristics of heterologous plantaricin from *Lactobacillus plantarum* and its future in food preservation. Rev Agric Sci 8:124–137. doi:10.7831/ras.8.0_124

[22] Omer AAM, Hinkula J, Tran P-T-H, Melik W, Zattarin E, Aili D, Selegård R, Bengtsson T, Khalaf H. 2022. Plantaricin NC8 αβ rapidly and efficiently inhibits flaviviruses and SARS-CoV-2 by disrupting their envelopes. PLoS One 17:e0278419. doi:10.1371/journal.pone.0278419 36449554 PMC9710782

[23] Omer AAM, Kumar S, Selegård R, Bengtsson T, Khalaf H. 2025. Characterization of novel plantaricin-derived antiviral peptides against flaviviruses. Int J Mol Sci 26:1038. doi:10.3390/ijms26031038 39940807 PMC11817140

[24] Bengtsson T, Zhang B, Selegård R, Wiman E, Aili D, Khalaf H. 2017. Dual action of bacteriocin PLNC8 αβ through inhibition of *Porphyromonas gingivalis* infection and promotion of cell proliferation. Pathog Dis 75:ftx064. doi:10.1093/femspd/ftx064 28605543 PMC5808647

[25] Pu J, Hang S, Liu M, Chen Z, Xiong J, Li Y, Wu H, Zhao X, Liu S, Gu Q, Li P. 2022. A class iib bacteriocin plantaricin NC8 modulates gut microbiota of different enterotypes *in vitro.* Front Nutr 9:877948. doi:10.3389/fnut.2022.877948 35845772 PMC9280423

[26] Bell A, Juge N. 2021. Mucosal glycan degradation of the host by the gut microbiota. Glycobiology 31:691–696. doi:10.1093/glycob/cwaa097 33043970 PMC8252862

[27] Tailford LE, Crost EH, Kavanaugh D, Juge N. 2015. Mucin glycan foraging in the human gut microbiome. Front Genet 6:81. doi:10.3389/fgene.2015.00081 25852737 PMC4365749

[28] Crouch LI, Liberato MV, Urbanowicz PA, Baslé A, Lamb CA, Stewart CJ, Cooke K, Doona M, Needham S, Brady RR, Berrington JE, Madunic K, Wuhrer M, Chater P, Pearson JP, Glowacki R, Martens EC, Zhang F, Linhardt RJ, Spencer DIR, Bolam DN. 2020. Prominent members of the human gut microbiota express endo-acting O-glycanases to initiate mucin breakdown. Nat Commun 11:4017. doi:10.1038/s41467-020-17847-5 32782292 PMC7419316

[29] Oehmcke-Hecht S, Mandl V, Naatz LT, Dühring L, Köhler J, Kreikemeyer B, Maletzki C. 2020. *Streptococcus gallolyticus* abrogates anti-carcinogenic properties of tannic acid on low-passage colorectal carcinomas. Sci Rep 10:4714. doi:10.1038/s41598-020-61458-5 32170212 PMC7070001

[30] Arias-Rojas A, Arifah AQ, Angelidou G, Alshaar B, Schombel U, Forest E, Frahm D, Brinkmann V, Paczia N, Beisel CL, Gisch N, Iatsenko I. 2024. MprF-mediated immune evasion is necessary for *Lactiplantibacillus plantarum* resilience in the *Drosophila* gut during inflammation. PLoS Pathog 20:e1012462. doi:10.1371/journal.ppat.1012462 39159259 PMC11361745

[31] Gallo M, Vento JM, Joncour P, Quagliariello A, Maritan E, Silva-Soares NF, Battistolli M, Beisel CL, Martino ME. 2022. Beneficial commensal bacteria promote *Drosophila* growth by downregulating the expression of peptidoglycan recognition proteins. iScience 25:104357. doi:10.1016/j.isci.2022.104357 35601912 PMC9121327

[32] Klein AH, Shulla A, Reimann SA, Keating DH, Wolfe AJ. 2007. The intracellular concentration of acetyl phosphate in *Escherichia coli* is sufficient for direct phosphorylation of two-component response regulators . J Bacteriol 189:5574–5581. doi:10.1128/JB.00564-07 17545286 PMC1951799

[33] Ren J, Sang Y, Qin R, Su Y, Cui Z, Mang Z, Li H, Lu S, Zhang J, Cheng S, Liu X, Li J, Lu J, Wu W, Zhao GP, Shao F, Yao YF. 2019. Metabolic intermediate acetyl phosphate modulates bacterial virulence via acetylation. Emerg Microbes Infect 8:55–69. doi:10.1080/22221751.2018.1558963 30866760 PMC6455138

[34] Beloborodova NV, Fedotcheva NI. 2024. Influence of the microbial metabolite acetyl phosphate on mitochondrial functions under conditions of exogenous acetylation and alkalization. Metabolites 14:703. doi:10.3390/metabo14120703 39728484 PMC11679681

[35] Hernández-Martínez G, Ares MA, Rosales-Reyes R, Soria-Bustos J, Yañez-Santos JA, Cedillo ML, Girón JA, Martínez-Laguna Y, Leng F, Ibarra JA, De la Cruz MA. 2024. The nucleoid protein HU positively regulates the expression of type VI secretion systems in *Enterobacter cloacae*. mSphere 9:e0006024. doi:10.1128/msphere.00060-24 38647313 PMC11324020

[36] Yu S, Zhao Z, Hao P, Qiu Y, Zhao M, Zhou G, Zhang C, Kang J, Li P. 2022. Biological functions and cross-kingdom host gene regulation of small RNAs in *Lactobacillus plantarum*-derived extracellular vesicles. Front Microbiol 13:944361. doi:10.3389/fmicb.2022.944361 36060780 PMC9436029

[37] Charteris WP, Kelly PM, Morelli L, Collins JK. 1998. Antibiotic susceptibility of potentially probiotic *Lactobacillus* species. J Food Prot 61:1636–1643. doi:10.4315/0362-028x-61.12.1636 9874341

[38] Charteris WP, Kelly PM, Morelli L, Collins JK. 2000. Effect of conjugated bile salts on antibiotic susceptibility of bile salt–tolerant *Lactobacillus* and *Bifidobacterium* Isolates. J Food Prot 63:1369–1376. doi:10.4315/0362-028X-63.10.1369 11041136

[39] Melo TA, Dos Santos TF, Pereira LR, Passos HM, Rezende RP, Romano CC. 2017. Functional profile evaluation of *Lactobacillus fermentum* TCUESC01: a new potential probiotic strain isolated during cocoa fermentation. Biomed Res Int 2017:5165916. doi:10.1155/2017/5165916 28808659 PMC5541819

[40] Haryani Y, Halid NA, Guat GS, Nor-Khaizura MAR, Hatta MAM, Sabri S, Radu S, Hasan H. 2023. High prevalence of multiple antibiotic resistance in fermented food-associated lactic acid bacteria in Malaysia. Food Control 147:109558. doi:10.1016/j.foodcont.2022.109558

[41] Duche RT, Singh A, Wandhare AG, Sangwan V, Sihag MK, Nwagu TNT, Panwar H, Ezeogu LI. 2023. Antibiotic resistance in potential probiotic lactic acid bacteria of fermented foods and human origin from Nigeria. BMC Microbiol 23:142. doi:10.1186/s12866-023-02883-0 37208603 PMC10197481

[42] The European Committee on Antimicrobial Susceptibility Testing (EUCAST). 2025. Breakpoint tables for interpretation of MICs and zone diameters. Version 15.0. Available from: https://www.eucast.org

[43] CLSI. 2024. Performance standards for antimicrobial susceptibility testing. 34th ed. CLSI Supplement M100. Clinical and Laboratory Standards Institute.

[44] Delgado S, Flórez AB, Mayo B. 2005. Antibiotic susceptibility of *Lactobacillus* and *Bifidobacterium* species from the human gastrointestinal tract. Curr Microbiol 50:202–207. doi:10.1007/s00284-004-4431-3 15902467

[45] Dušková M, Morávková M, Mrázek J, Florianová M, Vorlová L, Karpíšková R. 2020. Assessment of antibiotic resistance in starter and non-starter lactobacilli of food origin. Acta Vet Brno 89:401–411. doi:10.2754/avb202089040401

[46] Elkins CA, Mullis LB. 2004. Bile-mediated aminoglycoside sensitivity in *Lactobacillus* species likely results from increased membrane permeability attributable to cholic acid. Appl Environ Microbiol 70:7200–7209. doi:10.1128/AEM.70.12.7200-7209.2004 15574918 PMC535180

[47] Zavišić G, Popović M, Stojkov S, Medić D, Gusman V, Jovanović Lješković N, Jovanović Galović A. 2023. Antibiotic resistance and probiotics: knowledge gaps, market overview and preliminary screening. Antibiotics (Basel) 12:1281. doi:10.3390/antibiotics12081281 37627701 PMC10451169

[48] Zheng M, Zhang R, Tian X, Zhou X, Pan X, Wong A. 2017. Assessing the risk of probiotic dietary supplements in the context of antibiotic resistance. Front Microbiol 8:908. doi:10.3389/fmicb.2017.00908 28579981 PMC5437161

[49] Kerek Á, Román I, Szabó Á, Pézsa NP, Jerzsele Á. 2025. Antibiotic resistance gene expression in veterinary probiotics: two sides of the coin. Vet Sci 12:217. doi:10.3390/vetsci12030217 40266902 PMC11945515

[50] Morovic W, Roos P, Zabel B, Hidalgo-Cantabrana C, Kiefer A, Barrangou R. 2018. Transcriptional and functional analysis of *Bifidobacterium animalis* subsp. *lactis* exposure to tetracycline. Appl Environ Microbiol 84:e01999-18. doi:10.1128/AEM.01999-18 30266728 PMC6238047

[51] Price MN, Wetmore KM, Waters RJ, Callaghan M, Ray J, Liu H, Kuehl JV, Melnyk RA, Lamson JS, Suh Y, Carlson HK, Esquivel Z, Sadeeshkumar H, Chakraborty R, Zane GM, Rubin BE, Wall JD, Visel A, Bristow J, Blow MJ, Arkin AP, Deutschbauer AM. 2018. Mutant phenotypes for thousands of bacterial genes of unknown function. Nature 557:503–509. doi:10.1038/s41586-018-0124-0 29769716

[52] Almeida A, Nayfach S, Boland M, Strozzi F, Beracochea M, Shi ZJ, Pollard KS, Sakharova E, Parks DH, Hugenholtz P, Segata N, Kyrpides NC, Finn RD. 2021. A unified catalog of 204,938 reference genomes from the human gut microbiome. Nat Biotechnol 39:105–114. doi:10.1038/s41587-020-0603-3 32690973 PMC7801254

[53] Geller-McGrath D, Konwar KM, Edgcomb VP, Pachiadaki M, Roddy JW, Wheeler TJ, McDermott JE. 2024. Predicting metabolic modules in incomplete bacterial genomes with MetaPathPredict. Elife 13:e85749. doi:10.7554/eLife.85749 38696239 PMC11065424

[54] Tsapieva A, Duplik N, Suvorov A. 2011. Structure of plantaricin locus of *Lactobacillus plantarum* 8P-A3. Benef Microbes 2:255–261. doi:10.3920/BM2011.0030 22146685

[55] Kayumov AR, Khakimullina EN, Sharafutdinov IS, Trizna EY, Latypova LZ, Thi Lien H, Margulis AB, Bogachev MI, Kurbangalieva AR. 2015. Inhibition of biofilm formation in *Bacillus subtilis* by new halogenated furanones. J Antibiot (Tokyo) 68:297–301. doi:10.1038/ja.2014.143 25335695

[56] Schillinger U, Lücke FK. 1989. Antibacterial activity of *Lactobacillus* sake isolated from meat. Appl Environ Microbiol 55:1901–1906. doi:10.1128/aem.55.8.1901-1906.1989 2782870 PMC202976

[57] Rosenberg M. 2006. Microbial adhesion to hydrocarbons: twenty-five years of doing MATH. FEMS Microbiol Lett 262:129–134. doi:10.1111/j.1574-6968.2006.00291.x 16923066

[58] Ahumada MC, Colloca ME, López ME, Pesce de Ruiz Holgado A, Nader-Macias ME. 1999. Characterization of *Lactobacilli* isolated from the tongue and gum. Anaerobe 5:129–135. doi:10.1006/anae.1999.0274

[59] Kos B, Susković J, Vuković S, Simpraga M, Frece J, Matosić S. 2003. Adhesion and aggregation ability of probiotic strain *Lactobacillus acidophilus* M92. J Appl Microbiol 94:981–987. doi:10.1046/j.1365-2672.2003.01915.x 12752805

[60] Kim W, Lee EJ, Bae IH, Myoung K, Kim ST, Park PJ, Lee KH, Pham AVQ, Ko J, Oh SH, Cho EG. 2020. *Lactobacillus plantarum*‐derived extracellular vesicles induce anti‐inflammatory M2 macrophage polarization *in vitro*. J Extracell Vesicles 9:1793514. doi:10.1080/20013078.2020.1793514 32944181 PMC7480564

[61] Chernova OA, Kayumov AR, Markelova MI, Salnikov VV, Kutyreva MP, Khannanov AA, Fedorova MS, Zhuravleva DE, Baranova NB, Faizullin DA, Zuev Y, Chernov VM. 2024. Structure of extracellular vesicles and their effect on bacterial biofilms change with the development of antibiotic resistance in the probiotic strain *Lactiplantibacillus plantarum* 8p-a3. Dokl Biol Sci 519:295–300. doi:10.1134/S0012496624600246 39400899

[62] Burmatova A, Khannanov A, Gerasimov A, Ignateva K, Khaldeeva E, Gorovaia A, Kiiamov A, Evtugyn V, Kutyreva M. 2023. A hyperbranched polyol process for designing and manufacturing nontoxic cobalt nanocomposite. Polymers (Basel) 15:3248. doi:10.3390/polym15153248 37571141 PMC10421248

[63] Zucchiatti P, Mitri E, Kenig S, Billè F, Kourousias G, Bedolla DE, Vaccari L. 2016. Contribution of ribonucleic acid (RNA) to the Fourier transform infrared (ftir) spectrum of eukaryotic cells. Anal Chem 88:12090–12098. doi:10.1021/acs.analchem.6b02744 28193045

[64] Baidamshina DR, Trizna EY, Holyavka MG, Bogachev MI, Artyukhov VG, Akhatova FS, Rozhina EV, Fakhrullin RF, Kayumov AR. 2017. Targeting microbial biofilms using ficin, a nonspecific plant protease. Sci Rep 7:46068. doi:10.1038/srep46068 28387349 PMC5384253

[65] O’Toole GA, Kolter R. 1998. Initiation of biofilm formation in *Pseudomonas fluorescens* WCS365 proceeds via multiple, convergent signalling pathways: a genetic analysis . Mol Microbiol 28:449–461. doi:10.1046/j.1365-2958.1998.00797.x 9632250

[66] Gavrilova E, Kostenko V, Zadorina I, Khusnutdinova D, Yarullina D, Ezhkova A, Bogachev M, Kayumov A, Nikitina E. 2023. Repression of *Staphylococcus aureus* and *Escherichia coli* by *Lactiplantibacillus plantarum* strain AG10 in *Drosophila melanogaster in vivo* model. Microorganisms 11:1297. doi:10.3390/microorganisms11051297 37317271 PMC10223467

[67] Zhudlovsky DG, Bobkov Y, Antosyuk ON, Kostenko VV. 2025. Protective properties of *Prunella grandiflora* L. regarding the genoand cytotoxic effects of etoposide in a *Drosophila melanogaster* model with mutations in the FOXO gene. Biomedicine 21:25–29. doi:10.33647/2074-5982-21-3-25-29

[68] Mukhopadhyay I, Chowdhuri DK, Bajpayee M, Dhawan A. 2004. Evaluation of *in vivo* genotoxicity of cypermethrin in *Drosophila melanogaster* using the alkaline comet assay. Mutagenesis 19:85–90. doi:10.1093/mutage/geh007 14981154

[69] Singh SR, Zeng X, Zhao J, Liu Y, Hou G, Liu H, Hou SX. 2016. The lipolysis pathway sustains normal and transformed stem cells in adult *Drosophila*. Nature 538:109–113. doi:10.1038/nature19788 27680705 PMC7798135

[70] Priyadarsini S, Sahoo M, Sahu S, Jayabalan R, Mishra M. 2019. An infection of *Enterobacter ludwigii* affects development and causes age-dependent neurodegeneration in *Drosophila melanogaster*. Invert Neurosci 19:13. doi:10.1007/s10158-019-0233-y 31641932

[71] Priyadarsini S, Mukherjee S, Mishra M. 2020. Methodology to detect the abnormality of *Drosophila* gut by various staining techniques, p 51–64. *In* Mishra M (ed), Fundamental approaches to screen abnormalities in Drosophila. Springer, New York, NY.

[72] Livak KJ, Schmittgen TD. 2001. Analysis of relative gene expression data using real-time quantitative PCR and the 2^−ΔΔ^*^C^*^_T_^ Method. Methods 25:402–408. doi:10.1006/meth.2001.1262 11846609

[73] Tatusova T, DiCuccio M, Badretdin A, Chetvernin V, Nawrocki EP, Zaslavsky L, Lomsadze A, Pruitt KD, Borodovsky M, Ostell J. 2016. NCBI prokaryotic genome annotation pipeline. Nucleic Acids Res 44:6614–6624. doi:10.1093/nar/gkw569 27342282 PMC5001611

[74] Grant JR, Enns E, Marinier E, Mandal A, Herman EK, Chen C, Graham M, Van Domselaar G, Stothard P. 2023. Proksee: in-depth characterization and visualization of bacterial genomes. Nucleic Acids Res 51:W484–W492. doi:10.1093/nar/gkad326 37140037 PMC10320063

[75] Cantalapiedra CP, Hernández-Plaza A, Letunic I, Bork P, Huerta-Cepas J. 2021. eggNOG-mapper v2: functional annotation, orthology assignments, and domain prediction at the metagenomic scale. Mol Biol Evol 38:5825–5829. doi:10.1093/molbev/msab293 34597405 PMC8662613

[76] Overbeek R, Olson R, Pusch GD, Olsen GJ, Davis JJ, Disz T, Edwards RA, Gerdes S, Parrello B, Shukla M, Vonstein V, Wattam AR, Xia F, Stevens R. 2014. The seed and the rapid annotation of microbial genomes using subsystems technology (RAST). Nucl Acids Res 42:D206–D214. doi:10.1093/nar/gkt1226 24293654 PMC3965101

[77] Jain C, Rodriguez-R LM, Phillippy AM, Konstantinidis KT, Aluru S. 2018. High throughput ANI analysis of 90K prokaryotic genomes reveals clear species boundaries. Nat Commun 9:5114. doi:10.1038/s41467-018-07641-9 30504855 PMC6269478

[78] Lin HN, Hsu WL. 2020. GSAlign: an efficient sequence alignment tool for intra-species genomes. BMC Genomics 21:182. doi:10.1186/s12864-020-6569-1 32093618 PMC7041101

[79] Langmead B, Salzberg SL. 2012. Fast gapped-read alignment with Bowtie 2. Nat Methods 9:357–359. doi:10.1038/nmeth.1923 22388286 PMC3322381

[80] Danecek P, Bonfield JK, Liddle J, Marshall J, Ohan V, Pollard MO, Whitwham A, Keane T, McCarthy SA, Davies RM, Li H. 2021. Twelve years of SAMtools and BCFtools. Gigascience 10:giab008. doi:10.1093/gigascience/giab008 33590861 PMC7931819

[81] Cingolani P. 2022. Variant annotation and functional prediction: SnpEff. Methods Mol Biol 2493:289–314. doi:10.1007/978-1-0716-2293-3_19 35751823

[82] Siguier P, Perochon J, Lestrade L, Mahillon J, Chandler M. 2006. ISfinder: the reference centre for bacterial insertion sequences. Nucleic Acids Res 34:D32–D36. doi:10.1093/nar/gkj014 16381877 PMC1347377

[83] Wishart DS, Han S, Saha S, Oler E, Peters H, Grant JR, Stothard P, Gautam V. 2023. PHASTEST: faster than PHASTER, better than PHAST. Nucleic Acids Res 51:W443–W450. doi:10.1093/nar/gkad382 37194694 PMC10320120

[84] Couvin D, Bernheim A, Toffano-Nioche C, Touchon M, Michalik J, Néron B, Rocha EPC, Vergnaud G, Gautheret D, Pourcel C. 2018. CRISPRCasFinder, an update of CRISRFinder, includes a portable version, enhanced performance and integrates search for cas proteins. Nucleic Acids Res 46:W246–W251. doi:10.1093/nar/gky425 29790974 PMC6030898

[85] van Heel AJ, de Jong A, Song C, Viel JH, Kok J, Kuipers OP. 2018. BAGEL4: a user-friendly web server to thoroughly mine RiPPs and bacteriocins. Nucleic Acids Res 46:W278–W281. doi:10.1093/nar/gky383 29788290 PMC6030817

[86] Surve S, Shinde DB, Kulkarni R. 2022. Isolation, characterization and comparative genomics of potentially probiotic *Lactiplantibacillus plantarum* strains from Indian foods. Sci Rep 12:1940. doi:10.1038/s41598-022-05850-3 35121802 PMC8816928

[87] Liu B, Zheng D, Jin Q, Chen L, Yang J. 2019. VFDB 2019: a comparative pathogenomic platform with an interactive web interface. Nucleic Acids Res 47:D687–D692. doi:10.1093/nar/gky1080 30395255 PMC6324032

