# Supplementary material for: Probiogenomic analysis of functional potential and safety of L. plantarum 8p-a3 and DMC-S1 strains: in silico vs in vitro and in vivo data
Source: Microbiol Spectr. 2026 Jul 6;14(8):e02314-25. doi: 10.1128/spectrum.02314-25 (PMC13435645; doi:10.1128/spectrum.02314-25)
Supplement: Supplementary file 1 [file spectrum.02314-25-s0002.pdf]

Probiogenomic analysis of functional potential and safety of *L. plantarum* 8p-a3 and DMC-S1 strains: *in silico* vs. *in vitro* and *in vivo* data
Supplementry data

Maria Markelova,[a,b,#] Victoria Kostenko,[a] Arina Aristova,[b] Maria Nikolaeva,[a] Maya Kharitonova,[a,b] Shamil Validov,[c] Airat Kayumov,[a] Olga Chernova,[b] Vladislav Chernov[b]

Table S1. Clusters of Orthologous Genes (COG) one letter code descriptions

Category' group	One letter code of category	Description of category
Information storage and processing	J	Translation, ribosomal structure and biogenesis
	A	RNA processing and modification
	K	Transcription
	L	Replication, recombination and repair
	B	Chromatin structure and dynamics
Cellular processes and signaling	D	Cell cycle control, cell division, chromosome partitioning
	Y	Nuclear structure
	V	Defense mechanisms
	T	Signal transduction mechanisms
	M	Cell wall/membrane/envelope biogenesis
	N	Cell motility
	Z	Cytoskeleton
	W	Extracellular structures
	U	Intracellular trafficking, secretion, and vesicular transport
	O	Posttranslational modification, protein turnover, chaperones
Metabolism	C	Energy production and conversion
	G	Carbohydrate transport and metabolism
	E	Amino acid transport and metabolism
	F	Nucleotide transport and metabolism
	H	Coenzyme transport and metabolism
	I	Lipid transport and metabolism
	P	Inorganic ion transport and metabolism
	Q	Secondary metabolites biosynthesis, transport and catabolism
Poorly characterized	R	General function prediction only
	S	Function unknown

Table S2. KEGG orthology (KO) categories of identified protein-coding genes in genomes of *L. plantarum* 8p-a3 and DMC-S1

**Functional Category**	**Gene number (%)**	
	**8p-a3**	**DMC-S1**
Carbohydrate metabolism	206 (13.49%)	222 (14.16%)
Energy metabolism	65 (4.26%)	64 (4.08%)
Lipid metabolism	42 (2.75%)	42 (2.68%)
Nucleotide metabolism	60 (3.93%)	59 (3.76%)
Amino acid metabolism	138 (9.04%)	138 (8.80%)
Metabolism of other amino acids	35 (2.29%)	35 (2.23%)
Glycan biosynthesis and metabolism	47 (3.08%)	49 (3.13%)
Metabolism of cofactors and vitamins	73 (4.78%)	73 (4.66%)
Metabolism of terpenoids and polyketides	17 (1.11%)	17 (1.08%)
Biosynthesis of other secondary metabolites	27 (1.77%)	26 (1.66%)
Xenobiotics biodegradation and metabolism	22 (1.44%)	22 (1.40%)
Genetic Information Processing	178 (11.66%)	179 (11.42%)
Environmental Information Processing	169 (11.07%)	178 (11.35%)
Cellular Processes	64 (4.19%)	64 (4.08%)
Organismal Systems	37 (2.42%)	37 (2.36%)
Human Diseases	79 (5.17%)	79 (5.04%)

Table S3. SEED subsystem categories of identified protein-coding genes in genomes of *L. plantarum* 8p-a3 and DMC-S1

**SEED category**	**Gene number (%)**	
	**8p-a3**	**DMC-S1**
Carbohydrates	193 (20.8%)	197 (20.3%)
Amino Acids and Derivatives	158 (17.0%)	162 (16.7%)
Protein Metabolism	126 (13.5%)	133 (13.7%)
Cofactors, Vitamins, Prosthetic Groups, Pigments	86 (9.2%)	84 (8.7%)
Nucleosides and Nucleotides	84 (9.0%)	85 (8.8%)
DNA Metabolism	52 (5.6%)	51 (5.3%)
RNA Metabolism	39 (4.2%)	34 (3.5%)
Virulence, Disease and Defense	37 (4.0%)	35 (3.6%)
Membrane Transport	22 (2.4%)	22 (2.3%)
Stress Response	19 (2.0%)	20 (2.1%)
Cell Wall and Capsule	17 (1.8%)	30 (3.1%)
Fatty Acids, Lipids, and Isoprenoids	16 (1.7%)	18 (1.9%)
Respiration	14 (1.5%)	14 (1.4%)
Miscellaneous	13 (1.4%)	14 (1.4%)
Regulation and Cell signaling	12 (1.3%)	16 (1.6%)
Phages, Prophages, Transposable elements, Plasmids	10 (1.1%)	22 (2.3%)
Phosphorus Metabolism	6 (0.6%)	6 (0.6%)
Iron acquisition and metabolism	5 (0.5%)	5 (0.5%)
Potassium metabolism	5 (0.5%)	6 (0.6%)
Cell Division and Cell Cycle	4 (0.4%)	4 (0.4%)
Metabolism of Aromatic Compounds	4 (0.4%)	4 (0.4%)
Secondary Metabolism	4 (0.4%)	4 (0.4%)
Sulfur Metabolism	3 (0.3%)	3 (0.3%)
Dormancy and Sporulation	1 (0.1%)	1 (0.1%)

Table S4. IS elements found in *L. plantarum* DMC-S1 и 8p-a3

Sequence	IS Family	Region	Location	Origin	Score (bits)	E. value	Identity (%)
8p-a3							
ISP2	IS1182	NZ_SOQA02000010.1:105324-105790	Chromosome	*L. plantarum*	918	0.0	99
DMC-S1							
ISP2	IS1182	NZ_CP123053.1:2208470-2210265	Chromosome	*L. plantarum*	3441	0.0	99
ISP1	ISL3	NZ_CP123053.1:3060326-3062121	Chromosome	*L. plantarum*	2722	0.0	99
IS1310	IS256	NZ_CP123054.1: 7376-8551	Plasmid A	*Enterococcus hirae*	2196	0.0	95
ISLsa1	IS30	NZ_CP123055.1: 14648-15424	Plasmid B	*Lactobacillus sakei*	371	6e-101	87

By using a BLASTN and an ISFinder search, a single-copy IS1182 (ISP2) was detected in the genomes of both strains (identity 99%). One copy of ISL3 is present in the genome of *L. plantarum* DMC-S1 (identity 99%). In addition, IS256 and IS30 (identity 95% and 87%) were detected on the plasmids of *L. plantarum* DMC-S1 strain. Plasmid of *L. plantarum* 8P-A3 also contains IS - 1 ISLpl1 from the IS30 family (identity 98%).

Table S5. Prophage regions of *L. plantarum* 8p-a3 and DMC-S1 identified in PHASTEST.

Strain	Region	Length (kb)	Completeness	Score	Total Proteins	Region Position (bp)	Most Common Phage (Number of Matching Proteins)	GC%	attL/ attR Sites	Integrase
8p-a3	1	48.9	intact	150	59	NZ_SOQA02000009.1:61360-110334	PHAGE_Lactob_Sha1_NC_019489(39)	41.43%	+	+
8p-a3	2	32.6	intact	150	42	NZ_SOQA02000001.1:3-32606	PHAGE_Lactob_phig1e_NC_004305(6)	41.66%	-	-
8p-a3	3	39	intact	130	47	NZ_SOQA02000011.1:64690-103737	PHAGE_Oenoco_phiS13_NC_023560(15)	42.07%	-	-
8p-a3	4	16.7	questionable	70	29	NZ_SOQA02000007.1:122647-139419	PHAGE_Strept_315.2_NC_004585(3)	42.12%	+	+
DMC-S1	1	45.2	intact	150	63	NZ_CP123053.1:921253-966515	PHAGE_Lactob_phig1e_NC_004305(8)	42.31%	+	+
DMC-S1	2	44.3	intact	150	56	NZ_CP123053.1:1044103-1088430	PHAGE_Lactob_Sha1_NC_019489(32)	41.69%	+	+
DMC-S1	3	42.6	intact	150	60	NZ_CP123053.1:1278181-1320837	PHAGE_Lactob_Sha1_NC_019489(26)	41.50%	+	+
DMC-S1	4	52.8	intact	140	65	NZ_CP123053.1:2211371-2264198	PHAGE_Oenoco_phi9805_NC_023559(15)	41.42%	+	+
DMC-S1	5	53.9	intact	150	63	NZ_CP123053.1:2270714-2324642	PHAGE_Lactob_phijl1_NC_006936(16)	41.51%	+	+
DMC-S1	6	17.6	questionable	70	24	NZ_CP123053.1:36034-53690	PHAGE_Lactob_Sha1_NC_019489(2)	41.23%	+	+

The PHASTER analysis revealed five complete and one partial prophage in *L. plantarum* DMC-S1 genome, and three complete and one partial prophage in *L. plantarum* 8p-a3 genome. Each prophage region mainly consists of genes for bacteriophage proteins from various genera, including *Lactobacillus*, *Oenococcus*, and *Streptococcus*. In the genome of 8p-a3 strain, one of the complete prophages is similar to PHAGE_Lactob_Sha1_NC_019489; in the genome of *L. plantarum* DMC-S1, two complete and one incomplete prophages had the highest similarity to this phage. The prophage close to PHAGE_Lactob_phig1e_NC_004305 was identified in both genomes.

Table S6. List of genes found in plasmid A of *L. plantarum* DMC-S1

#	Start	End	Product	Pseudogene	COG Category
1	678	349	DUF202 domain-containing protein	-	
2	1561	716	PTS system mannose/fructose/sorbose family transporter subunit IID	+	[G] Carbohydrate transport and metabolism
3	2412	1564	PTS sugar transporter subunit IIC	-	[M] Cell wall/membrane/envelope biogenesis
4	2940	2446	PTS sugar transporter subunit IIB	-	[G] Carbohydrate transport and metabolism
5	3390	2956	PTS fructose transporter subunit IIA	-	[G] Carbohydrate transport and metabolism
6	3615	6152	PRD domain-containing protein	-	[K] Transcription
7	7174	6620	**recombinase family protein**	-	[L] Replication, recombination and repair
8	8551	7376	**IS256 family transposase**	-	[L] Replication, recombination and repair
9	11156	9477	alpha-glucosidase	-	[G] Carbohydrate transport and metabolism
10	11993	11166	PTS system mannose/fructose/sorbose family transporter subunit IID	-	[G] Carbohydrate transport and metabolism
11	12844	12002	PTS sugar transporter subunit IIC	-	[G] Carbohydrate transport and metabolism
12	13348	12863	PTS sugar transporter subunit IIB	-	[G] Carbohydrate transport and metabolism
13	13780	13361	PTS fructose IIA subunit	-	[G] Carbohydrate transport and metabolism
14	16510	13919	sigma 54-interacting transcriptional regulator	-	[K] Transcription
15	17437	16898	PadR family transcriptional regulator	-	[K] Transcription
16	19050	19334	hypothetical protein	+	[E] Amino acid transport and metabolism
17	19327	20070	APC family permease	+	[E] Amino acid transport and metabolism
18	20084	20869	cyclase family protein	-	[S] Function unknown
19	22421	21273	FRG domain-containing protein	-	
20	22681	22758	putative holin-like toxin	-	
21	23038	24438	MFS transporter	-	[E] Amino acid transport and metabolism
22	24438	25100	HD domain-containing protein	-	[S] Function unknown
23	25090	26244	S-adenosyl-L-homocysteine hydrolase	-	[H] Coenzyme transport and metabolism
24	27851	26625	**IS110 family transposase**	-	[L] Replication, recombination and repair
25	28226	28288	putative holin-like toxin	-	
26	29358	28445	hypothetical protein	+	
27	30061	29642	MarR family transcriptional regulator	-	[K] Transcription
28	33925	30074	bacterial Ig-like domain-containing protein	-	[M] Cell wall/membrane/envelope biogenesis
29	35572	34847	antiholin-like protein LrgB	-	[M] Cell wall/membrane/envelope biogenesis
30	36044	35574	CidA/LrgA family protein	-	[S] Function unknown

31	36896	36180	LytTR family transcriptional regulator DNA-binding domain-containing protein	-	[K] Transcription
32	38644	36926	sensor histidine kinase	-	[T] Signal transduction mechanisms
33	39431	39004	MarR family transcriptional regulator	+	[K] Transcription
34	40314	39544	acetoin reductase	-	[I] Lipid transport and metabolism
35	40816	40343	S-ribosylhomocysteine lyase	-	[H] Coenzyme transport and metabolism
36	41726	40866	MetQ/NlpA family ABC transporter substrate-binding protein	-	[P] Inorganic ion transport and metabolism
37	42455	41778	ABC transporter permease	-	[P] Inorganic ion transport and metabolism
38	43531	42455	ATP-binding cassette domain-containing protein	-	[P] Inorganic ion transport and metabolism
39	44011	43532	GAF domain-containing protein	-	[T] Signal transduction mechanisms
40	44988	45253	amino acid permease	+	
41	45517	46914	amino acid permease	-	[E] Amino acid transport and metabolism
42	47672	47217	**transposase**	-	[L] Replication, recombination and repair
43	50981	50784	hypothetical protein	-	
44	51670	51350	hypothetical protein	+	
45	52263	51683	**site-specific integrase**	+	[L] Replication, recombination and repair
46	52342	52620	type II toxin-antitoxin system prevent-host-death family antitoxin	-	[D] Cell cycle control, cell division, chromosome partitioning
47	52620	52976	type II toxin-antitoxin system YafQ family toxin	-	[S] Function unknown
48	53105	53737	**DDE-type integrase/transposase/recombinase**	-	[L] Replication, recombination and repair
49	54520	54236	helix-turn-helix transcriptional regulator	-	[K] Transcription
50	54867	54523	type II toxin-antitoxin system RelE/ParE family toxin	-	[S] Function unknown
51	55005	55585	**site-specific integrase**	+	[L] Replication, recombination and repair
52	55941	56149	hypothetical protein	+	
53	57566	57234	hypothetical protein	-	
54	58596	59426	AAA family ATPase	-	[D] Cell cycle control, cell division, chromosome partitioning
55	59419	59727	hypothetical protein	-	
56	59799	59924	hypothetical protein	-	
57	61467	61712	hypothetical protein	-	[L] Replication, recombination and repair
58	62407	62868	hypothetical protein	+	[L] Replication, recombination and repair

In plasmids of *L. plantarum* DMC-S1, genes associated with the virulence factor or toxic metabolites, as well as AMR genes, were not detected. However, factors potentially leading to genomic rearrangements (genes encoding for recombinases, transposases, and integrases) were found in plasmid A (highlighted in bold).

Table S7. List of genes found in plasmid B of *L. plantarum* DMC-S1

#	Start	End	Product	Pseudogene	COG Category
1	594	1	hypothetical protein	-	
2	813	598	hypothetical protein	-	
3	2446	935	DNA topoisomerase	-	[L] Replication, recombination and repair
4	4564	2501	MobQ family relaxase	-	[L] Replication, recombination and repair
5	4834	5046	hypothetical protein	-	
6	5063	5341	hypothetical protein	-	
7	6223	5468	Fic family protein	-	[S] Function unknown
8	6466	6269	hypothetical protein	-	
9	7708	6611	replication initiator protein A	-	[S] Function unknown
10	8530	8327	hypothetical protein	-	
11	9361	8576	ParA family protein	-	[D] Cell cycle control, cell division, chromosome partitioning
12	10222	9647	TetR/AcrR family transcriptional regulator	-	[K] Transcription
13	10387	12231	MFS transporter	-	[E] Amino acid transport and metabolism
14	12895	12326	**recombinase family protein**	-	[L] Replication, recombination and repair
15	13719	13159	TetR family transcriptional regulator	-	
16	13809	14303	HXXEE domain-containing protein	-	[S] Function unknown
17	14648	15424	**IS30 family transposase**	+	[L] Replication, recombination and repair
18	15892	15467	hypothetical protein	-	
19	16551	15994	Abi family protein	-	
20	18889	17225	ATP-binding protein	-	[S] Function unknown
21	20063	18879	SIR2 family protein	-	[S] Function unknown
22	23545	20105	phospholipase D-like domain-containing protein	-	[L] Replication, recombination and repair
23	27017	23538	hypothetical protein	-	[V] Defense mechanisms
24	28841	27465	HlyD family secretion protein	-	[M] Cell wall/membrane/envelope biogenesis
25	31007	28857	peptide cleavage/export ABC transporter	-	[V] Defense mechanisms
26	31763	31258	**recombinase family protein**	+	[L] Replication, recombination and repair
27	32746	32210	hypothetical protein	-	
28	33528	33391	hypothetical protein	-	

In the plasmids of *L. plantarum* DMC-S1, genes associated with the virulence factor or toxic metabolites, as well as AMR genes, were not detected. However, factors potentially leading to genomic rearrangements (genes encoding for recombinases, transposases, and integrases) were found in plasmid B (**highlighted in bold**).

Table S8. Genes located in *L. plantarum* 8P-A3 plasmid

#	Start	End	Product	Pseudogene	COG Category
1	1; 9316	423; 9480	**Tyrosine-type recombinase/integrase**	-	[L] Replication, recombination and repair
2	2641	3144	DUF536 domain-containing protein	-	
3	3370	3459	**IS30 family transposase**	+	[L] Replication, recombination and repair
4	3501	3968	DNA starvation/stationary phase protection protein	-	[P] Inorganic ion transport and metabolism
5	5712	5976	**IS5 family transposase**	frameshift, ribosomal slippage	[L] Replication, recombination and repair
6	6035	8302	DUF1906 domain-containing protein	+	
7	8627	8974	type II toxin-antitoxin system PemK/MazF family toxin	-	[T] Signal transduction mechanisms
8	8974	9237	AbrB/MazE/SpoVT family DNA-binding domain-containing protein	-	[S] Function unknown

Plasmid of *L. plantarum* 8P-A3 also contains recombinase/integrase and transposase genes, while the latter are identified as pseudogenes (**highlighted in bold**).

Table S9. Crispr array system (a) and Cas type detection (b) within genomes of *L. plantarum* 8p-a3 and DMC-S1 using CRISPRCasFinder tool

(a)

Strain	CRISPR_ID	Start	End	Length (bp)	Orientation	Consensus Repeat	No. of CRISPRs with Same Repeat (Crisprdb)	Repeat Length	No. of Spacers	Evidence Level
8p-a3	NZ_SOQA02000003_1	206278	206363	85	ND	TAAGAAACTTAAAGTGTCTTATT	0	23	1	1
8p-a3	NZ_SOQA02000018_1	26163	26360	198	ND	GGCCGACGTTGCACTCTGGCTCGTCGTGCTAGTGACTGAACTAGCACT	0	48	1	1
DMC-S1	NZ_CP123053_1	2852793	2852878	85	ND	TAAGAAACTTAAAGTGTCTTATT	0	23	1	1

(b)

Strain	Sequence ID	Cas-Type/Subtype	System	Type	Start	End	Strand
8p-a3	NZ_SOQA02000015_1	cas3_TypeI	CAS	CDS	44029	45372	+

Table S10. Genes of enzymes involved in synthesis of biogenic amines, D-lactate, nitrocompounds found in *L. plantarum* DMC-S1 и 8p-a3

Category	Protein ID/accession number	Product	Source Organism	% identity	
				8p-a3	DMC-S1
Biogenic amines	DQ132891	Histidine decarboxylase	*Ligilactobacillus saerimneri* 30a	0	0
	AF354231	Tyrosine decarboxylase operon	*Enterococcus faecalis*	0	0
	AFC60624.1	Ornithine decarboxylase	*Levilactobacillus brevis*	0	0
	AFC60625.1	Ornithine/putrescine exchanger		0	0
	AGG35759.1	Putrescine carbamoyltransferase, partial		0	0
	EKW98931.1	Ornithine decarboxylase	*Ligilactobacillus saerimneri* 30a	0	0
D-lactate	Q88VJ2	D-lactate dehydrogenase	*L. plantarum* WCFS1	100	100
Nitro compounds	Q890E7	FMN-dependent NADH:quinone oxidoreductase 1	*L. plantarum* WCFS1	99.07	99.07
	Q88Y41	FMN-dependent NADH:quinone oxidoreductase 2	*L. plantarum* WCFS1	98.56	98.56

Table S11. AMR (antimicrobial resistance) genes identified in genomes of *L. plantarum* 8p-a3 and DMC-S1

**Drug Class**	**Resistance Mechanism**	**Best hit gene**	**% identity of hit obtained in**	
			**8p-a3**	**DMC-S1**
Aminocoumarin antibiotic	Antibiotic efflux	novA	32.81	32.81
Aminoglycoside antibiotic	Antibiotic efflux	kdpE	0	31.72
		RanA	35.71	34.11
		baeS	28.06	27.76
	Antibiotic inactivation	AAC(6')-Ian	33.33	33.33
		ANT(3")-IIc	28.64	27.47
Carbapenem; cephalosporin; penam	Antibiotic inactivation	NmcR	35	35
		GOB-24	0	26.32
		PNGM-1	0	23.67
Cephalosporin	Antibiotic inactivation	CMA-2	28.06	28.06
		EC-18	25.17	25.17
		PSZ-1	23.41	23.41
	Reduced permeability to antibiotic	IreK	46.52	46.35
Cephamycin	Antibiotic inactivation	CMY-128	26.35	26.35
Diaminopyrimidine antibiotic	Antibiotic target replacement	dfrE	41.57	42.17
Disinfecting agents and antiseptics	Antibiotic efflux	emeA	40.2	0
		qacE	36.11	36.11
		qacH	40.59	40.59
		qacJ	31.37	31.37
Elfamycin antibiotic	Antibiotic efflux	facT	28.35	28.35
Fluoroquinolone antibiotic	Antibiotic efflux	emrR	26.4	26.4
		mdtH	21.59	21.59
		norB	30.46	30.46
		patA	49.82	49.82
		patB	35.07	35.07
		QepA2	31.32	0
		QepA4	25.38	25
		Rv1877	27.83	27.83
		norA	24.08	24.08
		YajC	30.38	30.38
		mgrA	41.75	41.75
		MexS	41.33	41.33
		arlR	55.95	55.95
		arlS	0	32.06
		cdeA	20.15	20.15
		marA	21.57	21.57
		bmr	28.16	39.11
		adeL	30.33	30.33
	Antibiotic target protection	*QnrVC3*	27.42	26.61
Fusidane antibiotic	Antibiotic target protection	*FusF*	42.08	42.08

Glycopeptide antibiotic	Antibiotic target alteration	D-Ala-D-Ala ligase	36.86	36.86
		vanH gene in *vanA* cluster	35.02	35.02
		vanH gene in *vanD* cluster	39.33	39.33
		vanH gene in *vanF* cluster	27.69	27.69
		vanH gene in *vanM* cluster	26.23	26.23
		vanH gene in *vanP* cluster	32.39	32.7
		vanR gene in *vanF* cluster	51.11	51.11
		vanR gene in *vanM* cluster	47.32	47.32
		vanS gene in *vanF* cluster	32.24	32.24
		vanT gene in *vanG* cluster	31.44	31.44
		vanX gene in *vanP* cluster	32.35	29.83
		vanY gene in *vanB* cluster	31.93	31.93
		vanZ gene in *vanF* cluster	38.1	38.1
Glycylcycline; tetracycline antibiotic	Antibiotic efflux	*mepA*	30.18	29.95
		mepR	26.12	26.12
Lincosamide antibiotic	Antibiotic efflux	*lmrD*	56.18	56.18
		lmrB	45.11	45.11
	Antibiotic inactivation	*lin*	31.98	32.19
	Antibiotic target protection	*lmrC*	35.35	35.35
Macrolide antibiotic	Antibiotic efflux	*macB*	41.52	41.52
		mef(E)	0	24.38
		mef(F)	24.44	24.44
		mreA	37.68	37.68
		oleC	31.39	31.39
Multiple antibiotics	Antibiotic efflux	*LmrS*	45.13	45.13
		AmvA	29.57	29.81
		MexZ	36.84	36.84
		nalD	30.17	30.17
		CRP	21.81	21.81
		gadX	37.5	37.5
		evgA	23.12	23.12
		evgS	31.36	30.21
		efrA	40.94	40.94
		efrB	0	30
		lmrP	23.43	23.43
		mtrA	31.08	31.08
		aadT	27.56	27.32
		golS	36.07	36.07
	Antibiotic target alteration; antibiotic efflux	*MexR*	29.71	28.99
	Antibiotic target alteration	*RlmA(II)*	30.88	30.88
		emtA	30.97	30.97
		Erm(K)	33	33
Monobactam; cephalosporin	Antibiotic inactivation	*MIR-9*	27.07	27.07

Nitroimidazole antibiotic	Antibiotic efflux	*msbA*	32.04	32.52
Nucleoside antibiotic	Antibiotic inactivation	*SAT-4*	30.19	30.19
	Antibiotic efflux	*leuO*	24.75	24.75
Oxazolidinone antibiotic; phenicol antibiotic	Antibiotic target protection	*optrA*	34.86	34.71
Penam	Antibiotic inactivation	*EXO-1*	31.87	31.87
	Antibiotic target replacement	*mecI*	29.91	29.91
Peptide antibiotic	Antibiotic efflux	*bcrA*	35.08	35.08
		rosB	27.1	27.42
	Antibiotic target alteration	*almE*	28.49	28.49
		arnA	34.02	35.05
		bacA	40.79	41.16
		PmrF	29.36	29.36
		tsnR	30.56	30.56
	Reduced permeability to antibiotic	*LpsB*	24	29.37
Phenicol antibiotic	Antibiotic efflux	*cmlB*	27.73	27.73
		cmx	25.78	25.78
	Antibiotic inactivation	*Agrobacterium fabrum* chloramphenicol acetyltransferase	47.06	47.06
		cat86	33	0
		catQ	0	33.33
		cmlv	31.95	31.95
Phosphonic acid antibiotic	Antibiotic efflux	*mdtG*	46.72	46.72
	Antibiotic inactivation	*FosM3*	25.38	25.38
Pleuromutilin antibiotic	Antibiotic efflux	*TaeA*	43.68	43.53
Rifamycin antibiotic	Antibiotic inactivation	*rphA*	0	37.38
		rphB	42	42
	Antibiotic target protection	*HelR*	28.03	28.41
	Antibiotic efflux	*efpA*	30.33	30.33
Streptogramin antibiotic	Antibiotic inactivation	*vatD*	34.91	34.91
		vatE	33.71	33.71
		vatF	40.91	27.01
		vatF	27.01	40.91
		vatF	43.14	43.14
		VatI	42.03	40.58
Sulfonamide antibiotic	Antibiotic target replacement	*sul3*	33.73	33.73
Tetracycline antibiotic	Antibiotic efflux	*otr(B)*	29.69	29.69
		tcr3	28.14	28.14
		tet(43)	26.17	29.41
		tetA(58)	50	50.65
		tetA(60)	35.55	35.46
		tetB(58)	44.26	44.26
		tetB(60)	35.21	35.21
		TxR	25.19	25.19

	Antibiotic target alteration; antibiotic efflux	*tetR*	37.5	37.5
	Antibiotic target protection	*otr(A)*	42.25	42.25
		tet(O/W/O)	42.63	37.01
		tet(T)	37.01	29.49
		poxtA	25.96	26.16
Cationic antimicrobial peptide (CAMP)	Reduced permeability to antibiotic	*pbpX2*	100.00	100.00
		dltX	100.00	100.00
		dltA	100.00	100.00
		dltB	100.00	100.00
		dltC	100.00	100.00
		dltD	100.00	100.00
		mprF	100.00	100.00

Table S12. The susceptibility to antibiotics of *L. plantarum* 8p-a3 and DMC-S1 strains.

Antibiotic name	Dispatch 8p-a3	Dispatch DMC-S1	Antibiotic name	Dispatch 8p-a3	Dispatch DMC-S1
**Aminoglycosides**			**Oxazolidones**		
Amikacin	R	R	Linezolid	S	S
Gentamicin	R	R	**Carbapenems**		
Kanamycin	R	R	Imipenem	S	S
Streptomycin	R	R	Meropenem	S	
**Penicillins**			Ertapenem	I	
Amoxicillin	S	S	**Glycopeptides and lipopeptides**		
Ampicillin-sulbactam		S	Vancomycin	R	R
Oxacillin		R	**Macrolides, lincosamides and streptogramins**		
Benzylpenicillin		R	Clarithromycin	S	S
Ampicillin	S		Clindamycin	S	S
**Cephalosporins***			Erythromycin	S	S
Cefepime	R	R	**Fluoroquinolones**		
Cefotaxime	R	R	Norfloxacin	R	R
Ceftazidime	S	R	Ciprofloxacin	R	R
Ceftriaxone	R	R	Ofloxacin	I	
Cefazolin	I		**Other antibiotics**		
Cefoperazone	R		Rifampicin	R	S
**Tetracyclines**			Trimethoprim-sulfamethoxazole **	S	S
Tetracycline	R	R	Nitrofurantoin		S
			Cloramphenicol		S

Note: S, I and R indicate sensitive, intermediary and resistant respectively

***** Areas with less intense growth were observed around discs containing beta-lactam antibiotics (oxacillin, benzylpenicillin, cefepime, cefotaxime, ceftazidime, ceftriaxone). Microscopic analysis of fixed preparations stained using the Gram method revealed a change in bacterial cell morphology characteristic of beta-lactam antibiotics. After replanting on a nutrient medium without an antibiotic, the cell shape was restored.

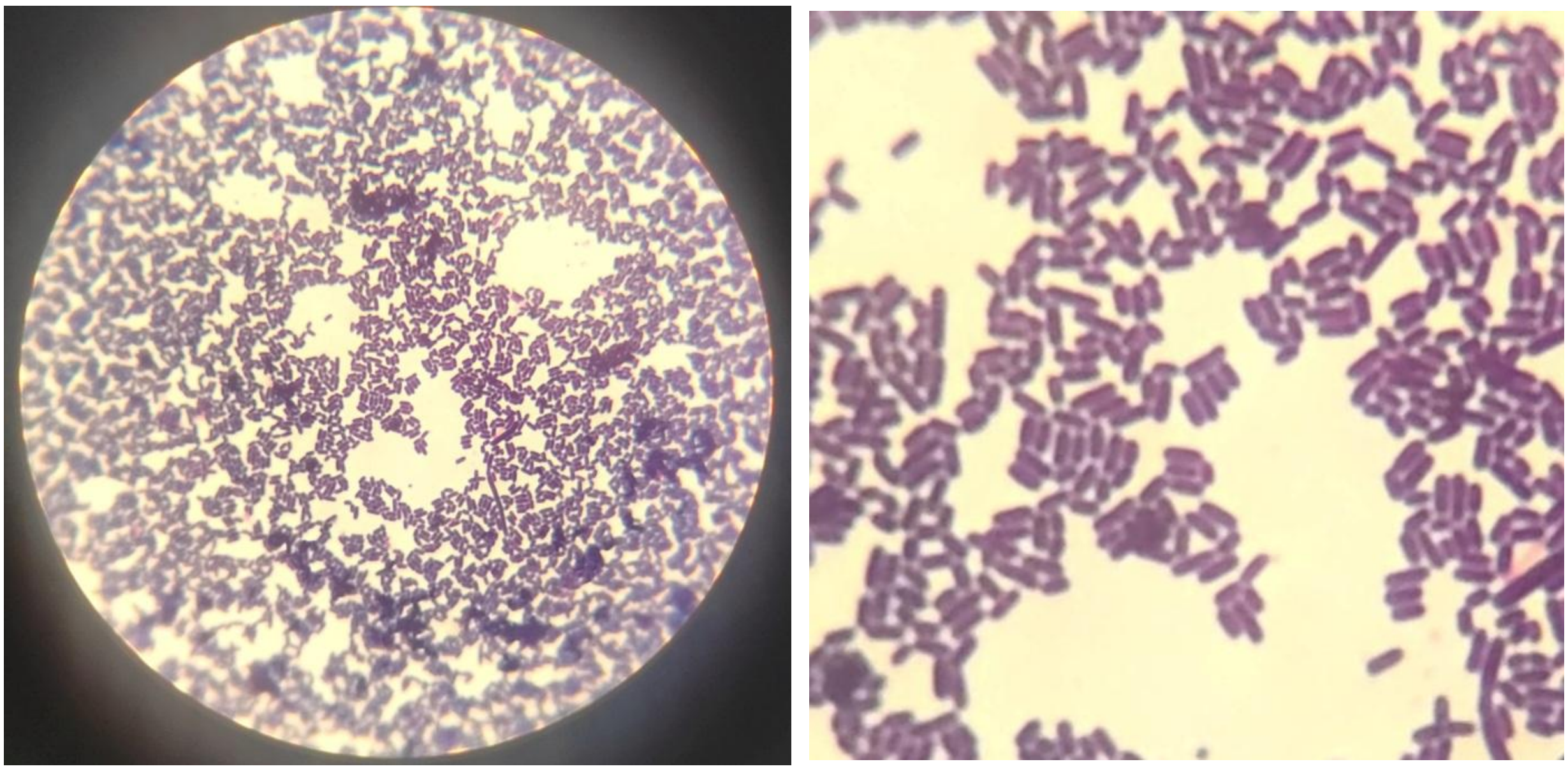

A – without an antibiotic

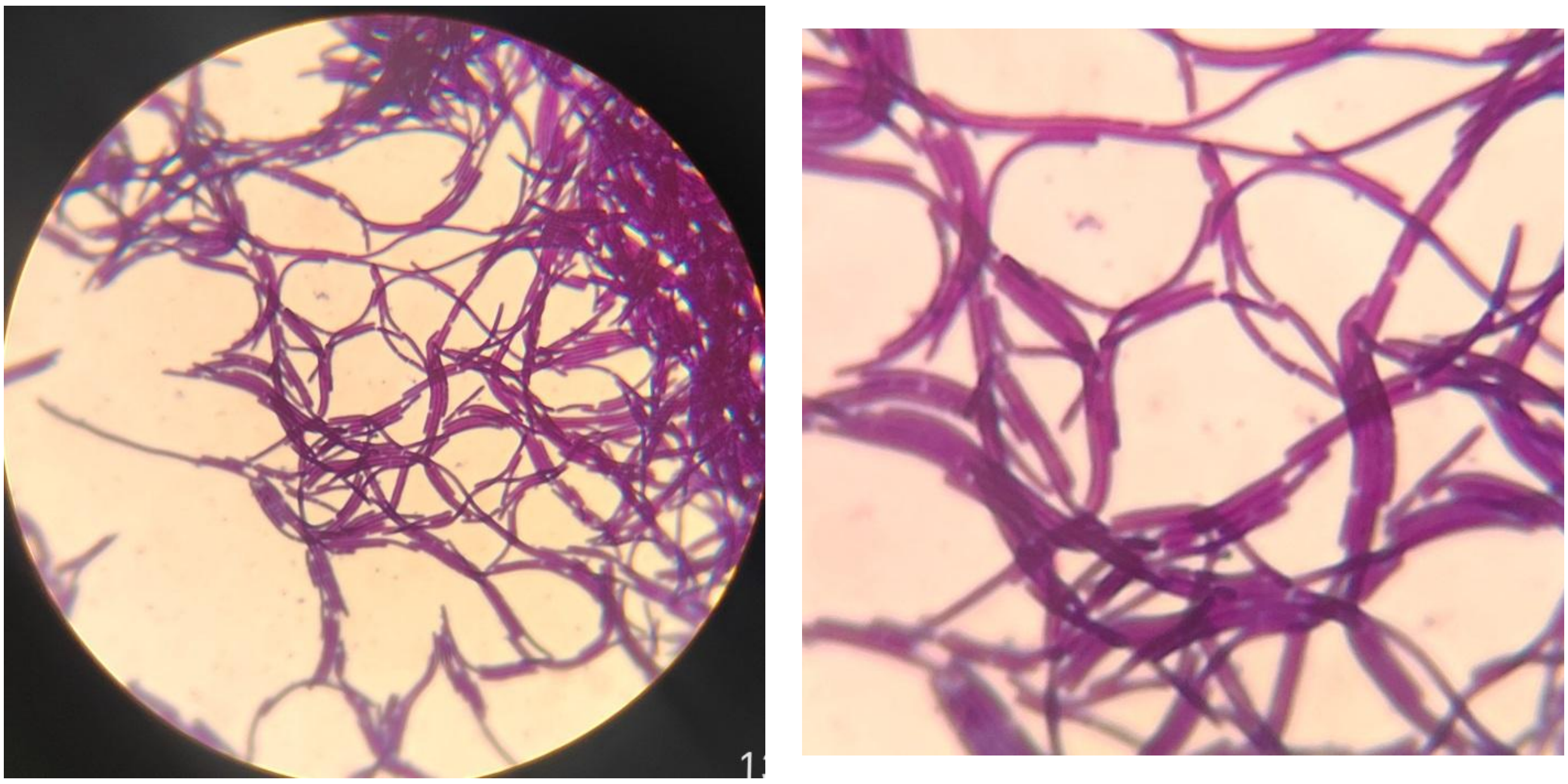

B – with an antibiotic

******When determining sensitivity to sulfonamides and their combination with trimethoprim, the boundary of the growth suppression zone was taken into account at the level of 80% growth inhibition in accordance with the guidelines. This is due to the fact that under the action of these drugs, 1-2 cycles of microbial proliferation can be completed before complete suppression of growth.

Table S13. Virulence genes identified in genomes of *L. plantarum* 8p-a3 and DMC-S1

VFDB id	Gene	Process	Host	% identity	
				8p-a3	DMC-S1
VFG012103(gb|WP_003514589)	*groEL*	Adherence (VFC0001)	*Clostridium thermocellum* ATCC 27405	68.99	68.99
VFG016490(gb|NP_975163)	*tuf*	Adherence (VFC0001)	*Mycoplasma mycoides subsp. mycoides* SC str. PG1	71.83	71.83
VFG043573(gb|NP_219906)	*dnaK*	Adherence; porin (VF0713)	*Chlamydia trachomatis* D/UW-3/CX	63.46	63.46
VFG005871(gb|WP_002991444)	*hasC*	Capsule (VF0144) - Immune modulation (VFC0258)	*Streptococcus pyogenes* MGAS10270	75.68	75.68
VFG006022(gb|WP_002947383)	*rfbB*	Capsule (VF0144) - Immune modulation (VFC0258)	*Streptococcus thermophilus* LMD-9	67.45	70.62
VFG002182(gb|WP_002376666)	*cpsI*	Capsule (VF0361) - Immune modulation (VFC0258)	*Enterococcus faecalis* V583	64.67	65.04
VFG002182(gb|WP_002376666)	*cpsI*	Capsule (VF0361) - Immune modulation (VFC0258)	*Enterococcus faecalis* V583	62.90	62.63
VFG045688(gb|WP_002294134)	*cpsA/uppS*	Capsule (VF0361) - Immune modulation (VFC0258)	*Enterococcus faecium* Aus0004	69.36	69.36
VFG048851(gb|WP_015958700)	*gndA*	Capsule (VF0560) - Immune modulation (VFC0258)	*Klebsiella pneumoniae subsp. pneumoniae* MGH 78578	63.35	63.35
VFG016432(gb|WP_000059856)	BALH_RS26235	Polysaccharide capsule (VF0659) - Immune modulation (VFC0258)	*Bacillus thuringiensis* str. Al Hakam	61.28	61.28
VFG006826(gb|NP_464902)	*lisR*	Regulation (VFC0301)	*Listeria monocytogenes* EGD-e	76.75	76.75
VFG026980(gb|YP_005923858)	*sigA/rpoV*	Regulation (VFC0301)	*Mycobacterium tuberculosis* RGTB423	63.04	63.04
VFG005582(gb|WP_002897814)	*eno*	Streptococcal enolase (VF1060) - Exoenzyme (VFC0251)	*Streptococcus sanguinis* SK36	71.99	71.99
VFG000077(gb|NP_465991)	*clpP*	Stress survival (VFC0282)	*Listeria monocytogenes* EGD-e	69.79	69.79
VFG006815(gb|WP_010959001)	*bsh*	Stress survival (VFC0282)	*Listeria monocytogenes* serotype 4b str. F2365	67.28	67.90
VFG037110(gb|WP_002224229)	*msrA/B(pilB)*	Stress survival (VFC0282)	*Neisseria meningitidis* M01-240149	64.75	64.75
WP_001284639.1	*hupB*	Stress survival (VFC0282)	*Streptococcus pneumoniae* R6	76.92	76.92

Table S14. Genes related with probiotic characteristics in genomes *of L. plantarum* 8p-a3 and DMC-S1

Gene	Function	Number of genes	
		8p-a3	DMC-S1
**Heat stress**			
htpX	heat shock protein HtpX	1	1
hrcA	heat-inducible transcriptional repressor	1	1
hslO	molecular chaperone Hsp33	1	1
hslU	ATP-dependent HslUV protease ATP-binding subunit HslU	1	1
HSP20	HSP20 family protein	3	3
dnaK	HSPA9; molecular chaperone DnaK	1	1
dnaJ	molecular chaperone DnaJ	1	1
ctsR	transcriptional regulator of stress and heat shock response	1	1
grpE	molecular chaperone GrpE	1	1
groEL	HSPD1; chaperonin GroEL	1	1
groES	HSPE1; chaperonin GroES	1	1
lon	Lon-like protease	1	1
clpB	ATP-dependent Clp protease ATP-binding subunit ClpB	1	1
clpC	ATP-dependent Clp protease ATP-binding subunit ClpC	1	1
clpE	ATP-dependent Clp protease ATP-binding subunit ClpE	1	1
clpL	ATP-dependent protease ClpL	1	1
clpX	ATP-dependent Clp protease ATP-binding subunit ClpX	1	1
clpP	ATP-dependent Clp endopeptidase proteolytic subunit ClpP	1	1
htrA	Serine protease HtrA	1	1
**Cold stress**			
cspA	Cold-shock protein	4	3
**Acid stress**			
atpA	F-type H+/Na+-transporting ATPase subunit alpha	1	1
atpB	F-type H+-transporting ATPase subunit a	1	1
atpE	F-type H+-transporting ATPase subunit c	1	1
atpF	F-type H+-transporting ATPase subunit b	1	1
atpH	F-type H+-transporting ATPase subunit delta	1	1

atpG	F-type H+-transporting ATPase subunit gamma	1	1
atpD	F-type H+/Na+-transporting ATPase subunit beta	1	1
atpC	F-type H+-transporting ATPase subunit epsilon	1	1
gadB	gadB, gadA, GAD; glutamate decarboxylase	1	1
nhaC	Na+:H+ antiporter, NhaC family	2	2
copA	Copper transporting ATPase	1	1
pgk	Phosphoglycerate kinase	1	1
gpmB	Phosphoglycerate mutase GpmB	1	1
guaA	GMP synthase [glutamine-hydrolyzing]	1	1
ldh_1	L-lactate dehydrogenase 1	5	5
plsC	1-acylglycerol-3-phosphate O-acyltransferase	1	1
pyk	Pyruvate kinase	1	1
recA	Protein RecA	1	1
relA	GTP pyrophosphokinase	2	2
uvrA/uvrA_1	uvrABC system protein A	1	1
**Osmotic stress**			
glpF	Glycerol uptake facilitator	4	4
**Bile tolerance**			
bsh	choloylglycine hydrolase	3	3
ppaC	manganese-dependent inorganic pyrophosphatase	1	1
srtA	Sortase A	1	1
cfa	cyclopropane-fatty-acyl-phospholipid synthase	2	2
arcB	Ornithine carbamoyltransferase	1	1
argS	Arginine--tRNA ligase	1	1
pgk	Phosphoglycerate kinase	1	1
dps	DNA protection during starvation protein	2	2
glnA	Glutamine synthetase	1	1
nagB	Glucosamine-6-phosphate deaminase	1	1
pepO	Endopeptidase PepO	1	1
pyrG	CTP synthase	1	1
rplD	50S ribosomal protein L4	1	1
rplE	50S ribosomal protein L5	1	1
rplF	50S ribosomal protein L6	1	1

rpsC	30S ribosomal protein S3	1	1
rpsE	30S ribosomal protein S5	1	1
luxS	S-ribosylhomocysteine lyase	1	1
**Adhesion**			
mapA	maltose phosphorylase	4	4
lspA	lipoprotein signal peptidase II	1	1
tuf	elongation factor Tu	1	1
gpr	L-glyceraldehyde 3-phosphate reductase	1	1
tpiA	triosephosphate isomerase (TIM)	1	1
gapA	glyceraldehyde 3-phosphate dehydrogenase (phosphorylating)	1	1
bgaB	beta-galactosidase	1	1
srtA	sortase A	1	1
cwaA	cell wall-anchored adhesion-associated protein	1	0
msa	mannose-specific adhesin LPXTG-motif cell wall anchor	0	1
mub	mucus-binding protein precursor LPXTG-motif cell wall anchor, lp_1643	1	0
eno	enolase	3	2
pgi	glucose-6-phosphate isomerase	1	1
LPXTG	surface protein (LPXTG motif)	5	6
msl	cell surface protein precursor mannose-specific lectin	1	1
exoA	Exodeoxyribonuclease III	1	1
pycA	Pyruvate carboxylase	1	1
**Antioxidant**			
katE	catalase	1	1
nrdH	glutaredoxin	1	1
gpx	glutathione peroxidase	1	1
mntH	manganese transport protein	2	2
mntB	manganese transport system permease protein	1	1
ndh	NADH dehydrogenase	1	1
npr	NADH peroxidase	2	2
spxB	pyruvate oxidase	3	3
tpx	thiol peroxidase	1	1
trxA	thioredoxin	2	2

trxB	thioredoxin reductase	1	1
nox	NADH oxidase	2	2
msrA	peptide-methionine (S)-S-oxide reductase	3	3
msrB	peptide-methionine (R)-S-oxide reductase	1	1
**Immunomodulation**			
dltA	D-alanine—poly(phosphoribitol) ligase subunit 1	1	1
dltB	membrane protein involved in D-alanine export	1	1
dltC	D-alanine—poly(phosphoribitol) ligase subunit 2	2	2
ackA	acetate kinase	1	1
mprF	bifunctional lysylphosphatidylglycerol flippase/synthetase MprF	1	1
**Tannin tolerance**			
tanA	subtype A tannase	1	0
tanB	subtype B tannase	1	1
**Gut persistence**			
celB	PTS system, cellobiose-specific EIIC component	2	2
**Riboflavin biosynthesis**			
ribA	**GTP cyclohydrolase-2**	1	1
ribB	**GTP cyclohydrolase-2**	1	1
ribG	Bifunctional diaminohydroxyphosphoribosylaminopyrimidine deaminase/5-amino-6-(5-phosphoribosylamino)uracil reductase RibD	1	1
ribH	6,7-dimethyl-8-ribityllumazine synthase	1	1
ribF	Riboflavin biosynthesis protein	1	1
**Folate biosynthesis**			
aroA	3-phosphoshikimate 1-carboxyvinyltransferase	1	1
aroB	3-dehydroquinate synthase	1	1
aroC	Chorismate synthase	1	1
aroD	3-dehydroquinate dehydratase	1	1
aroE	Shikimate dehydrogenase (NADP(+))	1	1
aroF	3-deoxy-7-phosphoheptulonate synthase	1	1
aroK	Shikimate kinase	1	1
folE	GTP cyclohydrolase 1	1	1

folK	2-amino-4-hydroxy-6-hydroxymethyldihydropteridine diphosphokinase	1	1
folB	7,8-dihydroneopterin aldolase	1	1
folQ	Dihydroneopterin triphosphate pyrophosphohydrolase	1	1
folC	Tetrahydrofolate synthase	1	1
folP	Dihydropteroate synthase	1	1
dfrA	Dihydrofolate reductase	1	1
**Cobalamin biosynthesis**			
cbiM	Cobalamin biosynthesis protein CbiM	1	1
cbiO	Energy-coupling factor transporter ATP-binding protein	1	1
cbiQ	Energy-coupling factor transporter transmembrane protein	1	1
cbiB	Cobalamin biosynthesis protein	1	1

Table S15. Glycosyl hydrolase (GH) families genes involved in mucin degradation in *L. plantarum* 8p-a3 and DMC-S1 genomes

Glycoside Hydrolases	EC	Activity name	Protein id	
			8p-a3	DMC-S1
GH2	3.2.1.23	b-galactosidase	WP_046947528.1	WP_022638262.1
GH42	3.2.1.23	b-galactosidase	WP_013356026.1	WP_013356026.1
GH20	3.2.1.52	beta-N-acetylhexosaminidase	WP_003641589.1	WP_013356079.1
GH85	3.2.1.96	mannosyl-oligosaccharide endo-b-1,4-N-acetylglucosaminidase	WP_003643680.1	-

Table S16. Bacteriocin related genes in *L. plantarum* strains

Gene	Function	8p-a3	DMC-S1	8P-A3	NC8	dm	EM	DR7	DOMLa	12_3	5_2	B21	Q7	JBE490	SRCM100995
plnA	Induction pheromone	+	+	+	-	+	+	-	-	-	+	+	-	-	+
plnB	Histidine protein kinase	+	+	+	-	+	+	+	+	-	+	+	-	+	+
plnC	Response regulator	+	+	+	-	+	+	-	-	-	-	+	-	-	-
plnD	Response regulator	+	+	+	+	+	+	+	+	+	+	+	-	+	+
plnE	Prebacteriocin with GC leader	+	+	+	+	+	+	+	+	-	+	+	-	+	+
plnF	Prebacteriocin with GC leader	+	+	+	+	+	+	+	+	-	+	+	-	+	+
plnG (lanT)	ABC Transporter	+	+	+	+	+	+	+	+	-	+	+	-	+	+
plnH (hlyD)	Accessory protein	+	+	+	+	+	+	+	+	-	+	+	-	+	+
plnI	Putative immunity protein containing a protease CAAX signature	+	+	+	+	+	+	+	+	-	+	+	-	+	+
plnJ	Prebacteriocin with a GG-leader	+	+	+	+	+	+	-	-	-	+	+	-	-	+
plnK	Prebacteriocin with a GG-leader	-	+	-	+	+	+	-	+	+	+	+	-	-	+
plnL	Putative immunity protein containing a protease CAAX signature	+	+	+	+	+	+	+	+	+	+	+	-	-	-
plnM	Unknown function	-	+	-	+	+	+	-	-	-	+	+	-	-	+
plnN	Putative prebacteriocin with a GG-leader	-	+	-	-	+	+	-	-	-	-	+	-	-	-
plnO (glyS)	Glycosyl transferase group 2 family	-	+	-	-	+	+	-	-	-	-	+	-	-	-
plnP	Protease CAAX family	-	+	-	+	+	+	-	-	-	+	+	-	-	+
plnQ	Putative protein with unknown function	+	-	+	-	-	+	-	-	-	-	+	-	-	-
plnR	Putative protein with unknown function	-	+	-	+	+	+	-	-	-	+	+	-	-	+
plnS	Putative protein with unknown function	-	-	-	+	-	-	-	-	-	+	-	-	-	-
plnT	Putative protein containing a protease CAAX signature	+	+	+	+	+	+	+	+	+	+	+	-	+	+
plnU	Putative protein containing a protease CAAX signature	+	+	+	+	+	+	+	+	+	+	+	-	+	+
plnV	Putative protein containing a protease CAAX signature	+	+	+	+	+	+	+	+	+	+	+	-	+	+
plnW	Putative protein containing a protease CAAX signature	+	+	+	+	+	+	+	+	+	+	+	-	+	+
plnX	Homologous to plasmid maintenance system killer proteins	-	-	-	+	-	-	-	+	-	+	+	-	-	+
plnY	Homologous to plasmid maintenance system antidote proteins	-	-	-	+	-	-	-	+	-	-	+	-	-	-
nc8α	Prebacteriocin with a GG-leader	+	-	+	+	-	-	-	-	-	+	-	-	-	+
nc8β	Prebacteriocin with a GG-leader	+	-	+	+	-	-	-	-	-	+	-	-	-	+

nc8c	Putative immunity protein	+	-	+	+	-	-	-	-	-	+	-	-	-	-
nc8-M	Unknown function	-	-	-	+	-	-	-	-	-	-	-	-	-	-
nc8-IF	Induction pheromone	-	-	-	+	-	-	-	-	-	-	-	-	-	-
nc8-HK	Histidine protein kinase	-	-	-	+	-	-	-	-	-	-	-	-	-	-
Enterocin-x-β	Prebacteriocin with GC leader	+	-	+	-	-	-	+	-	+	-	-	-	-	-
Lactococcin	Prebacteriocin with GC leader	+	-	+	-	-	-	+	-	+	-	-	-	-	-
Bovicin-255	Prebacteriocin	-	-	-	-	-	+	-	-	-	-	-	-	-	-
Pentocin	Prebacteriocin	-	-	-	-	-	-	-	-	-	-	+	-	-	-
Pediocin	Prebacteriocin	-	-	-	-	-	-	-	-	-	-	-	+	-	+
Pediocin_PA	Prebacteriocin	-	-	-	-	-	-	-	-	-	-	-	+	-	-
Coagulin A	Prebacteriocin	-	-	-	-	-	-	-	-	-	-	-	-	-	+

Table S17. Oligonucleotides used for the quantitative PCR

#	Target gene	Gene product	Oligonucleotide sequence, 5' – 3'
1	*ackA*	Acetate kinase	For gta ttc ctt acg att att atg aga aat ttg gcg<br>Rev gtg atc gaa gca ccc gac cc
2	*mprF*	L-O-lysylphosphatidyl glycerol synthase	For ctg atg ttg ttg atg tca acg gtc c<br>Rev gac ccg gcc agc agt tcc c
3	*tanA*	Tannase A	For cta cta gtg atg cag tgg ccg g<br>Rev aca aag ctt tgg tcc acg tac ctt g
4	*xhlA*	Hemolysin XhlA family protein	For ata ttc aaa agg atg tgg ctg cca cc<br>Rev cac gcc act aac caa gac acc
5	*ldh1*	D-Lactate dehydrogenase	For tta gct aag caa gac ttc cgt tgg g<br>Rev gta acc gat aac ctt agc gcc gaa g
6	*ldh2*	D-Lactate dehydrogenase	For gtc ggt ggc aca aac cat gcg<br>Rev gac ccg cac caa taa tac caa cgg
7	*rpoC*	RNA polymerase	For tct tga acc gcg ccc cta cg<br>Rev gtg aat agc cat ttg gtc ccc atc g
8	*Prx5*	Peroxyredoxin5	For ccg atg agc tga agt cca ag<br>Rev ttg ccg ttc tcc acc acc ag
9	*dFOXO*	Transcription factor FOXO	For tag cag tgc cgg atg gaa gaa c<br>Rev acc ctc ata aag cgg ttg tgc ag
10	*Dro*	Drosocin	For tca gtt cga ttt gtc cac ca<br>Rev gat ggc agc ttg agt cag gt
11	*Mtk*	Metchnikowin	For tcg ccc ttc aat cct aac caa cc<br>Rev acg aca tca gca gtg tga att tcc
12	*Def*	Defensin	For gtt ctt cgt tct cgt ggc tat cg<br>Rev atc cac atc gga aac tgg ctg ag
13	*Drs*	Drosomycin	For aag tac ttg ttc gcc ctc ttc gc<br>Rev aca ggg acc ctt gta tct tcc g
14	*CecA1*	Cecropin A1	For tcg ctc aga cct cac tgc aat atc<br>Rev tgt cca atg gtg atg gcc aga atg
15	*βTub56D*	βTub56D	For ggc caa ctg aac gct gat ct<br>Rev aag ccg ggc atg aag aag tg
16	*eEF1α1*	eEF1α1	For agg gca aga agt agc tgg ttt gc<br>Rev gct gct act act gcg tgt tgt tg
17	*cat*	Catalase	For ttc ctg gat gag atg tcg cac t<br>Rev ttc tgg gtg tga atg aag ctg g
18	*sesB*	Mitochondrial adenine nucleotide transferase	For ctc gct tgg ctg ctg ata ctg g<br>Rev ggc ggc acg gta gat gat gat g
19	*rpr*	Reaper	For tcg caa gcc atc gca atg agg<br>Rev cgg ctg atg agt ggt gac tgt g

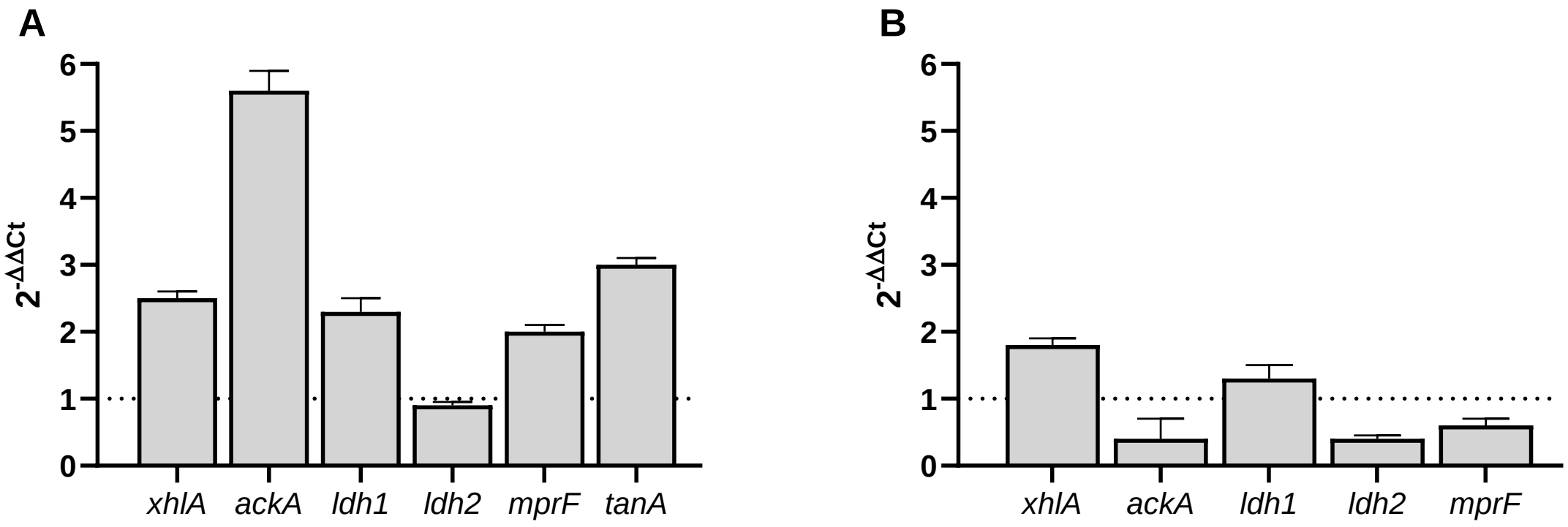

Fig. S1. Expression of *xhlA*, *ackA*, *ldh1*, *ldh2*, *mprF*, *tanA* genes. Variation of $2^{-\Delta\Delta Ct}$ values of strains growing at 37°C/25°C (A) in *L. plantarum* 8p-a3 and (B) in *L. plantarum* DMC-S1.

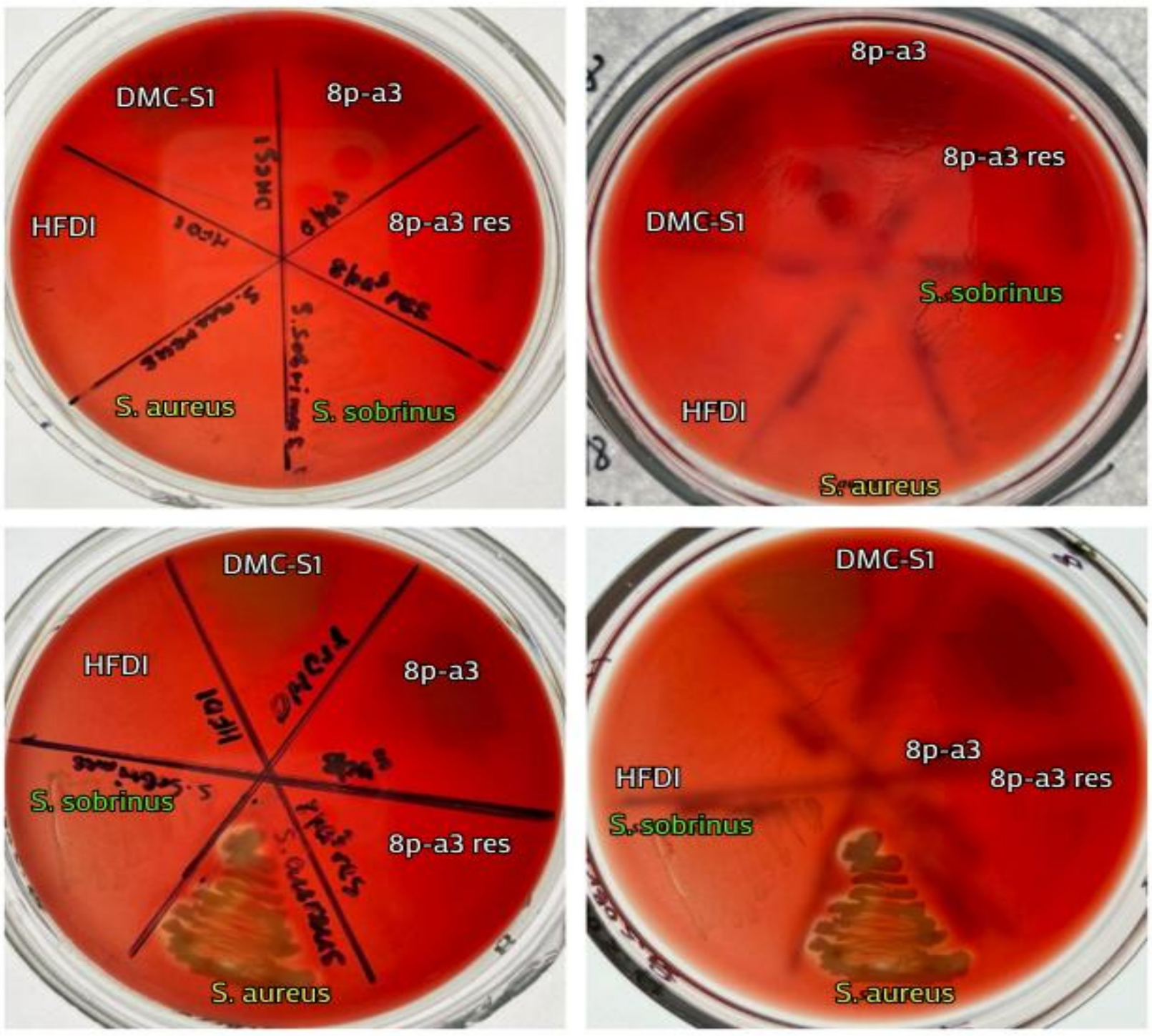

Fig. S2. Hemolytic activity of *L. plantarum* strains *in vitro.*
Staphylococcus aureus and *Streptococcus sobrinus* (causing β- and α-hemolysis, respectively) were used as references. *L. plantarum* DMC-S1 and 8p-a3 cause α-hemolysis; strain HFD1 does not exhibit hemolytic activity.

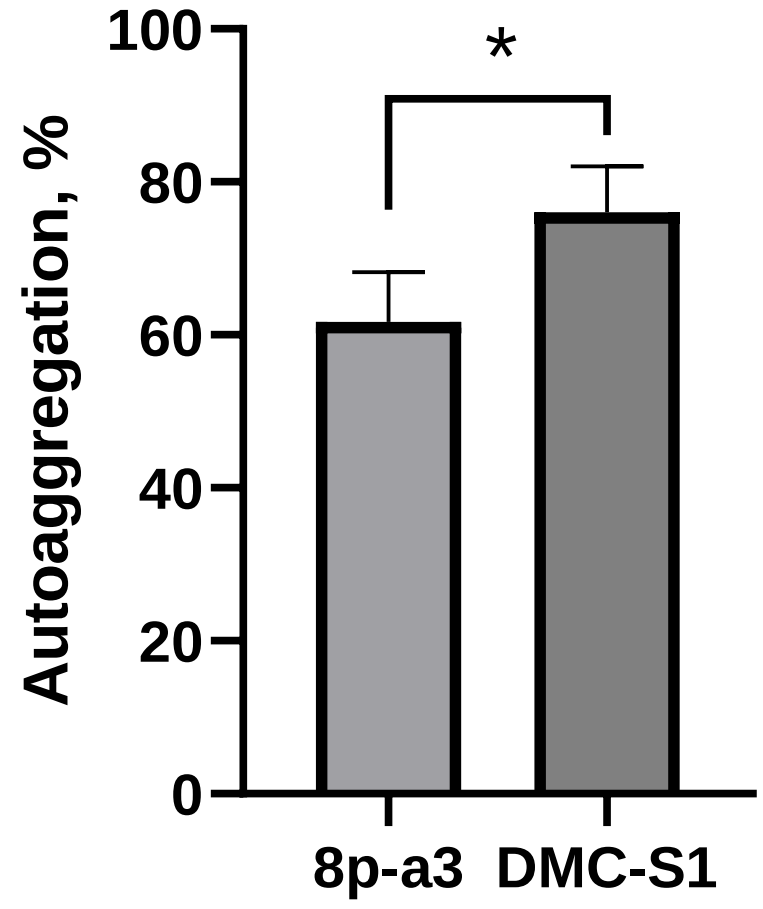

Fig. S3. The autoaggregation features of *L. plantarum* strains *in vitro*. Asterisk denote statistically significant difference (t-test, $p < 0.05$, n=5). Data are shown as mean±SD.

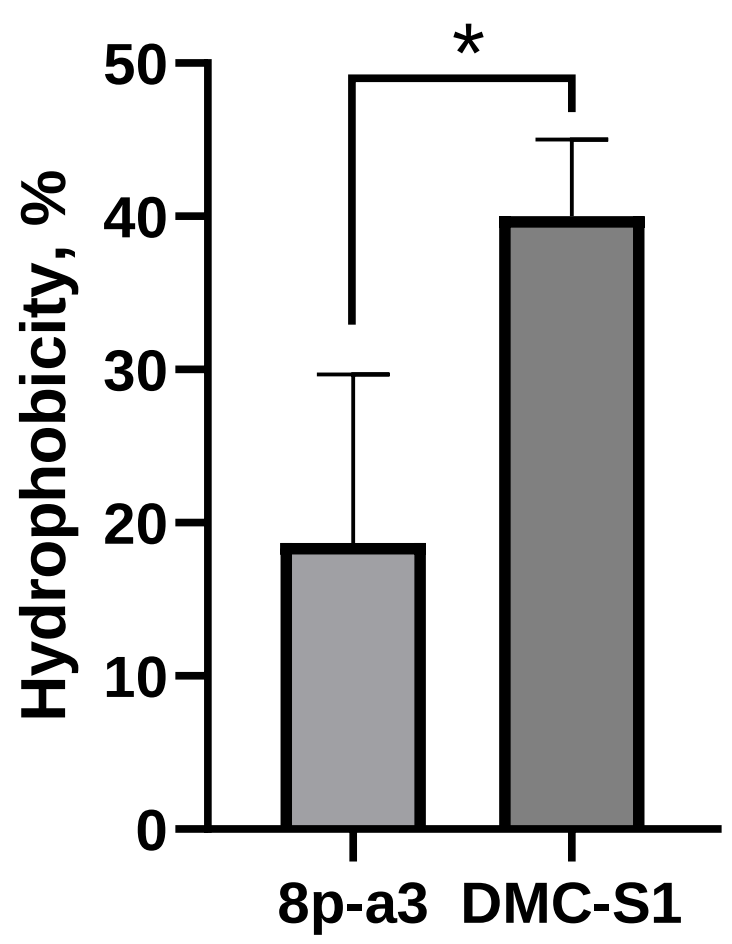

Fig. S4. Adhesive features of *L. plantarum* strains *in vitro.* Hexadecane adhesion indicates the hydrophobicity/hydrophilicity of the cell surface (the higher the level of adhesion on hexadecane, the higher the adhesive ability of the bacterium in the intestine). Based on hydrophobicity, the strains are divided into 3 groups: with low adhesion (0-35%), medium (36-70%) and high (71-100%). Strain DMC-S1 exhibits an average level of adhesion, while strain 8p-a3 exhibits a low level. DMCS1 - *L. plantarum* DMC-S1, 8p-a3 - *L. plantarum* 8p-a3. Asterisk denote statistically significant difference (t-test, $p < 0.05$, n=5). Data are shown as mean±SD.

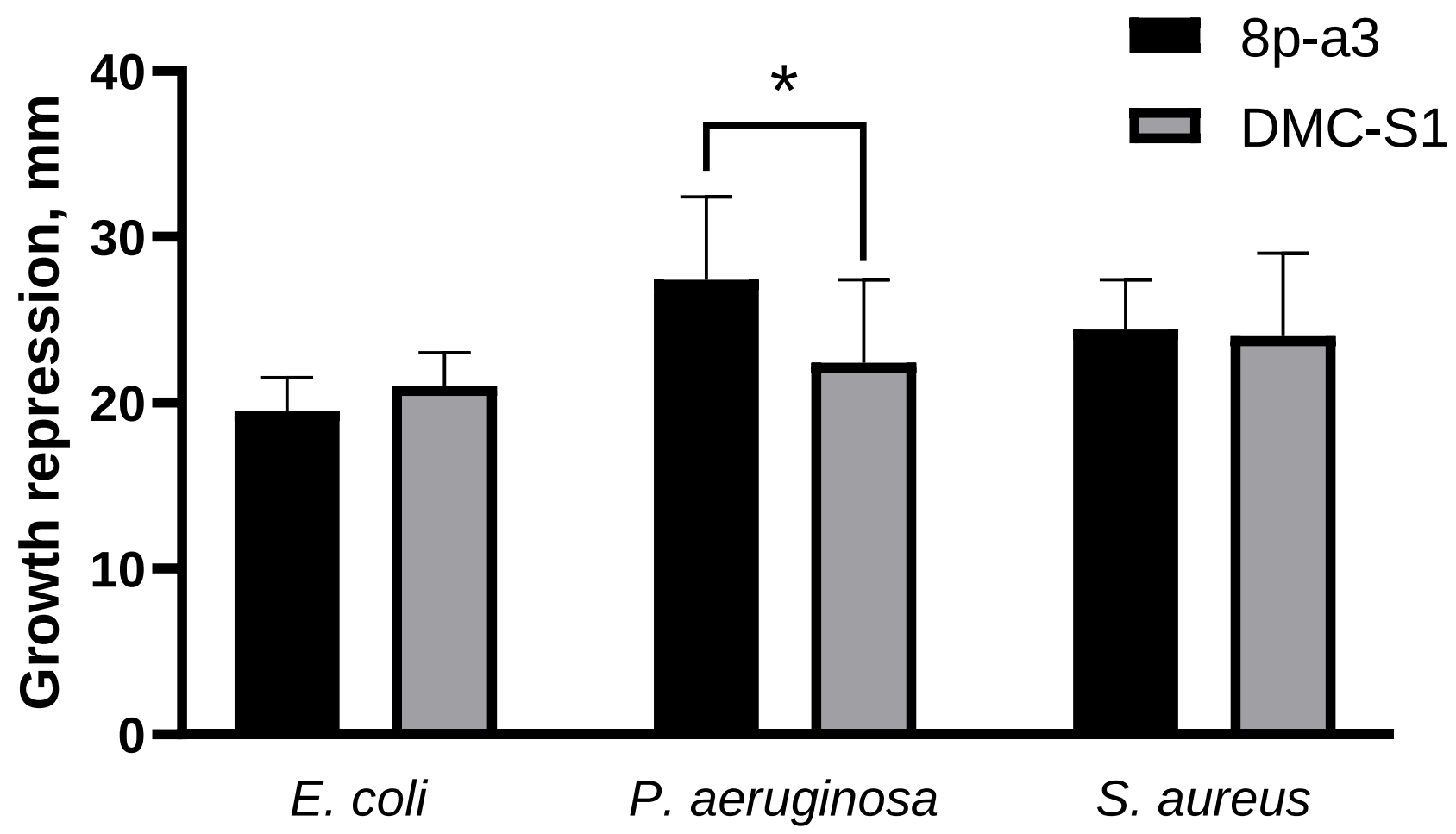

Fig. S5. Antagonistic activity of *L. plantarum* strains against pathogenic bacteria in spot-on-lawn test. 8p-a3, DMC-S1 - *L. plantarum* 8p-a3, *L. plantarum* DMC-S1, respectively. Asterisk denote statistically significant difference (multiple t-test with Holm-Sidak post hoc test, $p < 0.05$, n=3). Data are shown as mean±SD.

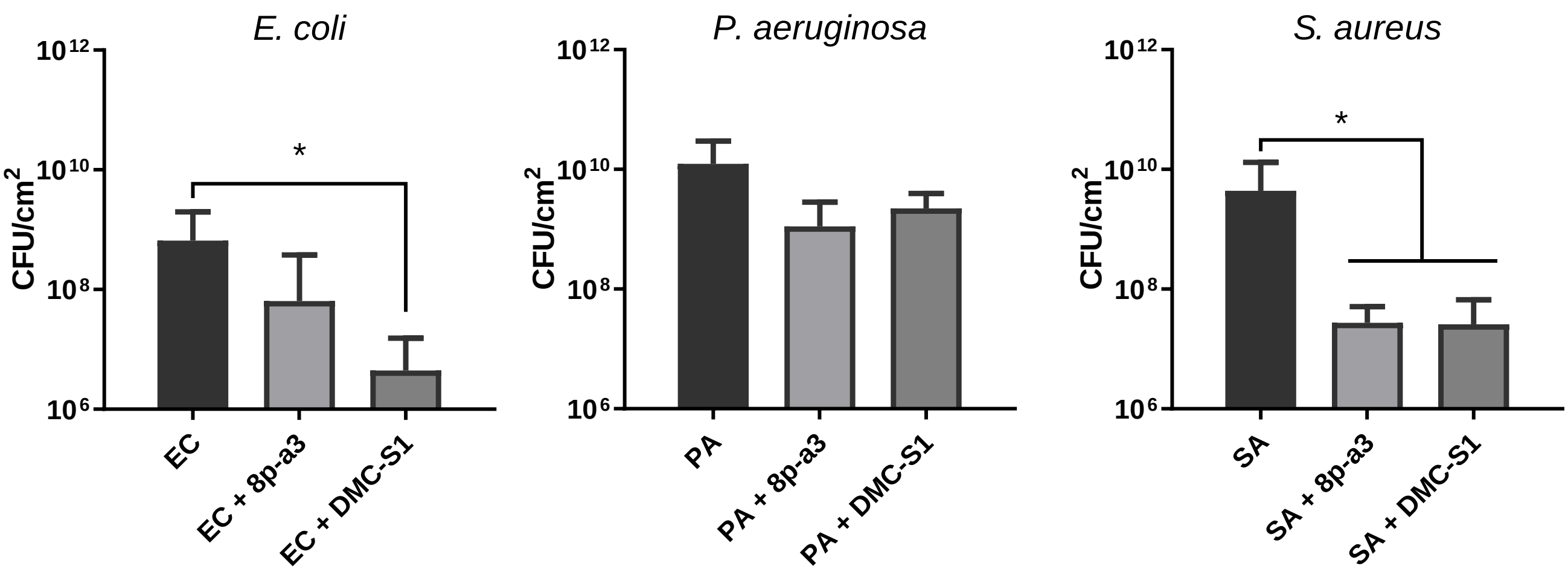

Fig. S6. Antagonistic activity of *L. plantarum* strains against pathogenic bacteria in growing biofilms. 8p-a3, DMC-S1 - *L. plantarum* 8p-a3, *L. plantarum* DMC-S1, respectively. EC, PA, SA - *E. coli*, *P. aeruginosa*, *S. aureus*, respectively. Asterisk denote statistically significant difference (Kruskal-Wallis test with Dunns post hoc test, $p < 0.05$, n=5). *L. plantarum* and opportunistic bacteria were grown together under static conditions for 48 h in BM broth with minimal amount of glucose (0.2%) to minimize acidification. Then CFUs were differentially counted on Endo agar (for *E. coli*), mannitol-salt agar (for *S. aureus*) and cetrimide agar (for *P. aeruginosa*). Data are shown as medians±IQR.

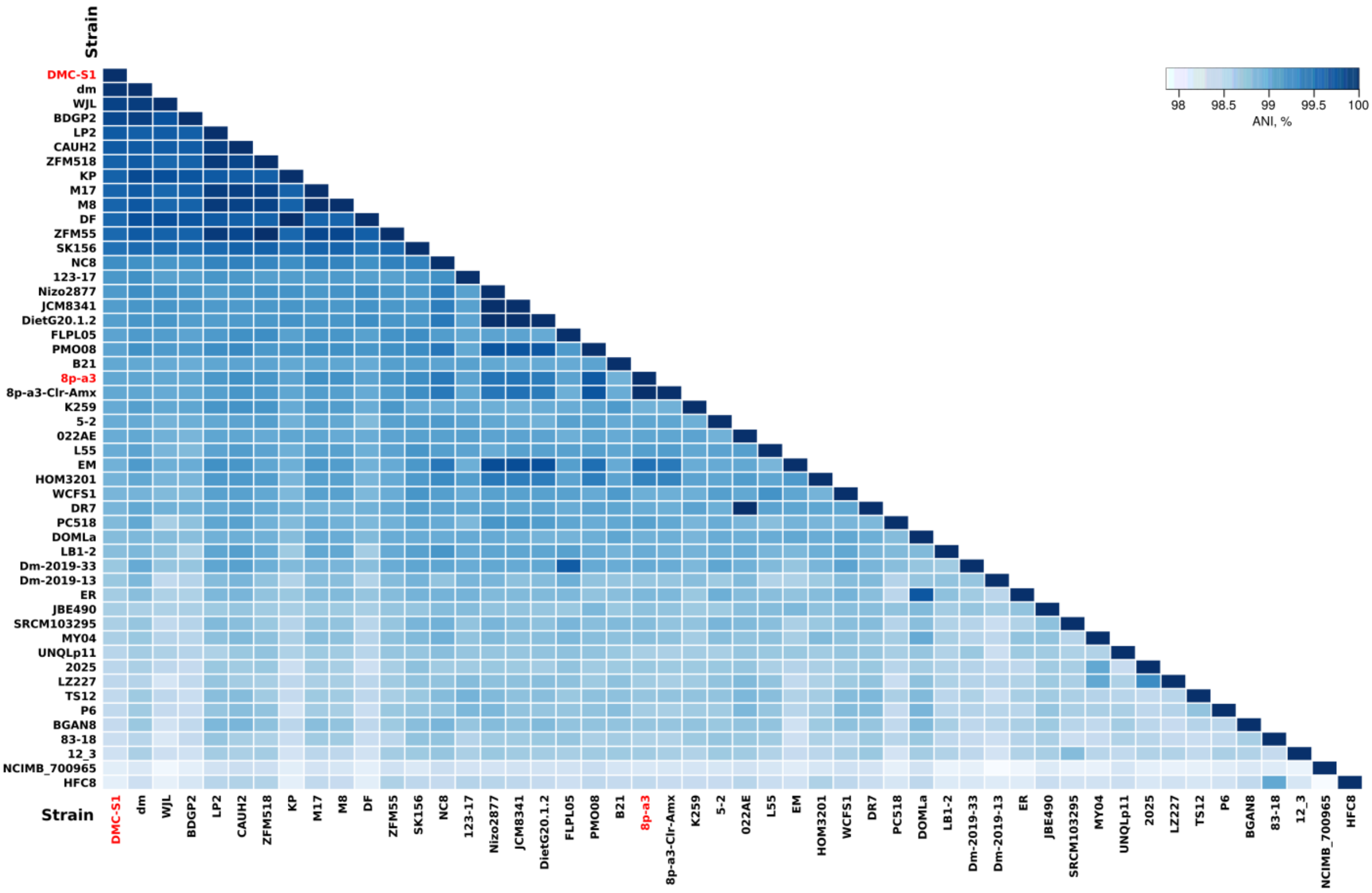

Fig. S7. Heatmap of pairwise average nucleotide identity (ANI) among 50 genomes of *L. plantarum* strains (including DMC-S1 and 8p-a3 isolated from *D. melanogaster* and the commercial probiotic “Lactobacterin”, respectively).

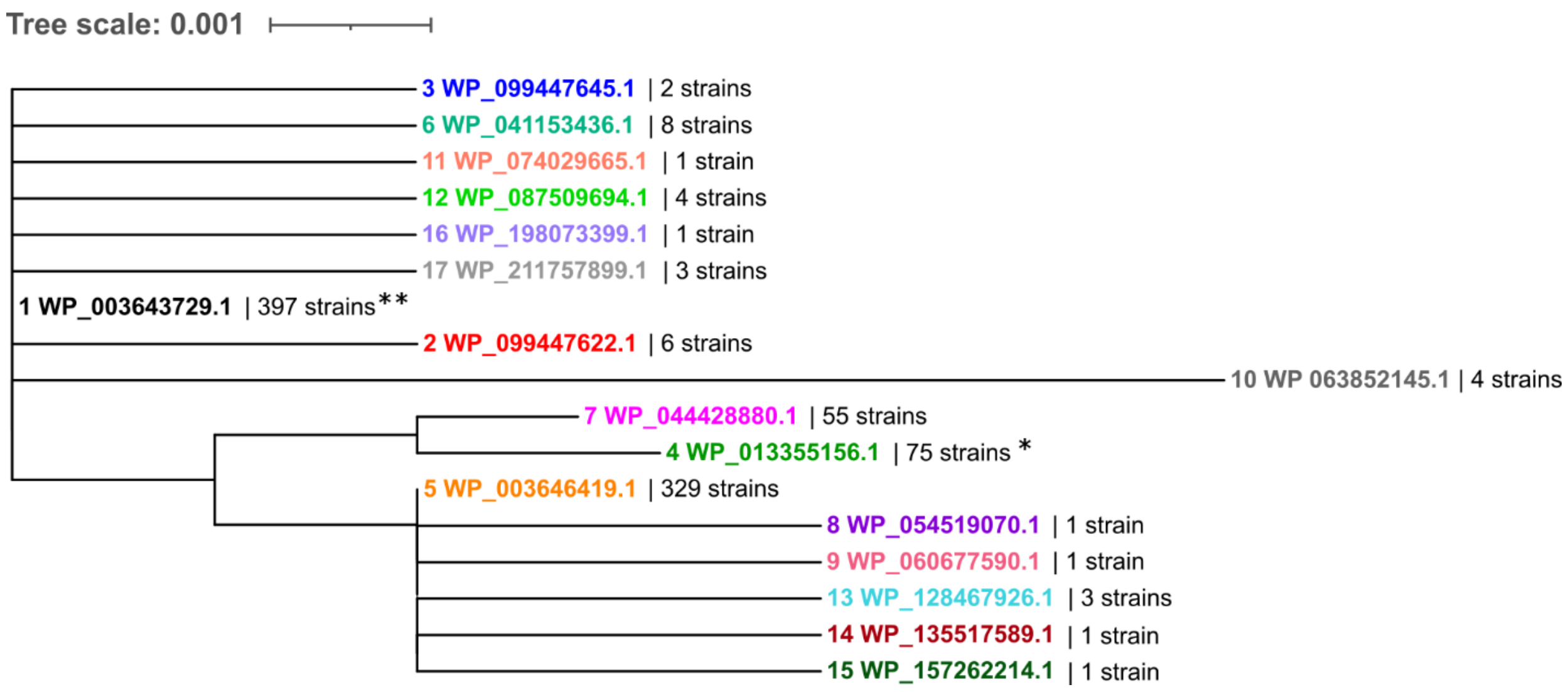

Fig. S8. The phylogenetic trees of *L. plantarum* strains is based on the amino acid sequence of the *ackA* gene. The various versions of the gene (1-17) are indicated by different colors. The number of strains exhibiting the corresponding gene version is indicated on the right, according to data from the National Center for Biotechnology Information (NCBI). *sequence variant in DMC-S1 strain, **sequence variant in 8p-a3 strain.

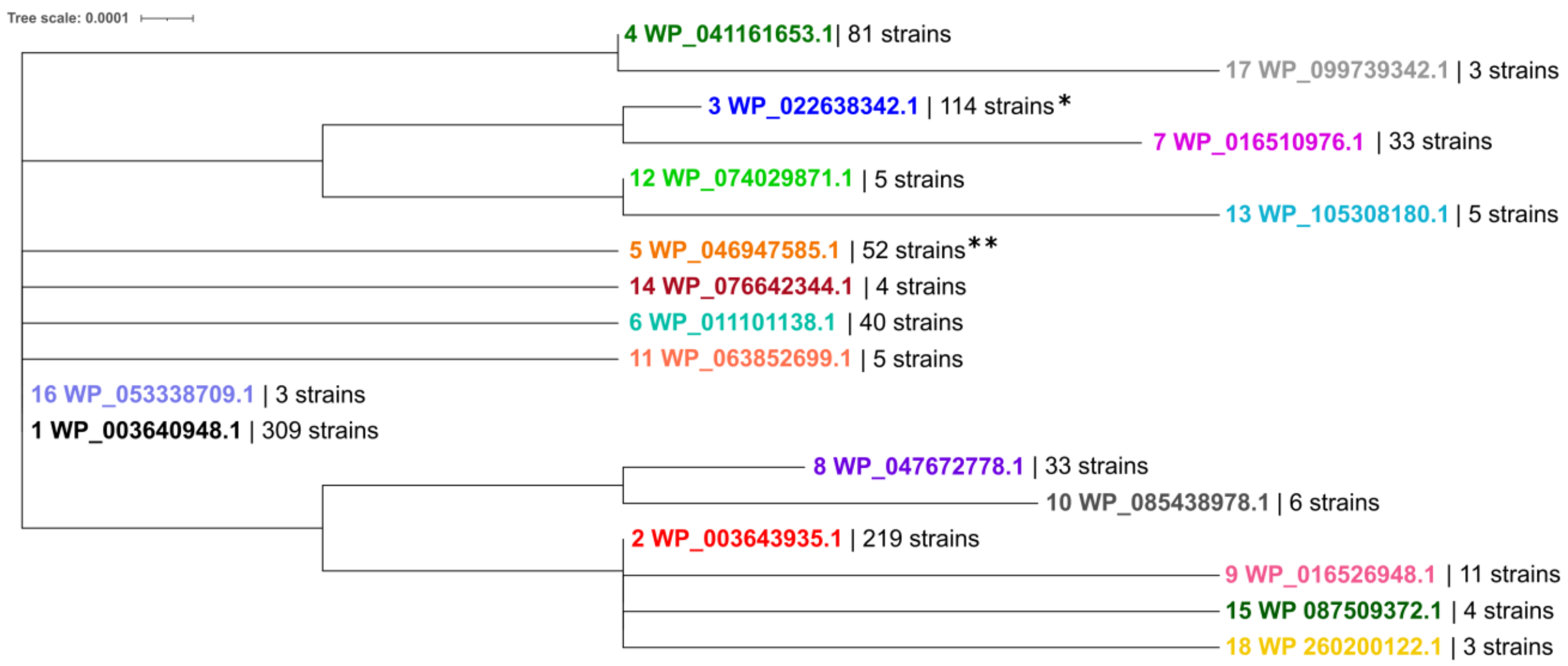

Fig. S9. The phylogenetic tree of *L. plantarum* strains is based on the amino acid sequence of the most abundant 18 types of *mprF* gene. The various versions of the gene (1-18) are indicated by different colors. The number of strains exhibiting the corresponding gene version is indicated on the right, according to data from the National Center for Biotechnology Information (NCBI). *sequence variant in DMC-S1 strain, **sequence variant in 8p-a3 strain.

(A)

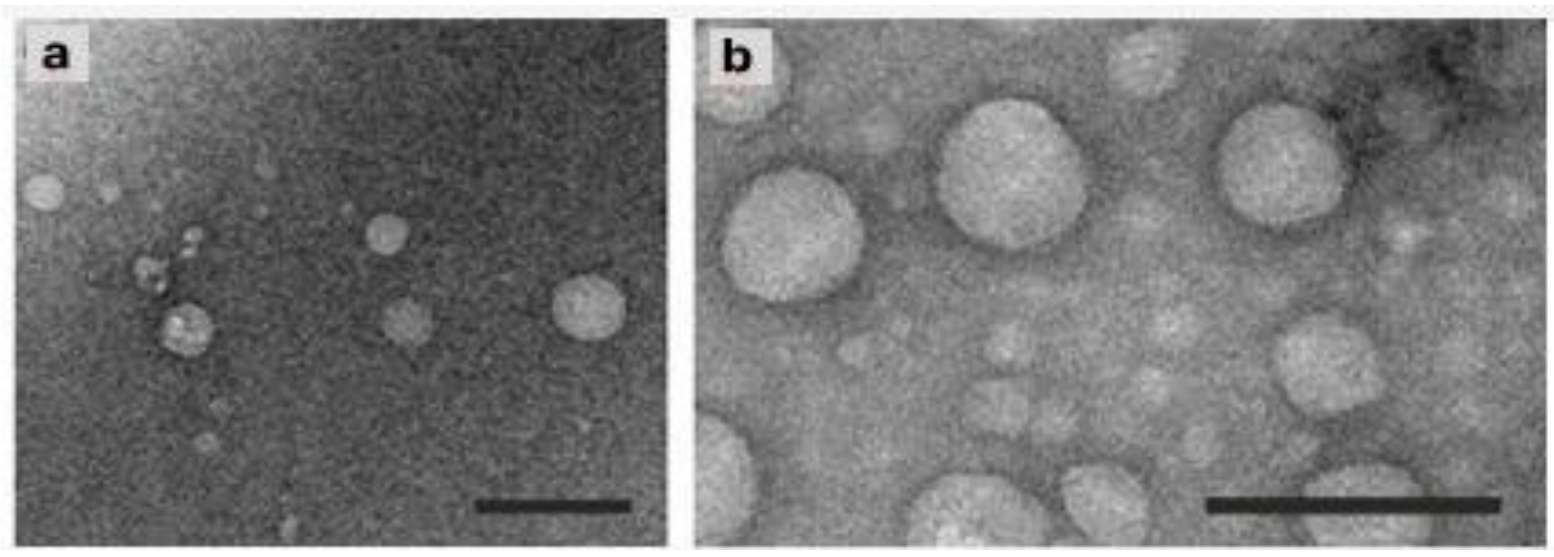

(B)

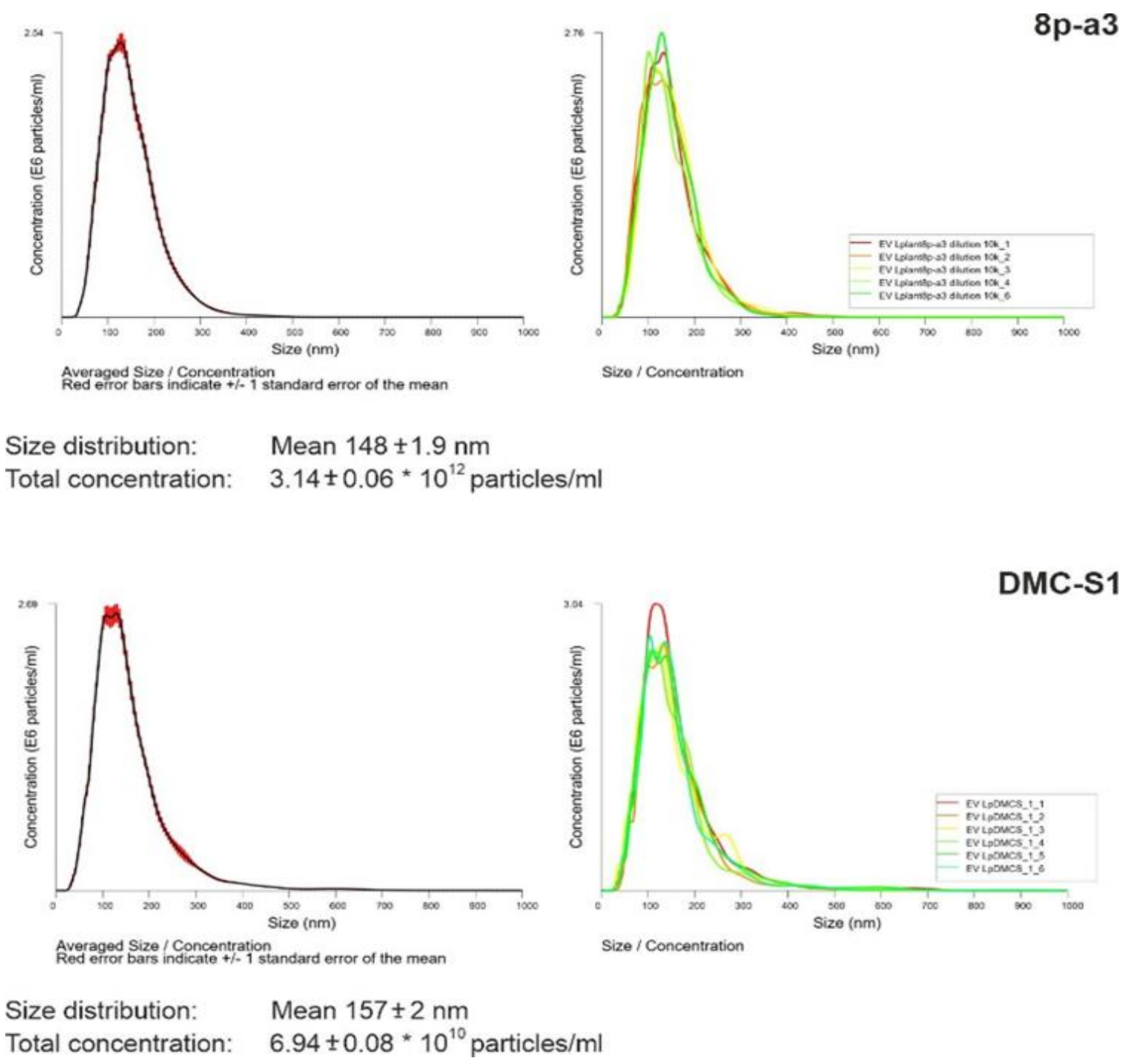

(C)

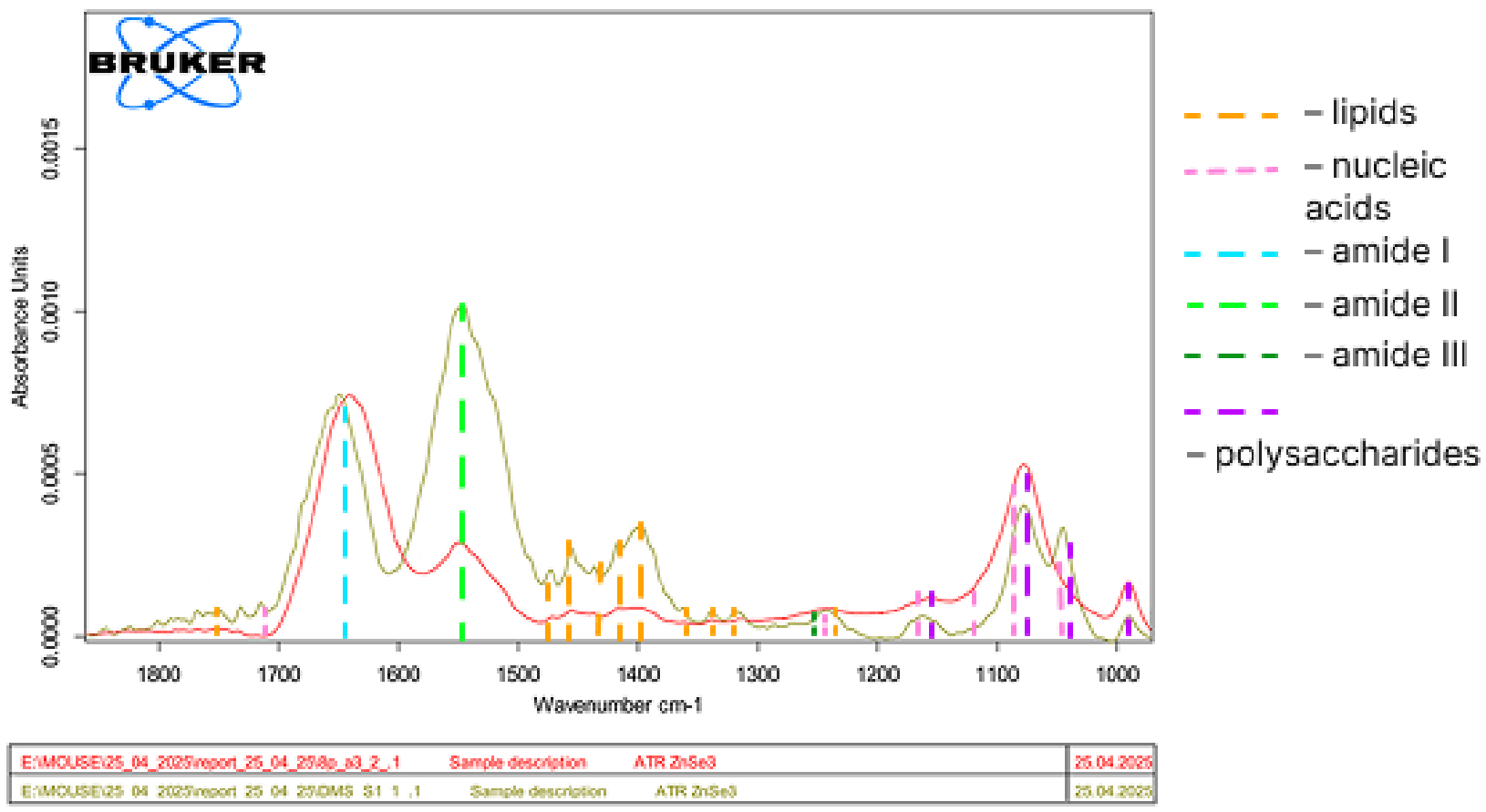

Fig. S10. Characterization of EVs from *L. plantarum* strains. (A) TEM micrographs (negative contrast) of EVs from *L. plantarum* strains 8p-a3 (a) and DMC-S 1 (b). (B) The scale bar represents 100 nm. Size distribution and concentration measured with NTA. The average values of particle sizes and concentrations are shown on the left, and data on technical repetitions of measurements are shown on the right. (C**)** IR spectrum of EVs samples. ATR-FTIR is a fast, label-free method for studying molecular composition without perturbing the biological samples. The baseline was subtracted for all spectra and the spectra were normalized to a maximum band of 1647 cm-1 (amide I).

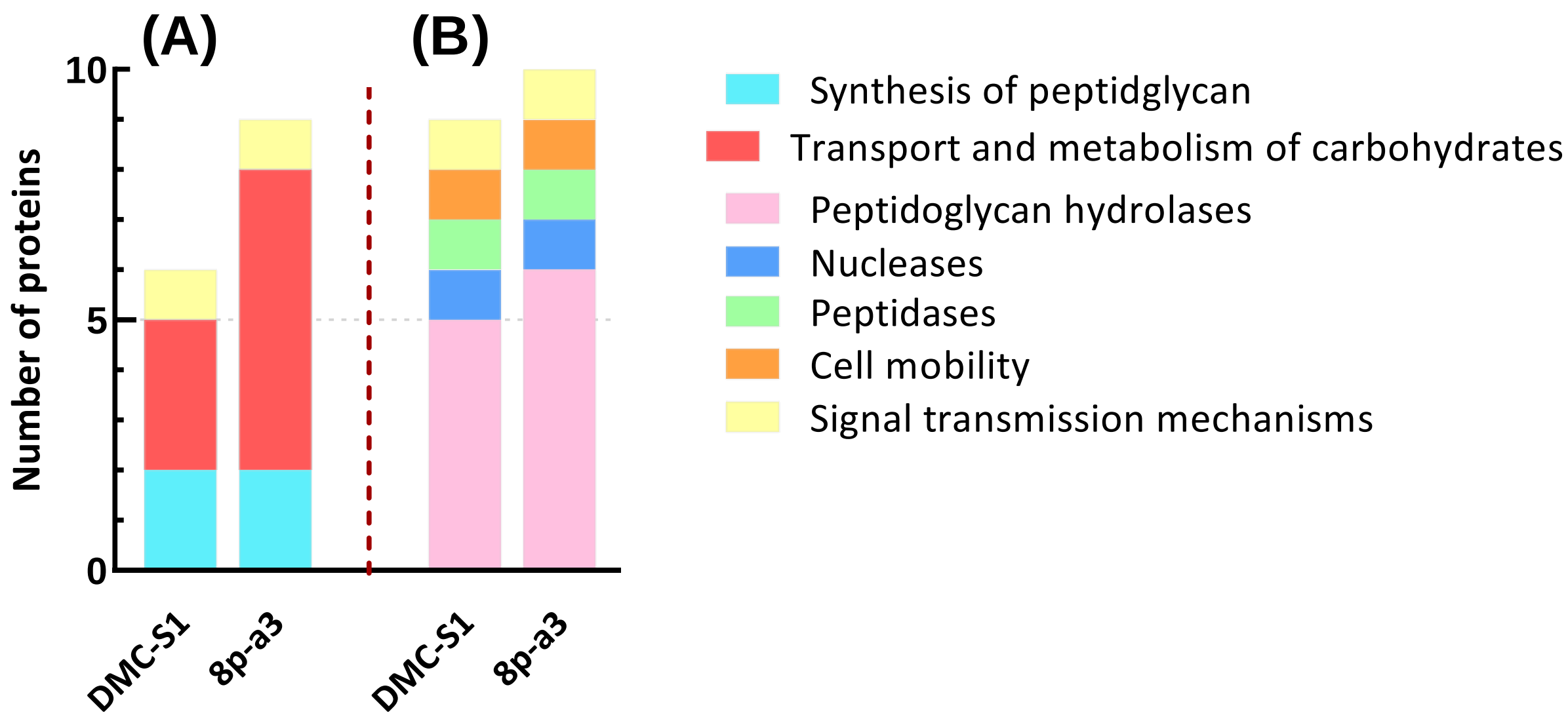

Fig. S11. The number of proteins in *L. plantarum* strains that may be involved in positive (A) and negative biofilm regulation (B).

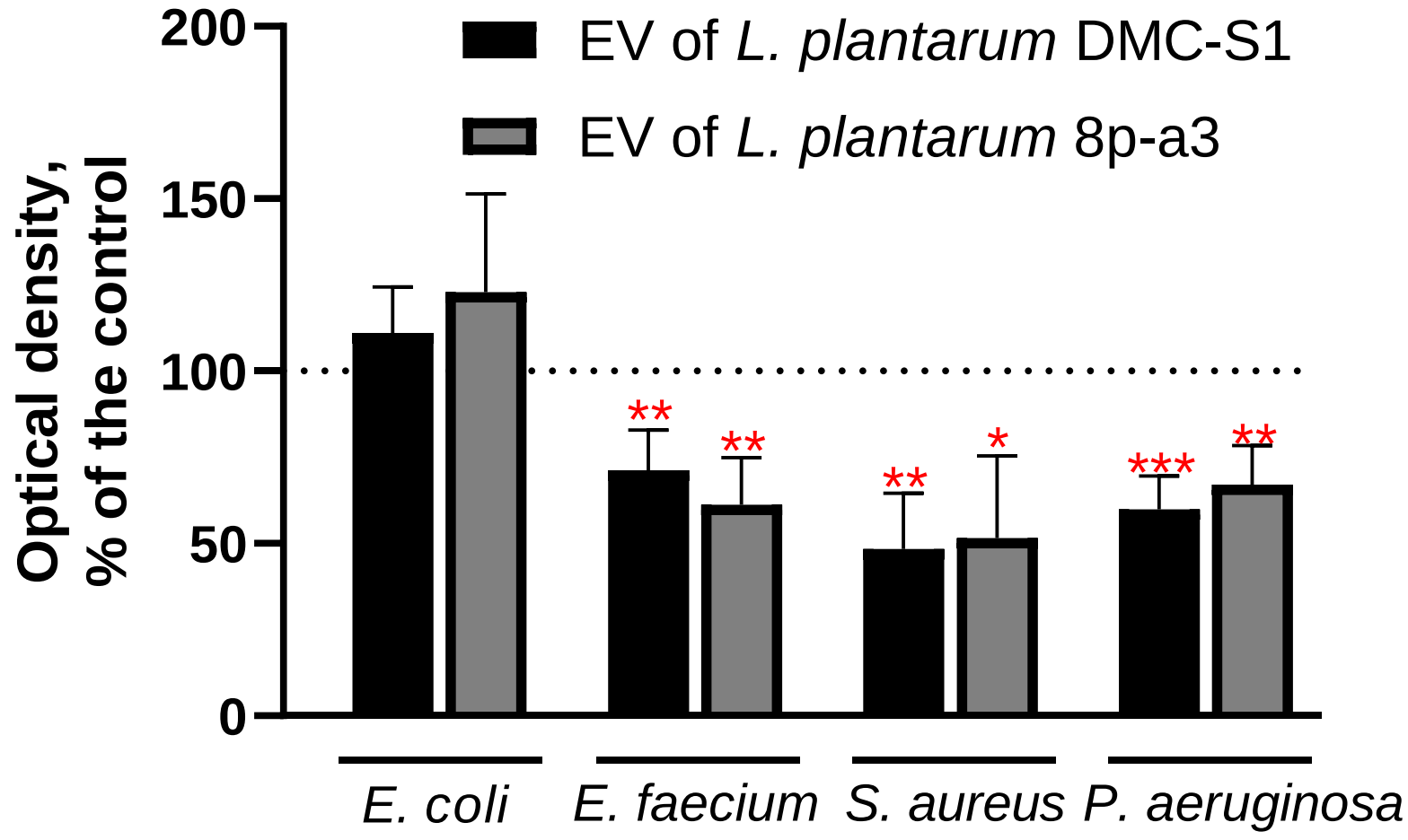

Fig. S12. The effect of extracellular vesicles of *L. plantarum* strains on the preformed biofilms of pathogens. The vesicles were added to preformed biofilms and after 24 h the biomass of the latter was quantified with CV-staining. Data are shown as percent of control (biofilms incubated in broth with no addition of extracellular vesicles of *L. plantarum* strains). Mean±SD is shown. Asterisk denote statistically significant difference with control biofilm of corresponding strain of pathogens (one-way ANOVA with Holm-Sidak post hoc test, n=4). *$p < 0.05$; **$p < 0.01$; *** $p < 0.001$.

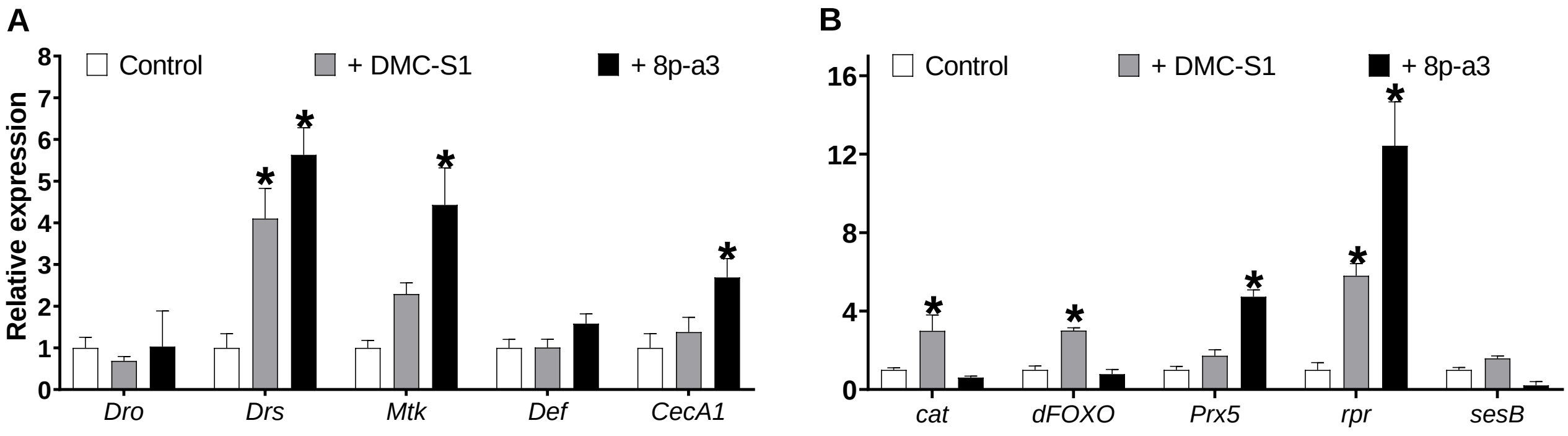

Fig. S13. Changes in the relative expression of genes encoding (A) AMP (*Cecropin* A1, *Defensin*, *Drosocin*, *Drosomycin* and *Metchnikowin*) and (B) stress response genes (*Catalase*, *dFoxo*, *Peroxiredoxin* 5, *reaper* and *sesB*) in *D. melanogaster* larvae treated with vesicles isolated from *L. plantarum* 8p-a3 and *L. plantarum* DMC-S1. Mean±SD is shown. Asterisk denote statistically significant difference with control (one-way ANOVA with Holm-Sidak post hoc test, n=4). *p < 0.05.

.

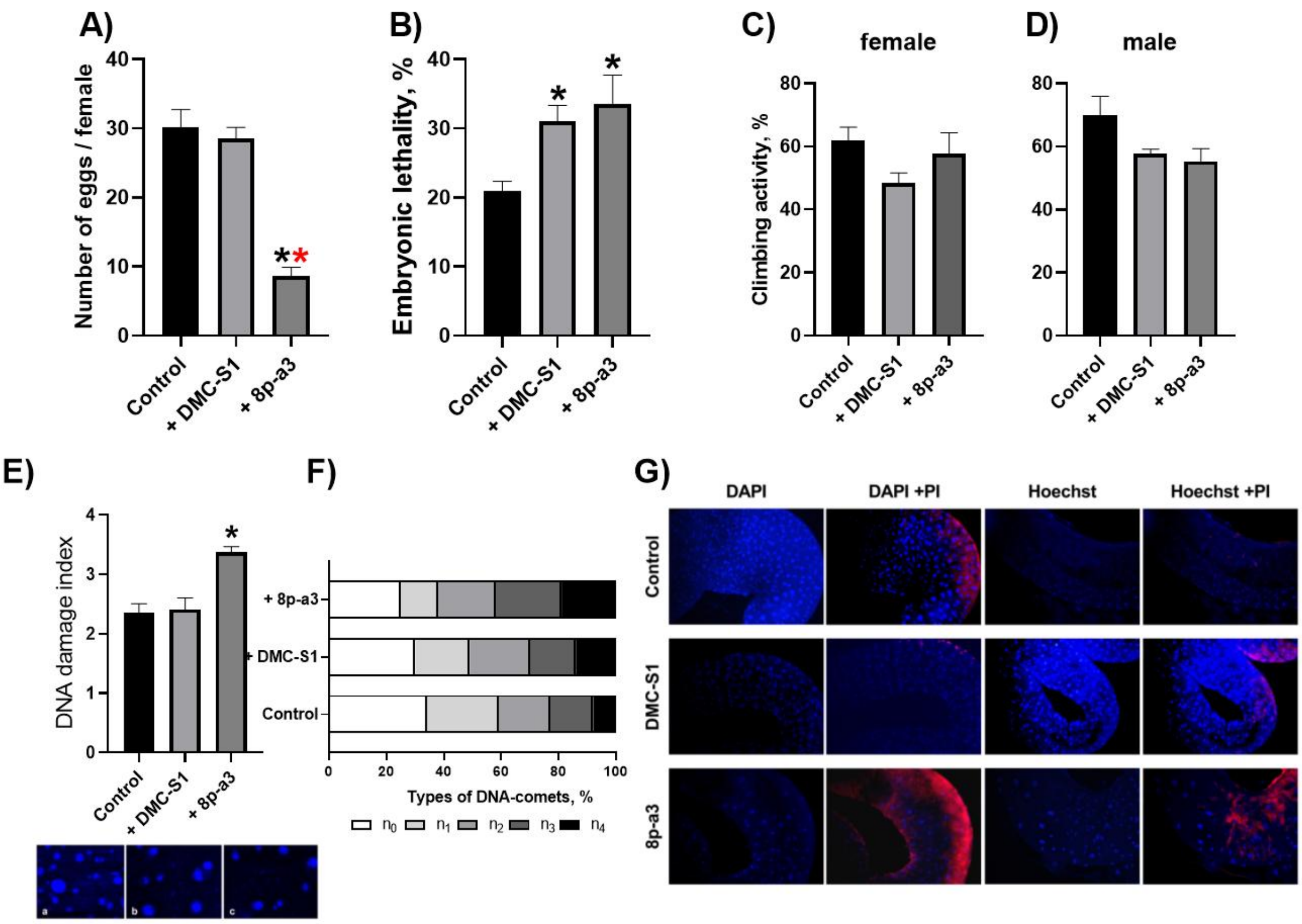

Fig. S14. The effects of vesicles from *L. plantarum* 8p-a3 and *L. plantarum* DMC-S1 on: a – egg production, b – embryonic death, c – motor activity, d – index of DNA damage in intestinal enterocytes, e – distribution of DNA comet types, f – visualization of intestinal tissue damage by *D. melanogaster*. Mean±SD is shown. Asterisk denote statistically significant difference with control (one-way ANOVA with Holm-Sidak post hoc test, n=4). *p < 0.05 **p < 0.01

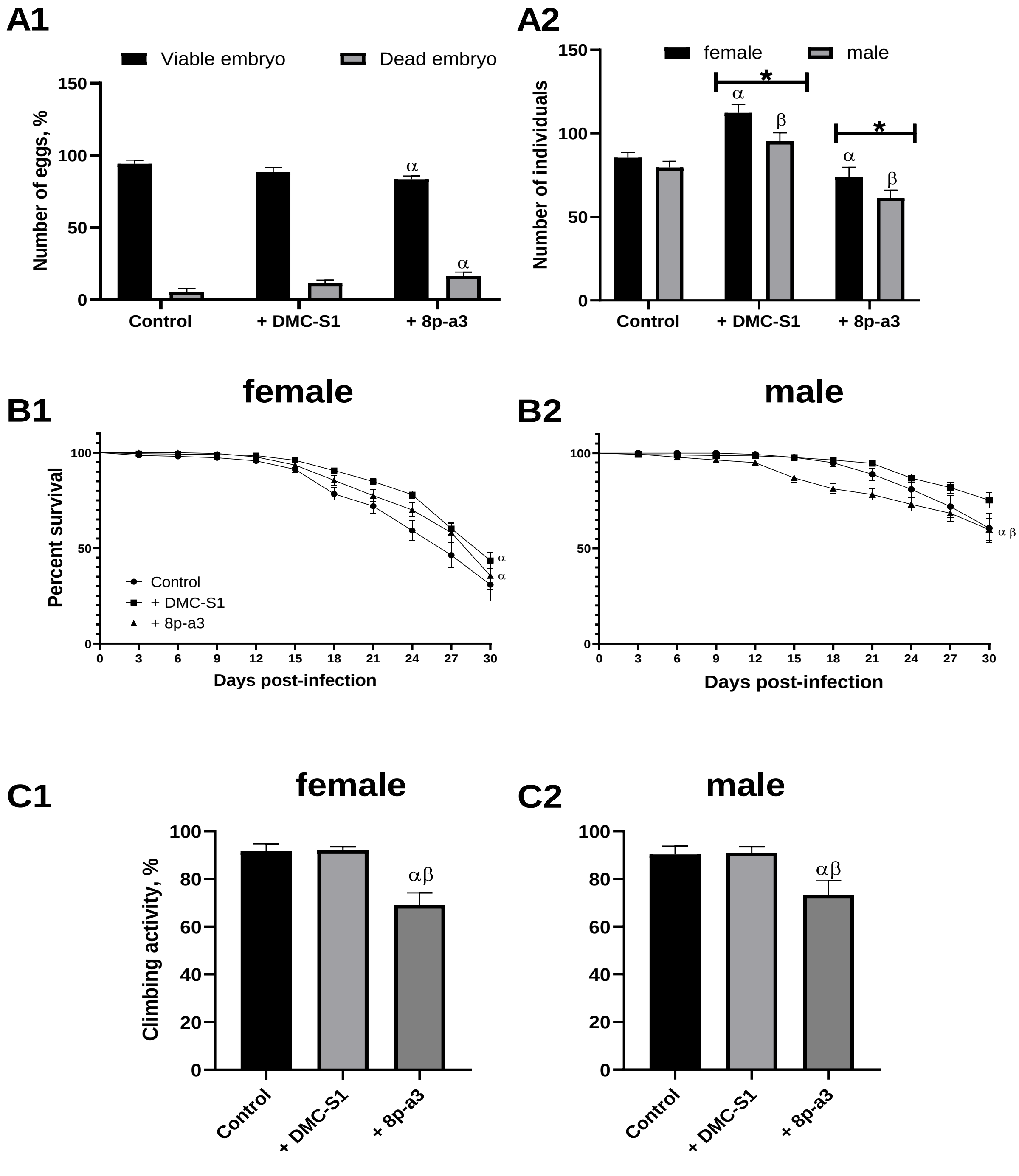

Fig. S15. The effect of *L. plantarum* strains on the adaptivity of flies in relation to A) the reproductive system (A1 – egg production and embryonic death; A2 – fertility – the number of viable offspring at the imago stage); C) survival of females (B1) and males (B2) after infection; C) the nervous system of females (C1) and males (C2). The data is presented in the form of an arithmetic mean and its error. The values were compared with each other using one-factor analysis of variance (one-way ANOVA) using a Bonferroni post hoc test; statistical analysis of data on the survival of flies was performed using the log-rank (Mantel-Cox) test: α, $P < 0.05$ compared to the group of uninfected flies; β, $P < 0.05$ compared to the group of flies infected with the strain *L. plantarum* DMC-S1.

A

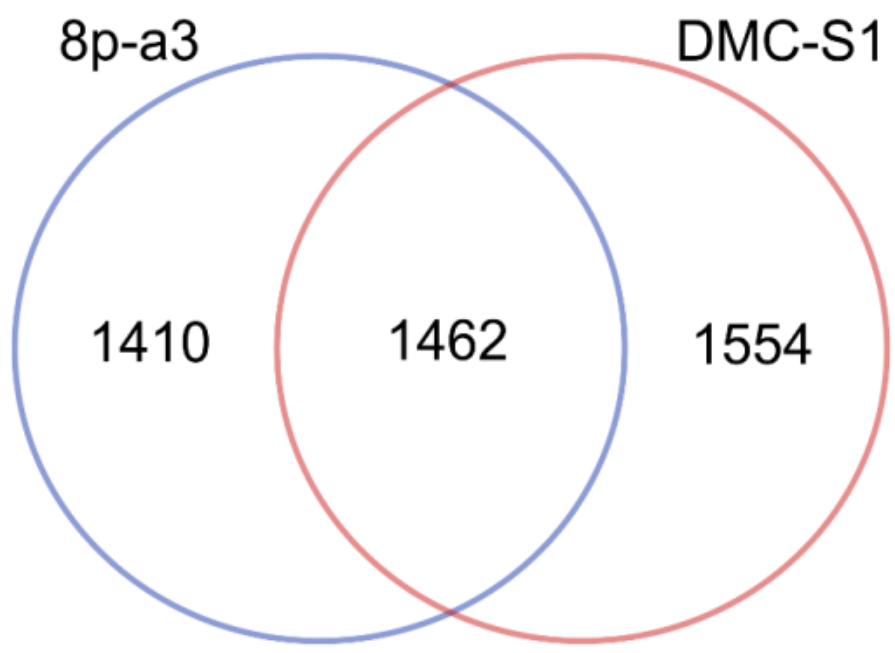

B

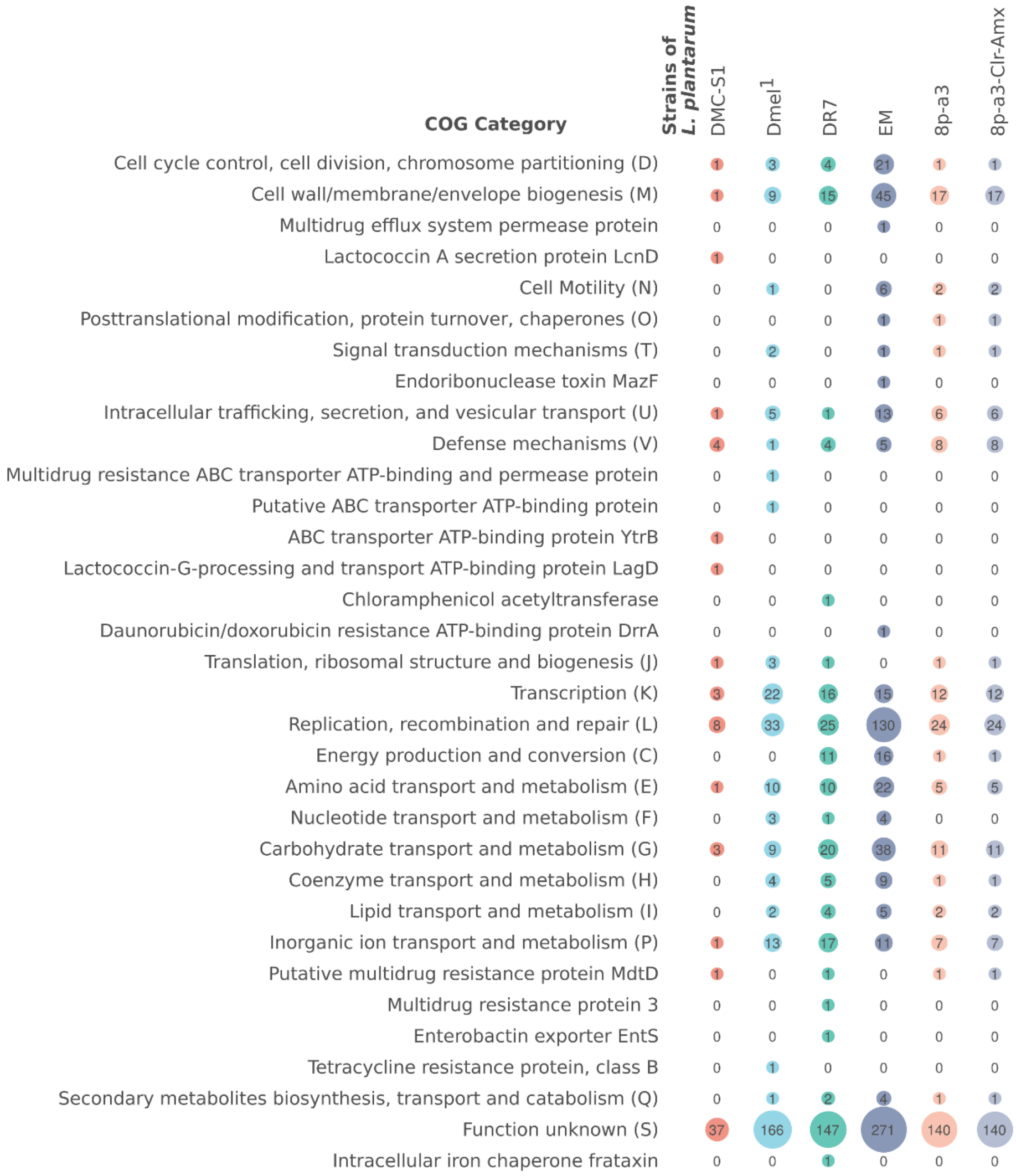

Fig. S16. A Venn diagram (A) and categorization of COG (B) proteins in *L. plantarum* strains (8p-a3, DMC-S1 and others). Genes of proteins with unknown function make up a significant part in the genomes of *L. plantarum* DMC-S1 and 8p-a3, including pools of common and unique genes of the strains.
